# Nanoparticles and single atoms of cobalt synergistically enabled low-temperature reductive amination of carbonyl compounds†

**DOI:** 10.1039/d2sc01596j

**Published:** 2022-07-08

**Authors:** Bingxiao Zheng, Jiao Xu, Jinliang Song, Haihong Wu, Xuelei Mei, Kaili Zhang, Wanying Han, Wei Wu, Mingyuan He, Buxing Han

**Affiliations:** Shanghai Key Laboratory of Green Chemistry and Chemical Processes, School of Chemistry and Molecular Engineering, East China Normal University Shanghai 200062 China hhwu@chem.ecnu.edu.cn hanbx@iccas.ac.cn; School of Chemical Engineering and Light Industry, Guangdong University of Technology Guangzhou 510006 China songjl_2021@gdut.edu.cn; Institute of Eco-Chongming Shanghai 202162 China; Beijing National Laboratory for Molecular Sciences, CAS Key Laboratory of Colloid, Interface and Chemical Thermodynamics, CAS Research/Education Center for Excellence in Molecular Sciences, Institute of Chemistry, Chinese Academy of Sciences Beijing 100190 China; School of Chemistry and Chemical Engineering, University of Chinese Academy of Sciences Beijing 100049 China

## Abstract

Low-temperature and selective reductive amination of carbonyl compounds is a highly promising approach to access primary amines. However, it remains a great challenge to conduct this attractive route efficiently over earth-abundant metal-based catalysts. Herein, we designed several Co-based catalysts (denoted as Co@C–N(*x*), where *x* represents the pyrolysis temperature) by the pyrolysis of the metal–organic framework ZIF-67 at different temperatures. Very interestingly, the prepared Co@C–N(800) could efficiently catalyze the reductive amination of various aldehydes/ketones to synthesize the corresponding primary amines with high yields at 35 °C. Besides non-noble metal and mild temperature, the other unique advantage of the catalyst was that the substrates with different reduction-sensitive groups could be converted into primary amines selectively because the Co-based catalyst was not active for these groups at low temperature. Systematic analysis revealed that the catalyst was composed of graphene encapsulated Co nanoparticles and atomically dispersed Co–N_*x*_ sites. The Co particles promoted the hydrogenation step, while the Co–N_*x*_ sites acted as acidic sites to activate the intermediate (Schiff base). The synergistic effect of metallic Co particles and Co–N_*x*_ sites is crucial for the excellent performance of the catalyst Co@C–N(800). To the best of our knowledge, this is the first study on efficient synthesis of primary amines *via* reductive amination of carbonyl compounds over earth-abundant metal-based catalysts at low temperature (35 °C).

## Abstract 2

An earth-abundant Co-based catalyst, Co@C–N(800), could efficiently catalyze the reductive amination of carbonyl compounds into primary amines at 35 °C owing to the synergistic effect of Co nanoparticles and atomically dispersed Co–N_*x*_ sites.

## Graphical abstract

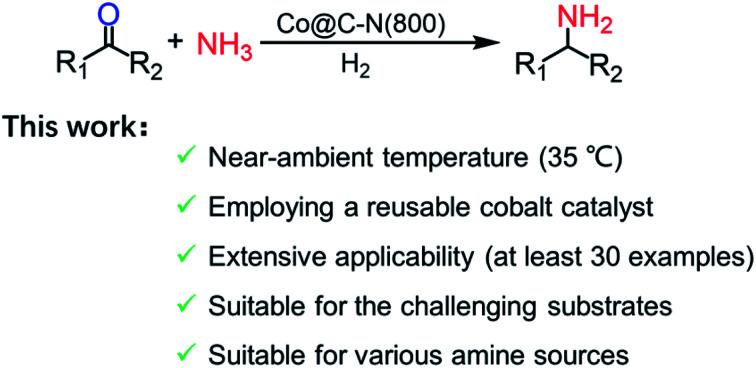

## Introduction

Primary amines represent a versatile class of compounds employed extensively as important intermediates to produce fine chemicals and functional materials.^1^ Generally, primary amines can be synthesized by several well-developed methods, including *N*-alkylation of ammonia with haloalkanes^2^ or alcohols,^3–5^ reductive amination of carbonyl compounds,^6–9^ and reduction of specific nitrogen-containing compounds.^10–13^ Among these synthetic methods, selective reductive amination of carbonyl compounds is the most promising strategy, which utilizes abundantly available NH_3_ and H_2_ as the alternatives of specific nitrogen-containing compounds and stoichiometric reductants, respectively. Notably, with the significant development of biorefineries, diverse carbonyl compounds can be obtained from transformation or direct extraction of renewable biomass.^14–20^ Besides, reductive amination often occurs with water as the major byproduct. These features afford reductive amination being a cost-effective, atom-efficient and environment-friendly approach to produce primary amines.

For reductive amination of carbonyl compounds with H_2_ as the reductant, metal-based catalysts are essential. Generally, noble metals (*i.e.*, Ru,^21–26^ Pt,^27,28^ Rh,^29,30^ and Pd (ref. 31)) show good performance on reductive amination of carbonyl compounds, even at ambient temperature (*e.g.*, Ru/TiP-100^24^ and dual-function Pd nanoparticles^31^). In spite of showing good activity, the high cost of noble metals significantly limited their practical applications. To address this drawback of noble metals, the catalysts from earth-abundant metals have attracted significant attention, and several earth-abundant metal (*i.e.*, Cu,^32^ Ni,^33–36^ and Co (ref. 37 and 38))-based catalysts have been developed for reductive amination of carbonyl compounds to synthesize primary amines. However, elevated temperatures are generally necessary for earth-abundant metals as the catalysts. Primary amines could be synthesized from reductive amination of carbonyl compounds over Cu/ZrO_2_ at 250 °C,^32^ metal–organic framework (MOF)-derived cobalt nanoparticles at 120 °C,^37^ Ni@SiO_2_ at 120 °C,^34^ or Ni/Al_2_O_3_ at 80 °C.^35^ Recently, Elfinger *et al.* performed the reductive amination of carbonyl compounds at 50 °C over the designed Co/N–SiC catalysts with a low loading of Co (1.5 mol%), but the yield of the desired amine from cyclohexanone was only 61% with a reaction time of 20 h.^39^ To date, reductive amination of carbonyl compounds at low temperature (<50 °C) over earth-abundant metals remains highly challenging. Additionally, the applications of earth-abundant metal-based catalysts are also limited by other shortcomings, including leaching, instability, easy inactivation, *etc*. Therefore, it is highly desirable for robust and stable earth-abundant metal-based catalysts (especially those with extensive substrate applicability) that enable reductive amination of carbonyl compounds to be performed at a reaction temperature of below 50 °C.

Herein, we reported the design of highly efficient and reusable cobalt-based catalysts by the pyrolysis of the metal–organic framework ZIF-67. The catalyst prepared at 800 °C, denoted as Co@C–N(800), showed excellent performance on reductive amination of various aldehydes/ketones using NH_3_ and H_2_ as the nitrogen and hydrogen resources, and a diverse range of primary amines with good to excellent yields could be successfully synthesized at 35 °C. More importantly, the feasibility of low (near ambient)-temperature reductive amination of aldehydes/ketones over earth-abundant metals was confirmed, which represented a crucial breakthrough in this important transformation. Besides, the wide applicability and the tolerance of other reducible groups further verified the great potential of this developed cobalt-based catalyst. As far as we know, this is the first time to achieve reductive amination of aldehydes/ketones to access primary amines over earth-abundant metals at such a low temperature (35 °C).

## Results and discussion

### Preparation and characterization of Co@C–N(*x*)

The Co@C–N(*x*) materials were prepared by a two-step process. First, the precursor ZIF-67 was synthesized from Co(NO_3_)_2_·6H_2_O and 2-methylimidazole (MeIM) by a hydrothermal method.^40^ The X-ray diffraction (XRD) pattern of the as-synthesized ZIF-67 was consistent with the reported results (Fig. S1†), verifying the successful formation of ZIF-67 crystals. Meanwhile, the synthesized ZIF-67 showed the typical rhombic dodecahedral shape with a particle size of around 500 nm (Fig. S2†). Subsequently, the desired Co@C–N(*x*) materials were obtained by direct pyrolysis of the as-synthesized ZIF-67 under a flow of nitrogen. Because the decomposition temperature of ZIF-67 was *ca.* 500 °C in a N_2_ atmosphere (Fig. S3†), the pyrolysis of ZIF-67 was performed at 600, 700, 800 and 900 °C, and the obtained materials are denoted as Co@C–N(*x*), where *x* represents the pyrolysis temperature.

**Fig. 1:**
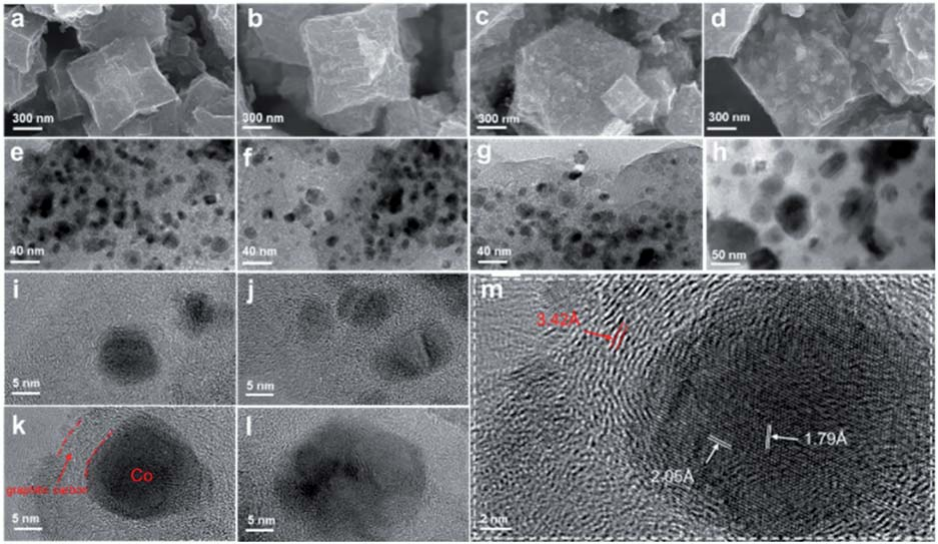
SEM images of (a) Co@C–N(600), (b) Co@C–N(700), (c) Co@C–N(800) and (d) Co@C–N(900); TEM images of (e) Co@C–N(600), (f) Co@C–N(700), (g) Co@C–N(800) and (h) Co@C–N(900); HR-TEM images of (i) Co@C–N(600), (j) Co@C–N(700), (k) Co@C–N(800), and (l) Co@C–N(900). The red lines in (k) indicate a Co nanoparticle wrapped with graphite layers. Panel (m) shows an enlarged image of a Co particle in (k).

The morphology and properties of the prepared Co@C–N(*x*) materials were examined by several typical techniques. The SEM images (Fig. 1a–d) indicated that there were obvious changes in the morphology of Co@C–N(*x*) in comparison with that of the parent ZIF-67 (Fig S2†), and the Co particles became clearer with the increase of the pyrolysis temperature from 600 to 900 °C, implying the gradual decomposition of the ZIF-67 frameworks. As characterized by TEM (Fig. 1e–h), the dispersion of Co particles was uniform. Although there was a slight increase in the sizes of Co particles (Fig. S4a–c†) when the pyrolysis temperature increased from 600 to 800 °C, no obvious aggregation of Co particles was observed. However, there was severe aggregation of Co particles in the material Co@C–N(900), and the particle size sharply increased to around 50 nm (Fig. S4d†). Furthermore, the HR-TEM images show that the Co particles in Co@C–N(600), Co@C–N(700) and Co@C–N(800) were surrounded tightly by graphitized carbon (Fig. 1i–l), and the graphitized carbon played the role of preventing the aggregation of Co particles, resulting in better dispersion of Co particles below 800 °C. The graphite-enclosed Co nanoparticles in Co@C–N(800) were highly crystallized (Fig. 1m) and exhibited two fringe spacings of 2.05 and 1.79 Å, corresponding to the (111) and (200) planes in fcc cobalt, respectively. In comparison, a large number of carbon nanotubes were formed (Fig. S5†) due to the catalytic effect of Co particles in Co@C–N(900), thus decreasing the graphitized carbon layer. Therefore, the protecting effect of the graphitized carbon layer on Co particles was weakened, resulting in severe aggregation of Co particles in Co@C–N(900). Additionally, EDS element mapping revealed the uniform distributions of C, N, and O and the dispersion of Co particles in the Co@C–N(*x*) materials (Fig. S6†).

**Fig. 2:**
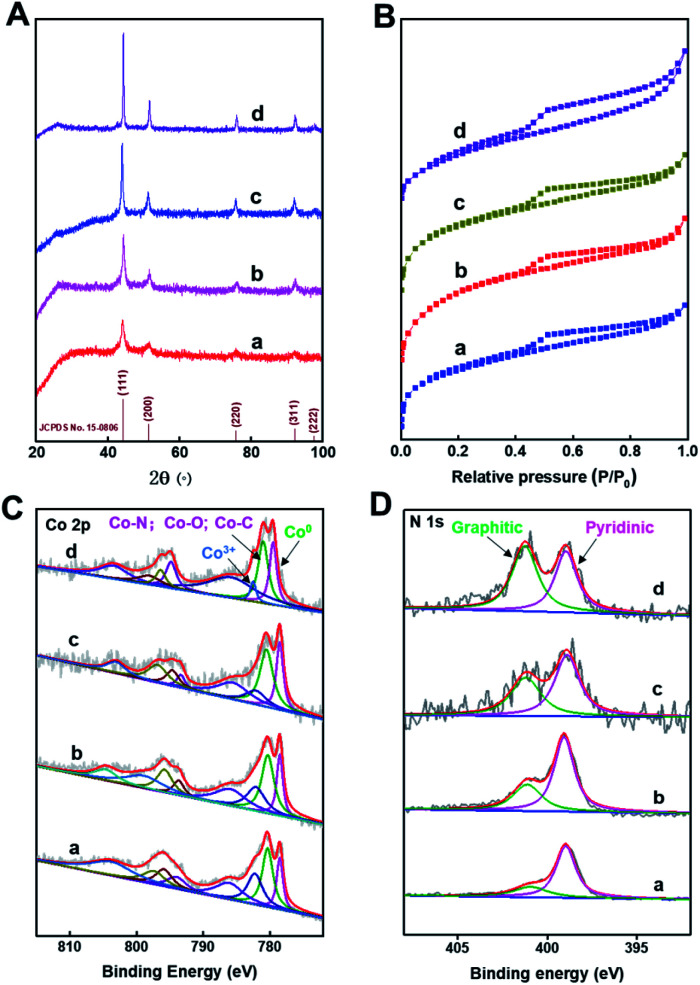
(A) Powder XRD patterns, (B) N_2_-adsorption–desorption isotherms, (C) Co 2p spectra and (D) N 1s spectra. In the figures, a, b, c, and d represent Co@C–N(600), Co@C–N(700), Co@C–N(800), and Co@C–N(900), respectively.

The XRD patterns (Fig. 2A) of Co@C–N(*x*) show five diffraction peaks at around 44.2, 51.6, 76.0, 92.4, and 97.8°, respectively, corresponding to the characteristic peaks of metallic Co (JCPDS no. 15-0806). The result indicated that Co^2+^ was reduced to generate metallic Co in all the four materials during the pyrolysis process. Meanwhile, from the XRD patterns, it could be concluded that the crystallinity of the metallic Co phase increased with the increase of pyrolysis temperature from 600 to 900 °C. The N_2_ adsorption–desorption isotherms of Co@C–N(*x*) show the mixed mode of type I and type IV (Fig. 2B), suggesting that Co@C–N(*x*) materials possessed both micropores and mesopores. Meanwhile, the BET surface area of the prepared Co@C–N(*x*) decreased with the increase of pyrolysis temperature from 600 to 900 °C (Table S1†), and the pore size showed no obvious difference (Table S1†). The fine structure of Co@C–N(*x*) was further characterized by X-ray photoelectron spectroscopy (XPS). The XPS spectra of Co 2p show that there were three Co species in all the four Co@C–N(*x*) materials, including metallic Co^0^ at 778.5 eV, Co–O/Co–N/Co–C at 780.5 eV, and Co^3+^ at 782.1 eV (Fig. 2C). In comparison with the XPS spectrum of Co 2p in ZIF-67 (Fig. S7A†), the presence of Co^0^ (778.5 eV) in Co@C–N(*x*) implied the partial reduction of cobalt during the pyrolysis process, which was consistent with the results of XRD examinations (Fig. 2A). Moreover, in the XPS spectra of N 1s, a peak at 401.0–402.0 eV appeared in all the Co@C–N(*x*) materials (Fig. 2D), which could be assigned to the graphitic-N species. In contrast, only pyridinic-N species at 398.7 eV were observed in the precursor ZIF-67 (Fig. S7B†). The ratio of graphitic-N/pyridinic-N in the Co@C–N(*x*) materials increased with the increase of pyrolysis temperature, *e.g.*, 0.35 for 600 °C, 0.5 for 700 °C, 0.75 for 800 °C, and 1.2 for 900 °C, implying that the graphitization degree was enhanced at higher temperatures. Additionally, as determined by ICP, the content of Co in the obtained Co@C–N(*x*) increased with the increase of pyrolysis temperature (Table S2†), suggesting the gradual decomposition of the organic components.

### Catalytic performance of various catalysts

The catalytic performance (activity and selectivity) of the synthesized Co@C–N(*x*) materials was initially evaluated by selecting the reductive amination of cyclohexanone to cyclohexylamine as a model reaction (Table 1). The desired cyclohexylamine was not generated in the catalyst-free system (Table 1, entry 1). Meanwhile, the precursor ZIF-67 showed no catalytic activity for the reductive amination of cyclohexanone to synthesize cyclohexylamine (Table 1, entry 2). Interestingly, the materials prepared from the pyrolysis of ZIF-67 could catalyze the reaction to obtain cyclohexylamine (Table 1, entries 3–6). The selectivity to cyclohexylamine increased with the increase of pyrolysis temperature from 600 to 800 °C (Table 1, entries 3–6), and Co@C–N(800) exhibited the highest catalytic efficiency with a cyclohexanone conversion of >99% and a cyclohexylamine selectivity of 96% (Table 1, entry 5). However, when the pyrolysis temperature further increased to 900 °C, the cyclohexylamine selectivity decreased (Table 1, entry 6). The main reason was that the Co particles in Co@C–N(900) were much larger as discussed above. These results indicated the significantly important role of pyrolysis temperature in the catalytic performance. Moreover, when the usage of Co@C–N(800) increased from 40 (23.4 mol% Co) to 50 mg (29.2 mol% Co), both cyclohexanone conversion and cyclohexylamine selectivity were >99% (Table 1, entry 7). The above discussion indicated that Co@C–N(800) was the optimal catalyst for the reductive amination of cyclohexanone.

**Table 1:** Activity of various catalysts for reductive amination of cyclohexanone^a^

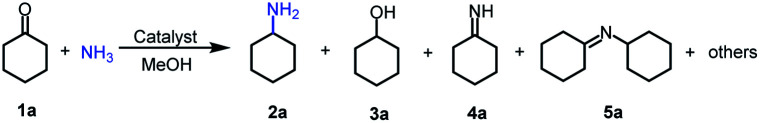							
Entry	Catalyst	Conv. (%)	Selectivity^b^ (%)				
			2a	3a	4a	5a	others
1	Blank	90	0	0	99	0	1
2	ZIF-67	91	0	0	99	0	1
3	Co@C–N(600)	99	50	0	0	50	0
4	Co@C–N(700)	98	80	0	0	20	0
5	Co@C–N(800)	>99	96	0	0	4	0
6	Co@C–N(900)	98	90	0	0	10	0
7^c^	Co@C–N(800)	>99	>99	0	0	0	0
8^d^	Co@C–N(800)	>99	95	0	0	5	0
9^e^	Co@C–N(800)	>99	>99	0	0	0	0
10^f^	Co@C–N(800)	>99	>99	0	0	0	0
11	Co@C–N(800)–H^+^	92	0	0	3	7	91
12	Co@C–N(800)–air	98	9	0	0	91	0
13	Co/C	88	0	0	95	0	5

a Reaction conditions: cyclohexanone, 1 mmol; methanol, 3 mL; H_2_, 1.4 MPa; NH_3_, 0.6 MPa; catalyst, 40 mg (the molar usage of Co was 18.4, 22.9, 23.4, or 25.3 mol% for entries 3–6, respectively); 35 °C; 6 h.

b The conversion and selectivity were determined by GC using 1-butanol as a standard.

c Catalyst, 50 mg (29.2 mol% Co).

d Catalyst, 10 mg (5.8 mol% Co); 24 h.

e Catalyst, 10 mg (5.8 mol% Co); 50 °C; 12 h.

f Catalyst, 14 mg (8.0 mol% Co); 24 h.

### Optimization of reaction conditions

**Fig. 3:**
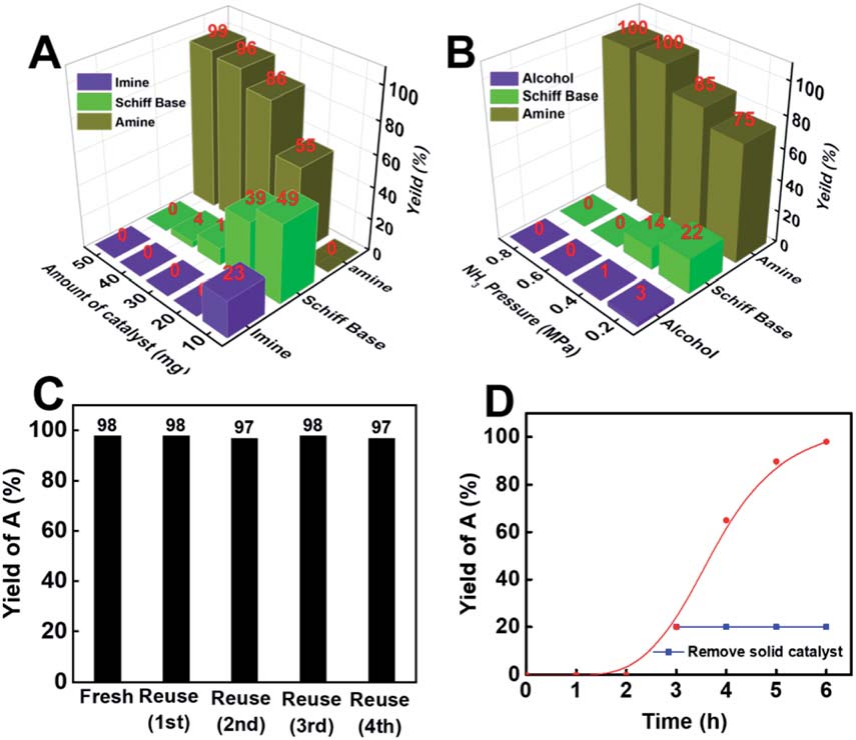
(A) Effect of the catalyst amount, (B) effect of NH_3_ pressure, (C) reusability of Co@C–N(800), and (D) time–yield plots for reductive amination of cyclohexanone with Co@C–N(800) (red line) or removing Co@C–N(800) after 3 h (blue line). Reaction conditions: cyclohexanone, 1 mmol; methanol, 3 mL; 35 °C; 6 h; Co@C–N(800), 50 mg (29.2 mol% Co); total pressure of NH_3_ and H_2_ was 2 MPa and 1.4 MPa H_2_ and 0.6 MPa NH_3_ in part D.

Employing Co@C–N(800) as the catalyst, the effects of the catalyst amount and the ratio of H_2_ and NH_3_ on cyclohexanone conversion and product selectivity were subsequently investigated (Fig. 3). First, the yield of cyclohexylamine increased with the increase of the Co@C–N(800) amount from 10 (5.8 mol% Co) to 50 mg (29.2 mol% Co) (Fig. 3A). Meanwhile, the product distribution was significantly affected by the amount of Co@C–N(800) (Fig. 3A). When the usage of Co@C–N(800) was too low (*e.g.*, 10 mg with 5.8 mol% Co), the main products were Schiff base and imine rather than the desired cyclohexylamine, resulting from the insufficient active sites at lower catalyst usage, and prolonged reaction time was needed to yield the desired cyclohexylamine. For example, a cyclohexylamine yield of 95% could be achieved over 10 mg Co@C–N(800) at 35 °C with a reaction time of 24 h (Table 1, entry 8). With the increase in the usage of Co@C–N(800), the yield of cyclohexylamine obviously increased and the amount of Schiff base decreased, while the imine would be undetected with a catalyst usage of >10 mg (5.8 mol% Co). From the viewpoint of achieving high conversion and product selectivity simultaneously, 50 mg (29.2 mol% Co) was selected for the following investigations. Besides, we found that cyclohexanone could also be quantitively converted into cyclohexylamine over 10 mg Co@C–N(800) (about 5.8 mol% Co) at 50 °C with a reaction time of 12 h (Table 1, entry 9) or over Co@C–N(800) with a Co usage of 8 mol% (about 14 mg catalyst) at 35 °C when the reaction time was prolonged to 24 h (Table 1, entry 10). These results confirmed that the reductive amination over Co@C–N(800) was a Co-catalyzed rather than a Co-mediated reaction. Second, the ratio of H_2_ and NH_3_ significantly affected the product distribution over Co@C–N(800) (Fig. 3B), and the best H_2_/NH_3_ ratio for our catalytic system was observed to be 7/3. When the H_2_/NH_3_ ratio was changed from 9/1 to 7/3, the selectivity of cyclohexylamine increased from 75% to 99%, because a high NH_3_ concentration could promote the subsequent conversion of the formed Schiff base to the desired product, which would be discussed in the following section. Further lowering the H_2_/NH_3_ ratio to 3/2, the selectivity of cyclohexylamine (99%) remained similar to that at a H_2_/NH_3_ ratio of 7/3. Additionally, at a high H_2_/NH_3_ ratio (*e.g.*, 9/1), cyclohexanol would be generated from direct hydrogenation of cyclohexanone. Thus, 7/3 was the optimal H_2_/NH_3_ ratio in our catalytic system.

### Stability of Co@C–N(800)

As is well-known, the stability of earth-abundant metal-based catalysts, which suffers from leaching and aggregation during the reaction process, is generally poor. Thus, evaluation of the durability of Co@C–N(800) was a significant aspect. To our delight, Co@C–N(800) could be reused for at least five catalytic cycles without considerable changes in catalytic performance (Fig. 3C). As characterized by SEM, TEM, XPS and XRD (Fig. S8 and S9†), no obvious difference was found between the virgin and recovered Co@C–N(800). These results indicated the high stability of Co@C–N(800). Moreover, the yield of cyclohexylamine plateaued after Co@C–N(800) was removed from the reaction system after 3 h (Fig. 3D), and the leaching of the Co species was negligible based on the results of ICP examination (Table S1†), firmly verifying the heterogeneous nature of Co@C–N(800). Additionally, Co@C–N(800) could be easily separated with a magnet quickly (Fig. S10†) because of its good magnetic properties.

### Discussion on the reasons for the high activity of Co@C–N(800)

It is widely accepted that pyrolysis of ZIF-based MOFs generally leads to the formation of metal-N-doped carbon materials with high structural heterogeneity. These materials are typically composed of atomically dispersed metal-N_*x*_ sites (metal single-atoms) and graphene encapsulated metal nanoparticles, which are both potential active sites. Herein, several experiments were conducted to identify the catalytically active sites of Co@C–N(800) for reductive amination of cyclohexanone. First, Co@C–N(800) was washed in an aqueous solution of HCl (36.0 wt%) to remove Co nanoparticles with the preservation of Co–N_*x*_ sites (Table S2†), and the resultant material was denoted as Co@C–N(800)–H^+^, which was characterized by Fourier transformed (FT) *k*^3^-weighted extended X-ray absorption fine structure (EXAFS), atomic-resolution high-angle annular dark-field scanning transmission electron microscopy (HAADF-STEM), XRD, and XPS. The EXAFS spectra (Fig. S11†) show that there was a strong Co–N coordination peak (at about 1.46 Å), while the Co–Co coordination peak (at about 2.18 Å) was weak. These results indicated the existence of Co–N_*x*_ sites and the removal of most of the Co nanoparticles from Co@C–N(800). The HAADF-STEM results clearly indicate that Co@C–N(800)–H^+^ contained Co single atoms (Fig. S12A†). Additionally, EDS element mapping revealed the uniform distributions of C, N, and O and the dispersion of the Co species in Co@C–N(800)–H^+^ (Fig. S12B–F†). Meanwhile, Co nanoparticles could still be examined by XRD (Fig. S13†) and XPS analysis (Fig. S14†). The content of Co nanoparticles significantly decreased based on the decreased area ratio of Co^0^ in the Co XPS spectra (Fig. S14A†), and thus an obvious characteristic peak of C (002) was observed in the XRD pattern of Co@C–N(800)–H^+^ (Fig. S13B†). It is known that Co nanoparticles could be easily removed using HCl solution.^41^ Thus, it can be deduced that the remaining Co nanoparticles in Co@C–N(800)–H^+^ (the treated catalyst with HCl solution for 48 h) were wrapped in the C–N support. We can assume that they could not play a catalytic role in the reaction process because it was very difficult for them to contact with the reactants due to the limited mass transfer.^41^ These results above confirmed the coexistence of metallic Co and atomically dispersed Co–N_*x*_ sites in Co@C–N(800). When using Co@C–N(800)–H^+^ as the catalyst, only very small amounts of Schiff bases were generated (Table 1, entry 11), indicating poor catalytic activity of Co@C–N(800)–H^+^ in the hydrogenation step of the reaction process.^41^ Second, the C–N composite could be removed by treating Co@C–N(800) in air at 400 °C for 1 h and then 1 h under a H_2_/Ar atmosphere (containing 90% Ar) (Table S2†), and the resultant material was denoted as Co@C–N(800)–air.^40^ The XPS spectra of Co@C–N(800)–air indicated that the signal of N was very weak (Fig. S13D†), implying that atomically dispersed Co–N_*x*_ sites were almost completely removed in Co@C–N(800)–air. The activity of Co@C–N(800)–air was significantly decreased, and the yield of cyclohexylamine was only 9.5% with a large amount (91%) of the Schiff base at 35 °C (Table 1, entry 12), indicating that the catalytic ability of Co@C–N(800)–air was weak for further conversion of the Schiff base into the desired product. As reported, acidic sites can promote further conversion of the Schiff base in the presence of H_2_ and NH_3_. Thus, the removed Co–N_*x*_ species may play the role of acidic sites in the reaction process. Additionally, Co supported on activated carbon (Co/C) showed no activity for the formation of cyclohexylamine and even Schiff base (Table 1, entry 13) because of its very weak ability to activate C=N in imine for further conversion. These control experiments suggested that both metallic Co (for hydrogenation step) and atomically dispersed Co–N_*x*_ species (acting as acidic sites) were indispensable for the reaction. The synergistic effect of metallic Co and Co–N_*x*_ sites is crucial for the excellent performance of Co@C–N(800).

As discussed above, the pyrolysis temperature significantly affected the catalytic activity of the prepared materials. Considering that the catalytically active sites were the Co species, the properties of the involved materials were analyzed by XPS examinations. The XPS spectra of Co 2p clearly show that no metallic Co existed in ZIF-67 (Fig. S7A†), while metallic Co was observed in other materials prepared by pyrolysis (Fig. 3A). Because metallic Co was the catalytic site for the hydrogenation step, ZIF-67 was inactive for the studied reaction, while all the pyrolyzed materials showed obvious activity. Meanwhile, as can be seen from the XPS spectra of Co 2p and the ICP results, the content of metallic Co in the pyrolyzed materials increased with the increase of pyrolysis temperature (Table S2†) because higher pyrolysis temperature could promote more Co^2+^ to be reduced. The binding energy of Co 2p (for metallic Co) followed the order: Co@C–N(800) < Co@C–N(700) < Co@C–N(600) < Co@C–N(900). The lower binding energy indicated the formation of more negatively charged metallic Co, which was helpful for activating H_2_ to attend the hydrogenation steps (Fig. S15†).^25^ Thus, Co@C–N(800) with a sufficient amount of more negatively charged metallic Co possessed the best catalytic performance. Although Co@C–N(900) had more content of metallic Co, the lower electron density and serious aggregation of Co particles (Fig. 1h) resulted in poorer ability to activate H_2_ and lower utilization efficiency of metallic Co, thus making the activity of Co@C–N(900) be lower than that of Co@C–N(800). From another aspect, the binding energy for Co–N_*x*_ species increased with the increase of pyrolysis temperature: Co@C–N(900) > Co@C–N(800) > Co@C–N(700) > Co@C–N(600), indicating that the Co–N_*x*_ species in Co@C–N(900) were more positively charged, affording it to possess stronger Lewis acidity. Thus, Co@C–N(900) had the highest ability to promote further conversion of the Schiff base *via* the interaction between the Co–N_*x*_ sites and the N atom in the C=N bonds (Fig. S16†).^42,43^ The strong ability of Co–N_*x*_ sites in Co@C–N(900) to activate C=N bonds could make up its weak ability to activate H_2_, thus resulting in better performance of Co@C–N(900) than both Co@C–N(600) and Co@C–N(700).

From the discussion above, we could deduce that the cooperation of atomically dispersed Co–N_*x*_ sites and metallic Co particles was crucial for the excellent catalytic performance of the prepared catalysts. The EXAFS spectra (Fig. S11†) of Co@C–N(800) show that there was a strong Co–Co coordination peak (at about 2.18 Å), resulting in a weak Co–N coordination peak (at about 1.46 Å), which is a general phenomenon when metal nanoparticles and signal metal atoms simultaneously exist.^41^ As discussed above, the Co–N coordination peak was significantly improved after most of the Co nanoparticles were removed (Fig. S11†). These results confirmed the coexistence of metallic Co and atomically dispersed Co–N_*x*_ sites. Metallic Co was the catalytic site for hydrogenation step, while Co–N_*x*_ species as Lewis acidic sites could promote conversion of the intermediate (Schiff base) in the presence of H_2_ and NH_3_. Co@C–N(800), which contained sufficient content of metallic Co and Co–N_*x*_ species in a suitable electronic state, showed the best catalytic performance for the reductive amination.

### Substrate scope

Based on the above results, the applicability of Co@C–N(800) in reductive amination of other ketones was investigated (Table 2). All the examined alkyl ketones (Table 2, entries 1–9), including 2-adamantanone with a complex spatial structure (Table 2, entry 9), could be efficiently converted into the corresponding primary amines with good yields over Co@C–N(800) at 35 °C. Although acetophenone showed lower reactivity at 35 °C (Table 2, entry 10) due to the electronic and steric effects of the phenyl group, the yield of 1-phenylethylamine could reach 95% by increasing the reaction temperature to 60 °C and changing the H_2_ pressure (0.4 MPa H_2_ in a total pressure of 1 MPa).

**Table 2:** Reductive amination of various ketones over Co@C–N(800)^a^

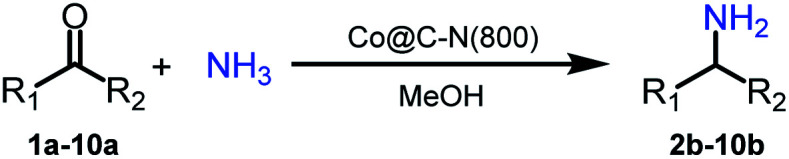			
Entry	Ketones	Products	Yields^b^^,^^g^ (%)
1	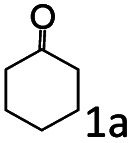	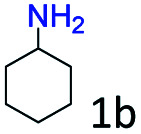	99 (96)
2^c^	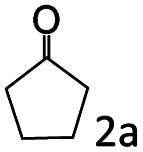	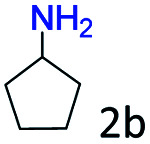	94 (92)
3^c^	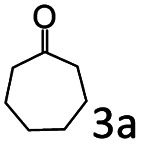	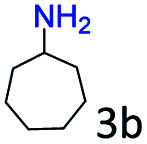	97 (94)
4^c^	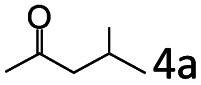	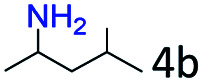	92 (87)
5^d^	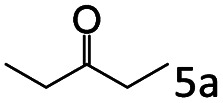	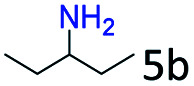	94 (90)
6^c^	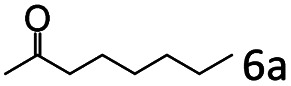	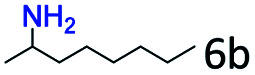	93 (92)
7^c^	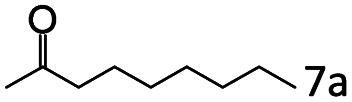	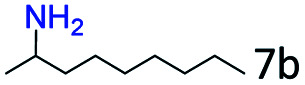	92 (90)
8^d^	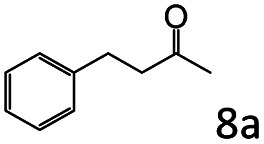	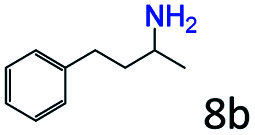	98 (95)
9^e^	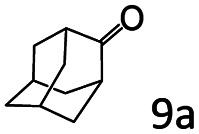	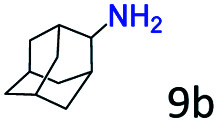	96 (92)
10^e^	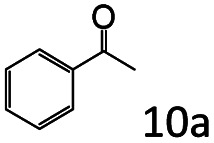	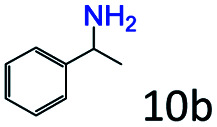	42
11^f^	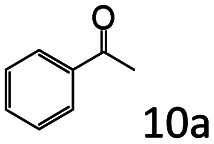	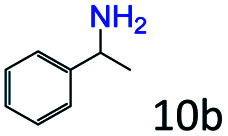	95 (91)

a Reaction conditions: ketone, 1 mmol; methanol, 3 mL; H_2_, 1.4 MPa; NH_3_, 0.6 MPa; Co@C–N(800), 50 mg (29.2 mol% Co); 35 °C; 6 h.

b The conversion and selectivity were determined by GC using 1-butanol as a standard.

c 13 h.

d 20 h.

e 24 h.

f 60 °C, 30 h.

g Isolated yield is shown in parentheses.

Inspired by the good applicability of Co@C–N(800) in the reductive amination of ketones, we attempted to employ it to catalyze reductive amination of aldehydes, which was more challenging to selectively form primary amines because of the higher reactivity of aldehydes in comparison with ketones. To our delight, Co@C–N(800) also showed high activity for the reaction with a slightly modified NH_3_/H_2_ ratio (1.7 MPa H_2_ and 0.3 MPa NH_3_ at a total pressure of 2 MPa) at 35 °C (Table 3), and the corresponding primary amines could be synthesized with high selectivity. Besides, the reaction conditions (35 °C and H_2_ pressure of <2 MPa) over Co@C–N(800) were milder than those in reported earth-abundant metal-based catalytic systems or most of noble-metal-based systems (Table S3†). Importantly, the activity and product selectivity over Co@C–N(800) (from the usage of metal, TON, TOF, and yield of the desired product) was better than those over reported earth-abundant metal-based catalytic systems. The results above confirmed the superior advantage of Co@C–N(800) for the synthesis of primary amines *via* the reductive amination of ketones and aldehydes.

**Table 3:** Reductive amination of various aldehydes over Co@C–N(800)^a^

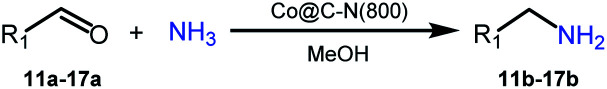			
Entry	Aldehydes	Products	Yields^b^^,^^e^ (%)
1	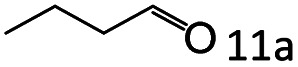	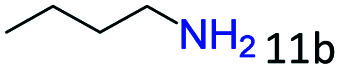	95 (90)
2	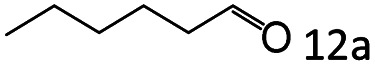	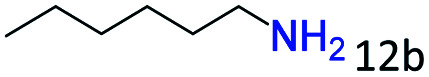	90 (87)
3	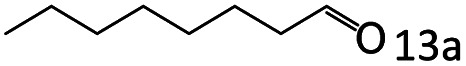	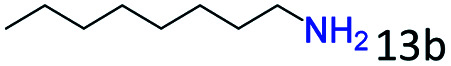	91 (86)
4	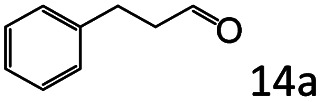	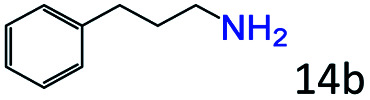	90 (89)
5^c^	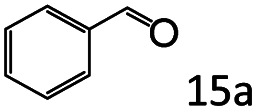	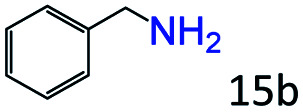	98 (92)
6^d^	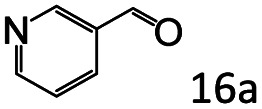	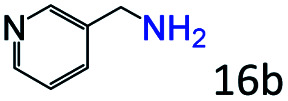	97 (93)
7	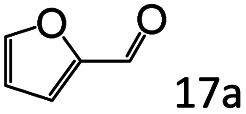	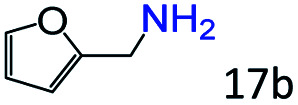	70 (64)

a Reaction conditions: ketone, 1 mmol; methanol, 3 mL; H_2_, 1.7 MPa; NH_3_, 0.3 MPa; Co@C–N(800), 50 mg (29.2 mol% Co); 35 °C; 24 h.

b The conversion and selectivity were determined by GC using 1-butanol as a standard.

c 16 h.

d 30 h.

e Isolated yield is shown in parentheses.

More notably, high chemoselectivity in organic synthesis and drug discovery is highly desirable. Thus, we further examined the performance of Co@C–N(800) for the reductive amination of various benzaldehydes functionalized by steric groups (Table 4, entries 1–3), halogen (Table 4, entries 4–6), or other reduction-sensitive substituents (Table 4, entries 7–11). It was discovered that all the examined benzaldehydes could be efficiently and selectively transformed into the corresponding primary amines. Additionally, citronellal (a plant molecule), 5-hydroxymethylfurfural (a biomass platform molecule) and androsterone (a bioactive molecule) could also be transformed into the desired products with high yields of 94%, 71% and 90%, respectively (Table 4, entries 12–14). In particular, the reduction-sensitive substituents (*i.e.*, methoxy, ester, acylamino, C=C bonds, and furan rings) remained intact, confirming the unique advantage of Co@C–N(800) for reductive amination of substrates with reduction-sensitive substituents at such a mild reaction temperature (35 °C). The main reason was that the Co-based catalyst was not active for these reduction-sensitive groups.

**Table 4:** Reductive amination of various challenging substrates over Co@C–N(800)^a^

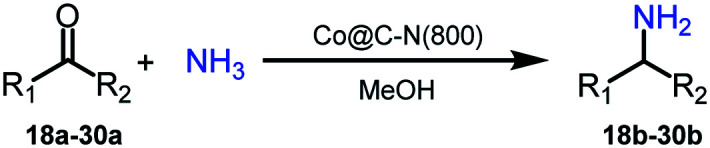			
Entry	Aldehydes	Products	Yields^b^^,^^f^ (%)
1	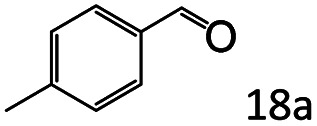	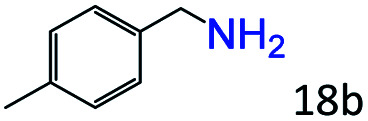	96 (92)
2	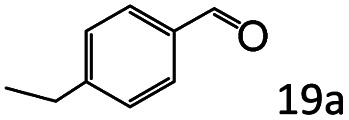	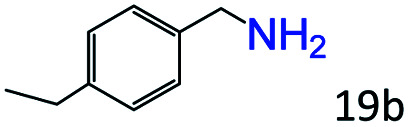	95 (91)
3	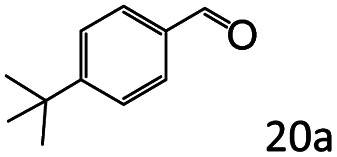	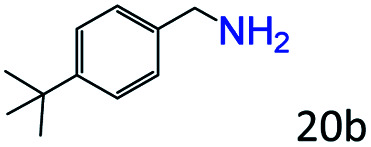	93 (90)
4^c^	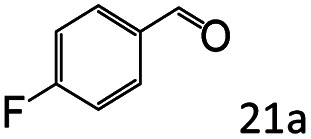	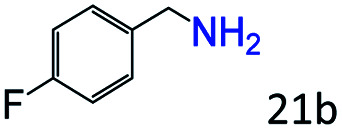	95 (92)
5^c^	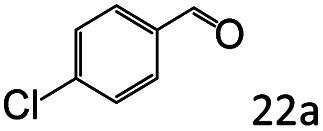	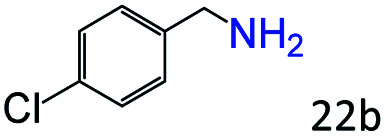	94 (90)
6^c^	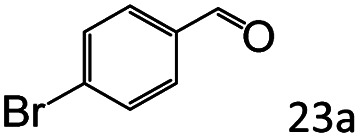	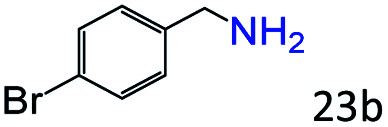	92 (87)
7^c^	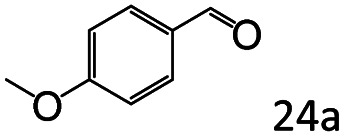	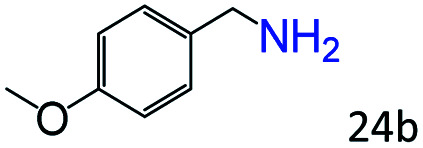	95 (91)
8	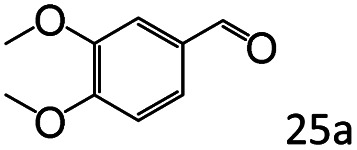	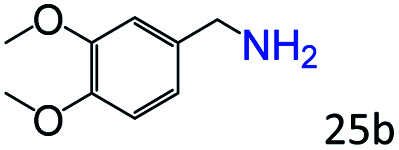	90 (87)
9	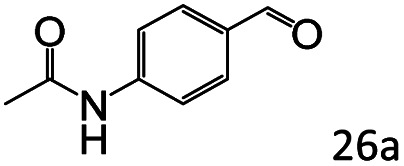	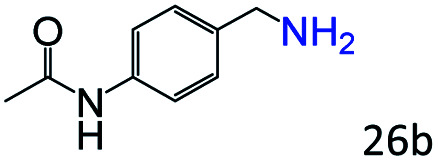	87 (85)
10	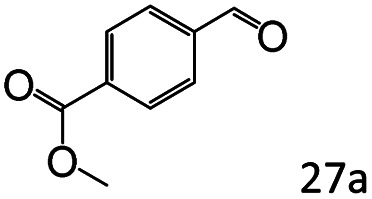	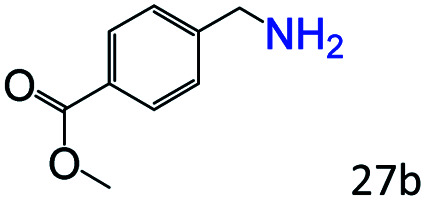	46
11^d^	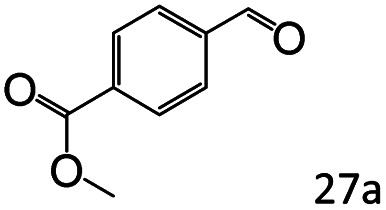	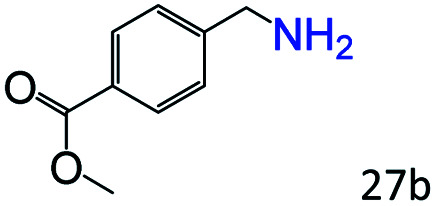	85 (80)
12^c^	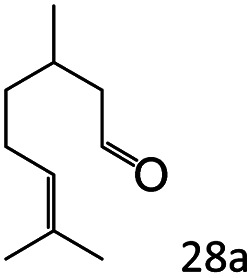	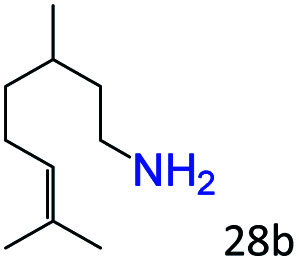	94 (92)
13^c^	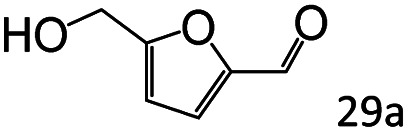	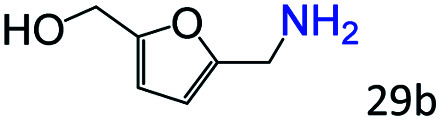	71 (65)
14^e^	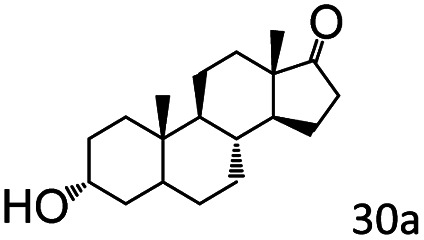	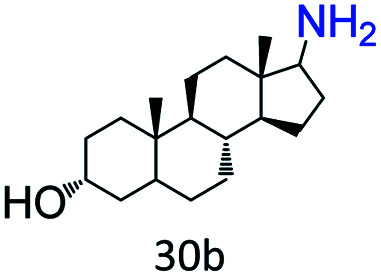	90 (89)

a Reaction conditions: ketone, 1 mmol; methanol, 3 mL; H_2_, 1.4 MPa; NH_3_, 0.6 MPa; Co@C–N(800), 50 mg (29.2 mol% Co); 35 °C; 30 h.

b The conversion and selectivity were determined by GC using 1-butanol as a standard.

c 24 h.

d 50 °C.

e 80 °C.

f Isolated yield is shown in parentheses.

### Mechanism investigation

**Fig. 4:**
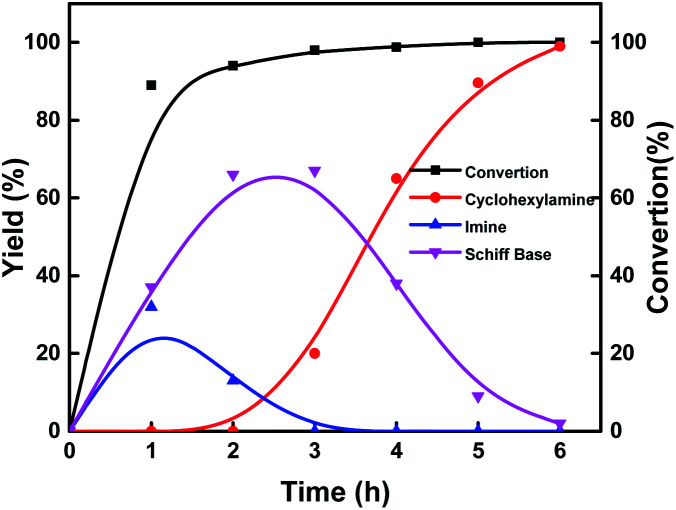
Time–yield plots. Reaction conditions: cyclohexanone, 1 mmol; methanol, 3 mL; 35 °C; Co@C–N(800), 50 mg (29.2 mol% Co); 1.4 MPa H_2_ and 0.6 MPa NH_3_.

**Fig. 5:**
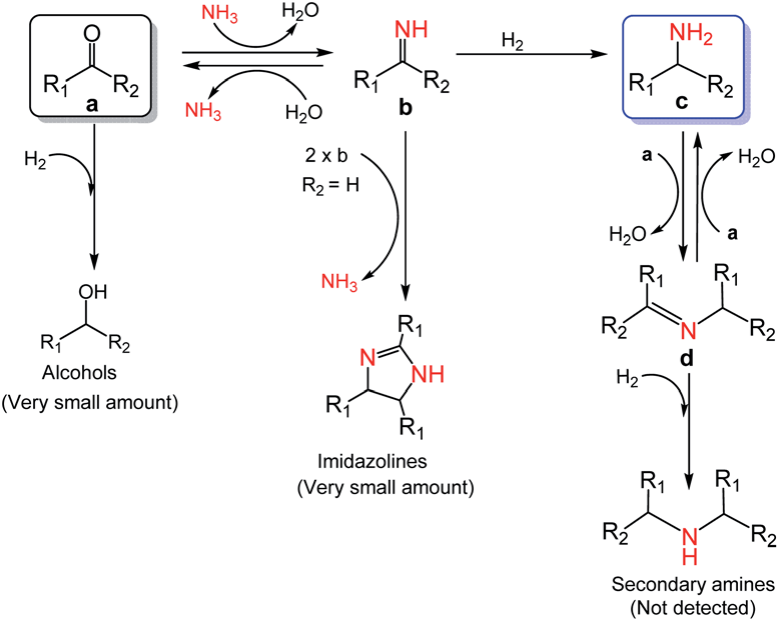
Proposed mechanism for the reductive amination of carbonyl compounds to primary amines using NH_3_ and H_2_ over Co@C–N(800).

Investigation of the reaction pathway is of significant importance to reveal the probable reaction mechanism. First, the dependence of product distribution on time was determined using reductive amination of cyclohexanone as the benchmark reaction (Fig. 5). As described in Fig. 5, cyclohexanone was rapidly converted in the initial 1.5 h with imine and Schiff base as the products. The Schiff base originated from the reaction of cyclohexanone with the cyclohexylamine formed by hydrogenation of imine. Because of the high reactivity between cyclohexanone and cyclohexylamine (Fig. S17a and b†), no desired cyclohexylamine was detected in this time period (initial 1.5 h). After most of the cyclohexanones were consumed, cyclohexylamine was observed when the reaction time was continually prolonged. Meanwhile, the amount of the Schiff base increased from 0 to 2.5 h and decreased after 2.5 h, and no secondary amine was detected in the reaction process, indicating that the Schiff base could be converted into the desired cyclohexylamine rather than being directly hydrogenated to form secondary amine. In addition, no cyclohexanol was generated in the whole catalytic process because of the rapid consumption of cyclohexanone with NH_3_ and the weak hydrogenation ability of Co@C–N(800) for direct hydrogenation of cyclohexanone at 35 °C (Fig. S17c†). These results implied that cyclohexylamine was generated through the direct hydrogenation of imine or the reaction pathway of forming a Schiff base, and the latter was the predominant pathway based on the product distribution (Fig. 4). Second, control experiments using the Schiff base as the reactant over Co@C–N(800) confirmed that cyclohexylamine was successfully synthesized in the presence of NH_3_ (Fig. S17d†), while secondary amine was formed without NH_3_ (Fig. S17e†). These results not only explained why no secondary amine was formed in the reaction process, but also further confirmed the important role of NH_3_ pressure, as discussed above (Fig. 3B). Third, we found that imidazolines at a very low yield would be generated from the *in situ* formed imines when aldehydes were applied as the substrates (Fig. S18†), indicating that imidazolines were the potential byproduct.

Based on the experimental results and some reported knowledge,^37,44^ a plausible mechanism was proposed for the reductive amination of carbonyl compounds using NH_3_ and H_2_ over Co@C–N(800) (Fig. 5). In the first step, imines could be rapidly formed by the reaction between the carbonyl group in the substrates and NH_3_, and this step could occur without using any catalysts. Subsequently, over Co@C–N(800), the imines were hydrogenated to generate the corresponding primary amines. When there were many unreacted carbonyl compounds, Schiff bases would be formed through the condensation of carbonyl compounds and the *in situ* formed primary amines. Finally, the formed Schiff bases were transformed into the target primary amines over Co@C–N(800) because NH_3_ was in excess in the reaction system. Additionally, only very small amounts of byproducts (*i.e.*, alcohols from direct hydrogenation of substrates, imidazolines from the reaction of two-molecule imines, and secondary amines from hydrogenation of Schiff bases) were detected in some cases, suggesting the high selectivity of this developed Co@C–N(800) catalytic system.

## Conclusions

In conclusion, robust Co-based catalysts were prepared by the pyrolysis of the precursor ZIF-67. The synthesized earth-abundant Co-based catalysts could efficiently catalyze reductive amination of aldehydes/ketones into primary amines with high activity and selectivity. The pyrolysis temperature had significant impact on the catalytic performance of the prepared Co-based catalysts, and the catalyst Co@C–N(800) showed the highest catalytic activity for the transformation. Various aldehydes/ketones, including those containing other reduction-sensitive substituents, could be selectively converted into the corresponding primary amines at a mild reaction temperature of 35 °C. Systematic study revealed that the excellent performance of Co@C–N(800) originated from the synergistic effect of metallic Co and the atomically dispersed Co–N_*x*_ sites. This work opens the way for efficient and selective synthesis of primary amines *via* reductive amination of carbonyl compounds over earth-abundant metal-based catalysts at mild temperature (35 °C). We believe that the prepared Co@C–N(800) has great application potential for the synthesis of primary amines from reductive amination of aldehydes/ketones, and pyrolysis of MOF materials provides an effective avenue to design robust earth-abundant metal-based catalysts. More importantly, through the suitable synergistic effect of different species, more robust earth-abundant metal-based catalysts could be hopefully designed to enable reductive amination of carbonyl compounds to be performed with lower metal usage and under ambient conditions, relying on developing innovative methods to construct this type of desired catalyst.

## Data availability

The authors declare that all data supporting the findings of this study are available within the paper and its ESI.†

## Author contributions

B. Zheng conceived the idea and performed the reaction tests. J. Xu helped to evaluate the experimental data. X. Mei and W. Wu helped to perform the SEM and XPS. K. Zhang and W. Han helped to perform the BET measurement (supporting). J. Song, H. Wu, and B. Han directed the project. B. Zheng, J. Song, H. Wu and B. Han co-wrote the manuscript. M. He helped to revise the manuscript. All the authors discussed the results and commented on the manuscript.

## Conflicts of interest

The authors declare no competing interests.

## Supplementary Material

SC-013-D2SC01596J-s001
