# Supplementary material for: Nanoparticles and single atoms of cobalt synergistically enabled low-temperature reductive amination of carbonyl compounds
Source: Chem Sci. 2022 Jul 8;13(31):9047–55. doi: 10.1039/d2sc01596j (PMC9365245; doi:10.1039/d2sc01596j)

Electronic Supplementary Material (ESI) for Chemical Science.
This journal is © The Royal Society of Chemistry 2022

Supporting Information for:

Nanoparticles and Single Atoms of Cobalt Synergistically Enabled Low-Temperature Reductive Amination of Carbonyl Compounds

Bingxiao Zheng,[a,c] Jiao Xu,[a,c] Jinliang Song, [b] Haihong Wu,* [a,c] Xuelei Mei,[a,c] Kaili Zhang,[a,c] Wanying Han,[a,c] Wei Wu,[a,c] Mingyuan He,[a,c] and Buxing Han* [a,c,d,e]*

[a] *Shanghai Key Laboratory of Green Chemistry and Chemical Processes, School of Chemistry and Molecular Engineering, East China Normal University, Shanghai 200062, China.*
E-mails: hhwu@chem.ecnu.edu.cn; hanbx@iccas.ac.cn

[b] *School of Chemical Engineering and Light Industry, Guangdong University of Technology, Guangzhou 510006, China.*
E-mail: songjl_2021@gdut.edu.cn

[c] *Institute of Eco-Chongming, Shanghai 202162, China.*

[d] *Beijing National Laboratory for Molecular Sciences, CAS Key Laboratory of Colloid, Interface and Chemical Thermodynamics, CAS Research/Education Center for Excellence in Molecular Sciences, Institute of Chemistry, Chinese Academy of Sciences, Beijing 100190, China.*

[e] *School of Chemistry and Chemical Engineering, University of Chinese Academy of Sciences, Beijing 100049, China.*

Table of Contents

1. Experimental Procedures..…………………………………..S3
2. References…………………………….………………..S17
3. Chromatograms of GC-measurements………………..….S18
4. Copies of ^{1}H NMR and ^{13}C NMR……………..………..…..S26

Experimental Procedures

Materials. 2-Methylimidazole (99%), cyclohexanone (99.8%),1-butanol (99.5%) and cobalt(II) nitrate hexahydrate (99%) were obtained from Acros. 3-Pentanone (98%), 4-methyl-2-pentanone (99%), cyclopentanone (99%), 2-nonanone (99%), 4-ethylbenzaldehyde (98%), acetophenone (99%), 4-*tert*-butylbenzaldehyde (97%) and benzaldehyde (99%) were purchased from Aladdin. Butyraldehyde (99%), cycloheptanone (99%), hexanal (99%), 2-octanone (99%), *n*-octanal (99%) and *p*-anisaldehyde (99%) were provided by TCI. 3-Phenylpropanal (99%) was purchased from ARK. 4-Phenyl-2-butanone (98%), *p*-methyl benzaldehyde (98%), 3-pyridinecarboxaldehyde (98%), 4-fluorobenzaldehyde (98%), 4-chlorobenzaldehyde (98%), *p*-bromo benzaldehyde (99%), veratraldehyde (98%), (±)-citronellal (96%), methyl *p*-formylbenzoate (98%), 4-acetamidobenzaldehyde (98%), furfural (99%), 5-hydroxymethyl-2-furaldehyde (98%), androsterone (98%) and 2-adamantanone (98%) were obtained from Innochem. Methanol (99.5%) and hydrochloric acid (36.0 wt%) was obtained from Sinopharm.

Synthesis of ZIF-67. In a typical procedure, $Co(NO_3)_2{\cdot}6H_2O$ (0.45 g) was dissolved in 3 mL water to form a clear solution, which was subsequently injected into 40 mL of methanol containing 5.5 g 2-methylimidazole (MeIM) under ultrasound for 20 min at room temperature. The mixed solution was then transferred into 50 mL Teflon-lined stainless-steel autoclaves and heated at 120 ^{o}C for 4 h. The as-obtained precipitations were centrifuged and washed with ethanol several times and dried in vacuum at 60 ^{o}C for overnight.

General procedures to conduct the reaction. In a typical route, the desired carbonyl compound and catalyst were charged into a stainless-steel reactor with a Teflon coating (15 mL inner volume). After the reactor was sealed, it was purged with 0.5 MPa NH_3 three times to remove residual air. After that, the NH_3 was charged into the reactor to reach a desired pressure. Then, the reactor was further pressurized with H_2. Then, the autoclave was placed into a constant-temperature air bath and heated to the desired temperature, and the reactions were conducted with a stirring rate of 800 rpm for the desired reaction time. After the reaction, the products were analyzed quantitatively using *n*-butanol as the internal standard (cyclohexanol for the case with butyraldehyde as the substrate) by gas chromatography (GC) and gas chromatography coupled with mass spectroscopy (GC-MS). And the obtained solution was then separated using the flash chromatography system with an amino column (eluent, MeOH and

CH_3Cl or AcOEt and hexane). The filtrate was evaporated to obtain the analytically pure products.

Synthesis of Co@C-N(x). The ZIF-67 was transferred to a furnace and pyrolyzed for 3 h at 800 ^oC (or 600, 700, 900) for 3 h at the heating rate of 5 ^oC/min under flowing nitrogen and then naturally cooled to room temperature to obtain the Co@C-N(800).

Recycling of catalyst. The recyclability of the Co@C-N(800) catalyst was tested for reductive amination at the same reaction conditions as described above, except using the recovered catalyst. For each cycle, the catalyst was isolated from the reaction mixture by magnetic separation at the end of catalytic reaction, thoroughly washed with methanol, and then treated in H_2/Ar (10%) at 400 ^oC for 1 h.

Synthesis of Co@C-N(800)-H$^+$ and Co@C-N(800)-air. The prepared Co@C-N(800) was dispersed in HCl aqueous solution (36.0 wt%), and stirred at room temperature for 48 h. Then, the materials were filtered off, washed with water and dried at 80 ^oC for 12 h. The obtained material was denoted as Co@C-N(800)-H$^+$. For the preparation of Co@C-N(800)-air, the Co@C-N(800) was heated at 400 ^oC for 1 h in air. Then, the obtained powder was reduced by heating at 400 ^oC for 1 h in H_2/Ar (10%) to give the Co@C-N(800)-air material. The Co content of the treated catalysts are also provided in Table S2.

Synthesis of Co/C. In this process, 0.3 g of activated carbon was dispersed in 20 mL of water. Then, cobalt nitrate hexahydrate (0.8 g) was added into the above dispersion. The mixture was stirred at room temperature for 12 h. Then, it was evaporated to remove the solvent. The obtained product was transferred to a furnace and pyrolyzed for 3 h at 800 ^oC for 3 h at the heating rate of 5 ^oC/min under flowing nitrogen and then naturally cooled to room temperature to obtain the Co/C.

Synthesis of the standard cyclohexylimine. Because there was no commercial resource, cyclohexylamine was pre-synthesized as the standard product to determine the yield of cyclohexylamine generated in the catalytic cycle. The cyclohexylimine was pre-synthesized based on a previously reported route.[1] Initially, 3M ammonia water in methanol (25 mL) was added into a flask of 25 mL. Subsequently, cyclohexanone (4 mmol) was added dropwise with a time increment of 5 minutes at room temperature. After the cyclohexanone was completely added, the reaction mixture was continually stirred for 24 h at room temperature. Finally, the

precipitate in the system was collected by filtration, washed by hexane, and dried at room temperature to obtain the desired cyclohexylimine.

Instruments. Conversions and yields were determined by a gas chromatography (Agilent 8890B) with the FID detector and a HP-5 column (30 m x 320 μm x 0.25μm). Tube furnace (Anhui Kemi Instrument Co., Ltd., CHEM^{N}, TFH-1200-440). Thermalfisher Scientific Titan Themsis Z. (HAADF-STEM). ^{1}H, ^{13}C NMR data were recorded on a Bruker ARX 400 and Bruker ARX 500 spectrometers using DMSO-d6 or CD_3OD solvents.

Catalyst Characterization. Inductively coupled plasma atomic emission spectrometry (ICP-AES) was adopted to measure the actual Co content. X-ray diffraction (XRD) patterns were obtained using Rigaku and model with Cu Kα radiation (1.5418 Å). X-ray photoelectron spectroscopy (XPS) study was conducted on the AXIS Supra surface analysis instrument with an X-ray monochromatic source (combined Al/Ag anode, energy 1486.6/2984.2eV) and operated on 10^{-9} mbar. The morphology of the catalysts was observed on Hitachi S4800 scanning electron microscope (SEM) at 3 kV and transmission electron microscopy (TEM) (Tecnai G2 F30 FETEM). Nitrogen adsorption−desorption analysis at 77 K was carried out on a Quantachrome Autosorb-3B system after the catalyst was outgassed under vacuum at 100 ^{o}C for 12 h. The specific surface area was determined according to the Brunauer–Emmett–Teller (BET) equation from the adsorption branch. The X-ray absorption spectroscopy (XAS) experiments were carried out at the 4B9A beamline at Beijing Synchrotron Radiation Facility (BSRF), China. Extended X-ray absorption fine structure (EXAFS) spectra were conducted using the Athena software package.

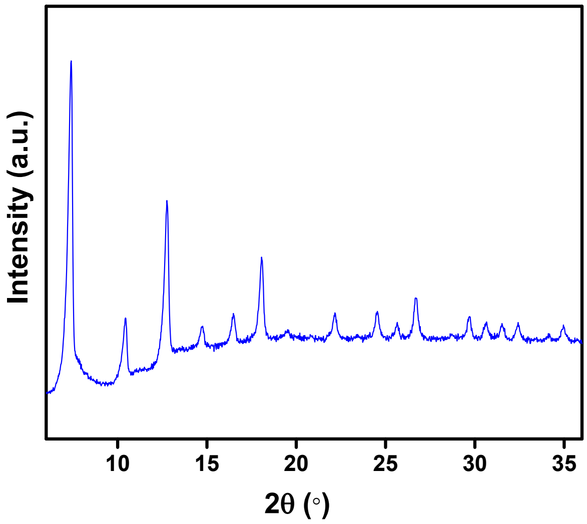

Fig. S1. Powder XRD patterns of ZIF-67.

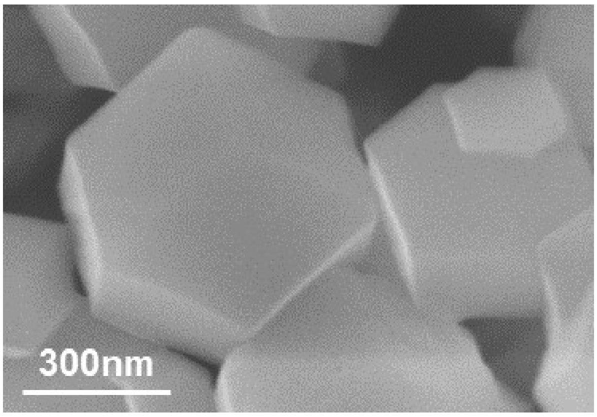

Fig. S2. SEM images of ZIF-67.

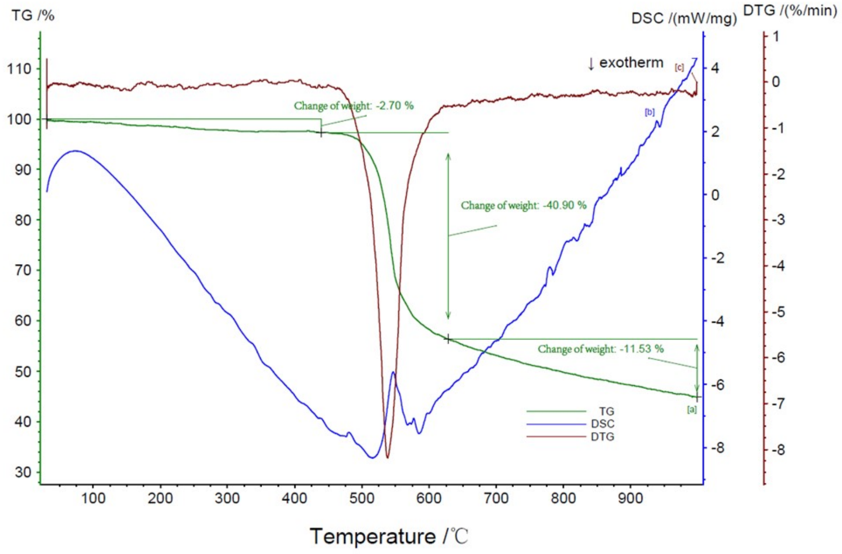

Fig. S3. Thermal analysis of ZIF-67: (a) TGA, (b) DSC, and (c) DTG.

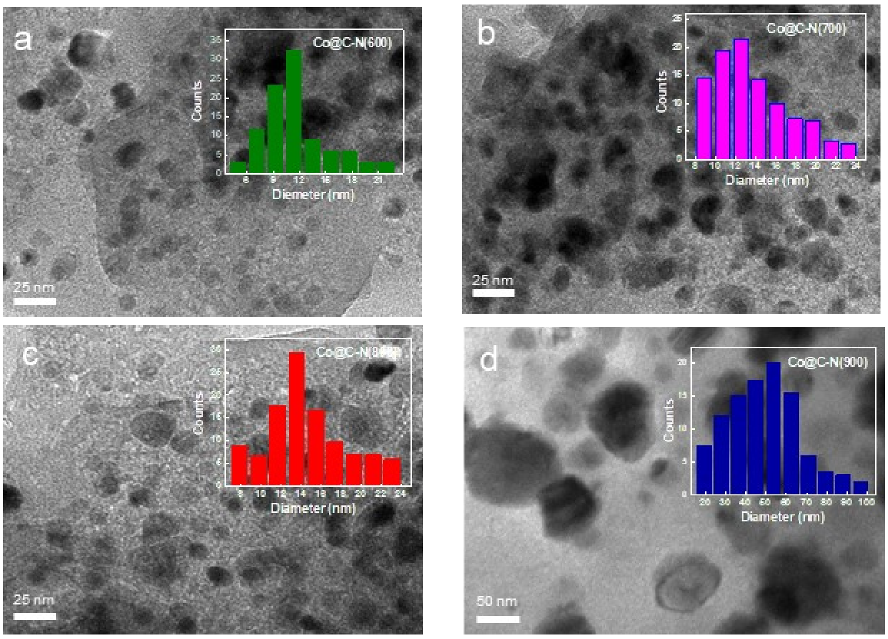

Fig. S4. TEM images and corresponding size distribution of (a) Co@C-N(600), (b) Co@C-N(700), (c) Co@C-N(800) and (d) Co@C-N(900).

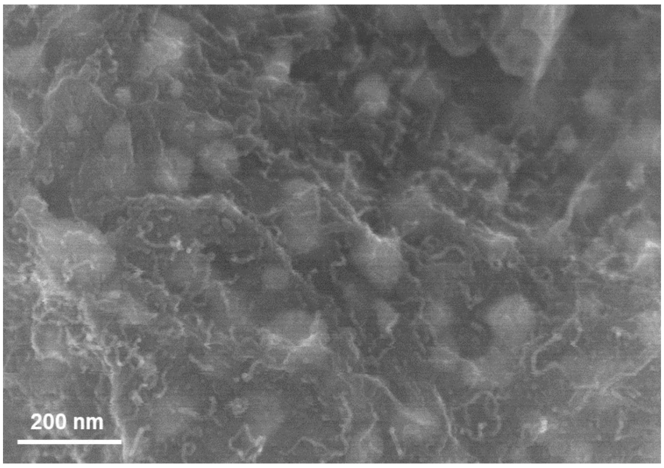

Fig. S5. SEM images of Co@C-N(900).

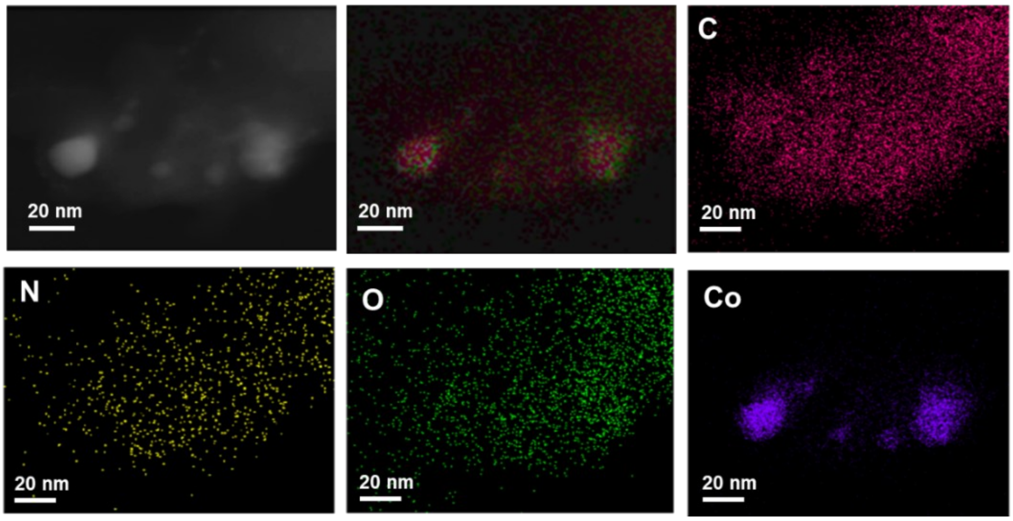

Fig. S6. TEM and EDS mapping images of the prepared Co@C-N(800).

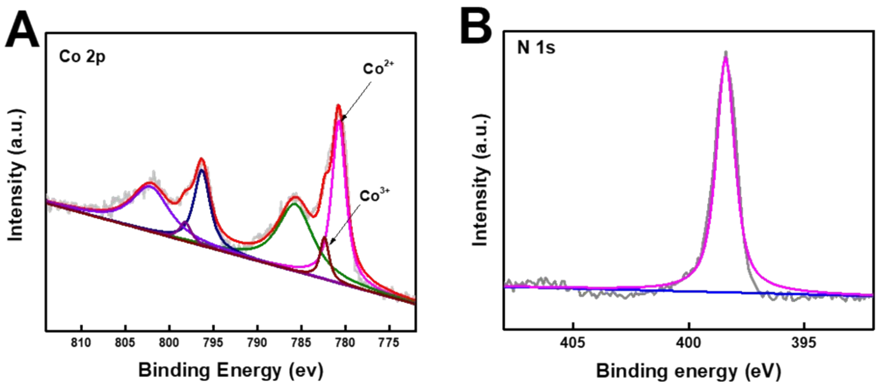

Fig. S7. (A) Co 2p spectra and (B) N 1s spectra of ZIF-67.

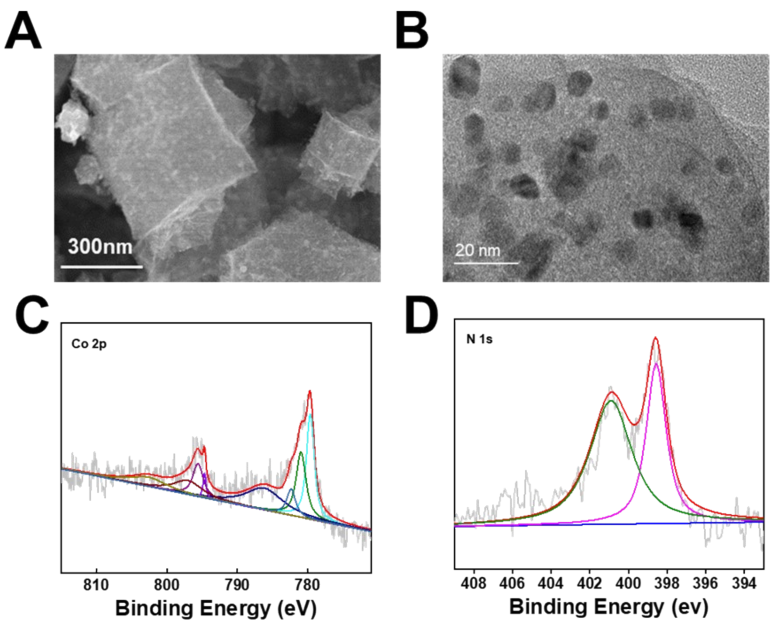

Fig. S8. SEM image, TEM image, XPS spectra of Co 2p and N 1s for the recovered Co@C-N(800).

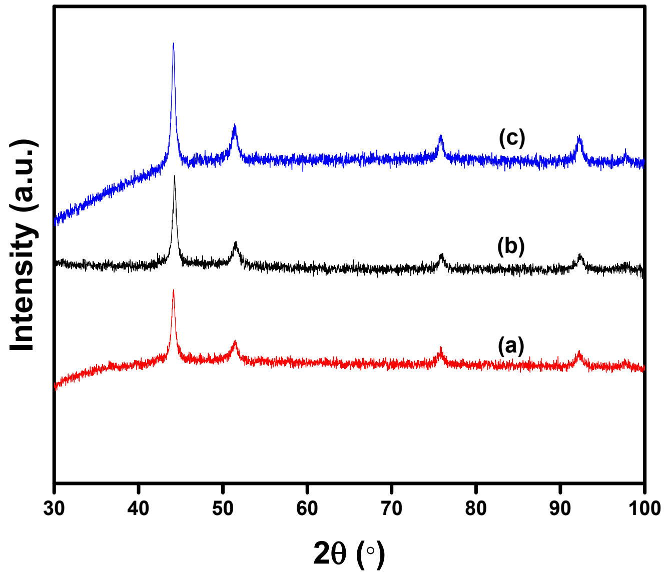

Fig. S9. Powder XRD patterns for (a) fresh Co@C-N(800), (b) recycled Co@C-N(800) without H_2 treatment, (c) recycled Co@C-N(800) after H_2/Ar (10%) treatment.

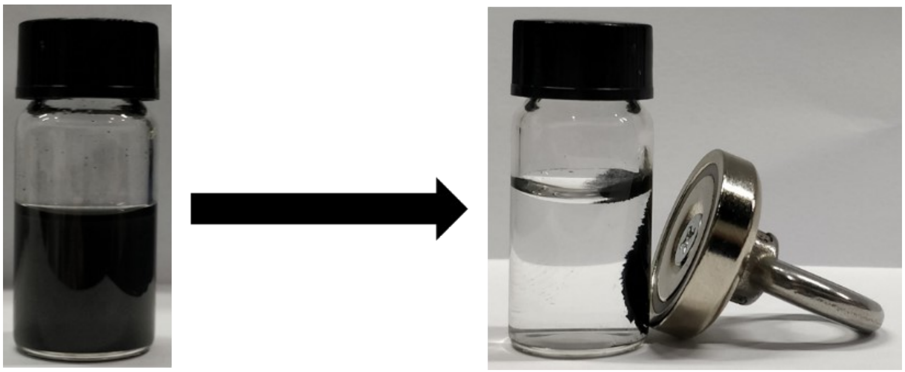

Fig. S10. Magnetic separation of the catalyst after reaction.

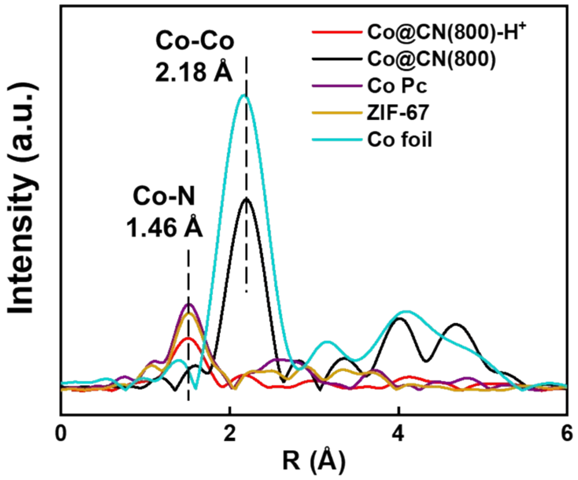

Fig. S11. the corresponding Fourier transforms EXAFS spectra of Co@C-N(800)-H^+, Co@C-N(800)-air, Co@C-N(800), Co Pc, ZIF-67 and Co foil.

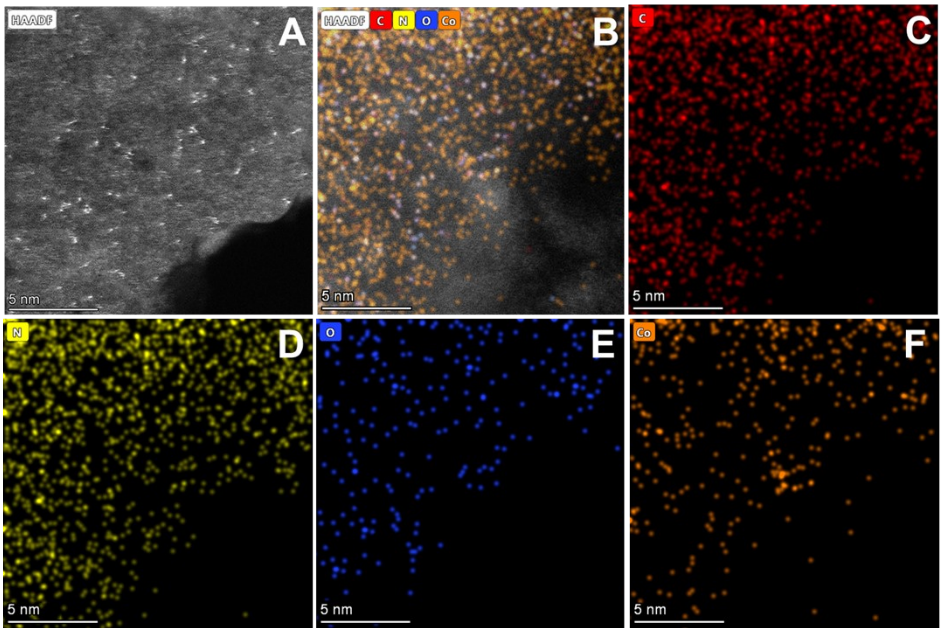

Fig. S12. (A) HAADF-STEM image of Co@C-N(800)-H^+, in which single Co atoms were clearly seen, and (B-F) EDS mappings of the Co@C-N(800)-H^+.

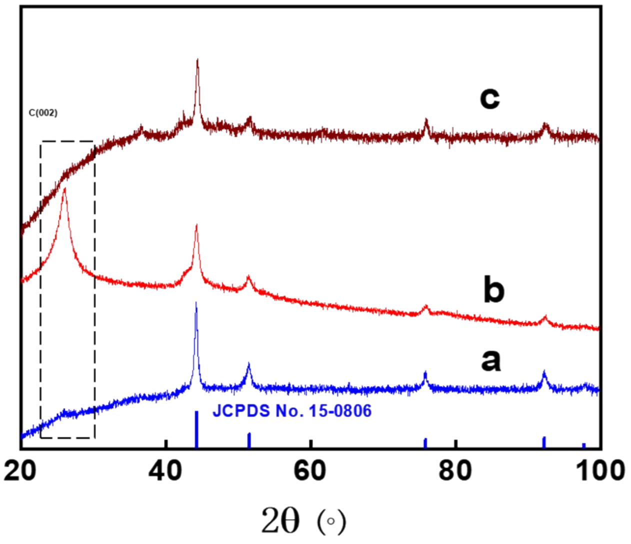

Fig. S13. Powder XRD patterns for (A) fresh Co@C-N(800), (B) Co@C-N(800)-H^+, (C) Co@C-N(800)-air.

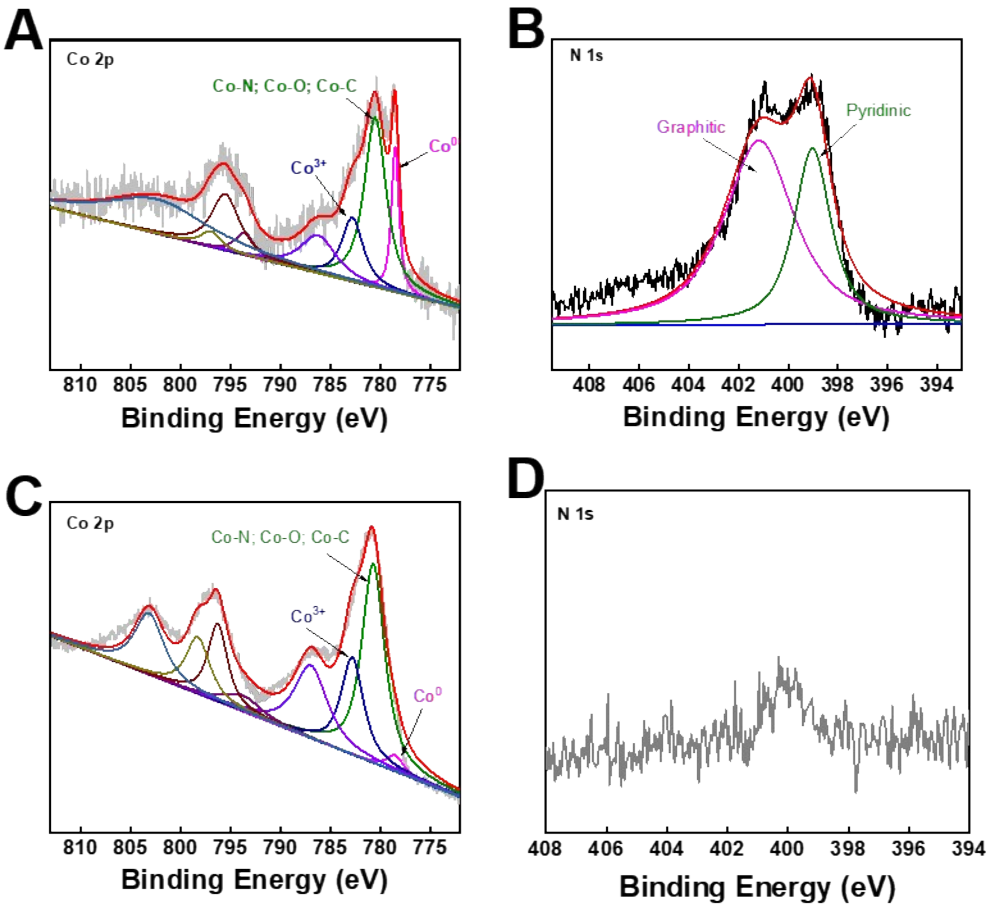

Figure S14. A) Co 2p spectra and B) N 1s spectra of Co@C-N(800)-H^+ ; C) Co 2p spectra and D) N 1s spectra of Co@C-N(800)-air.

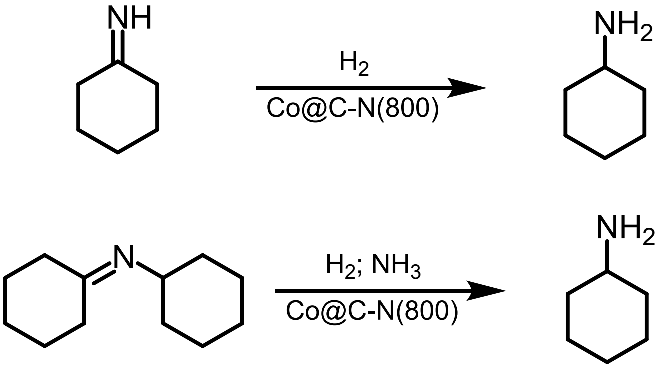

Fig. S15. The hydrogenation steps in the reaction process.

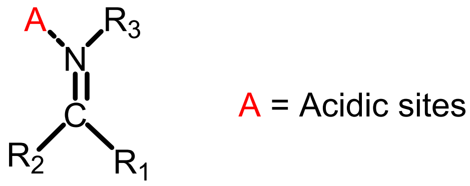

Fig. S16. The activation of C=N groups in *in situ* generated imines and Schiff bases by the acidic sites on Co@C-N(x).

Table S1. Summary of the results from N_2 adsorption-desorption

Entry	Catalyst	S_{BET} (m^2 g^{-1})	Pore volume (cm^3 g^{-1})	Pore size (nm)
1	ZIF-67	1352	0.64	3.30
2	Co@C-N(600)	225	0.18	2.90
3	Co@C-N(700)	202	0.19	2.96
4	Co@C-N(800)	180	0.18	2.98
5	Co@C-N(900)	105	0.20	2.97

Table S2. ICP-OES analysis of different catalysts.

Entry	Catalyst	Co content (wt%)
1	ZIF-67	23.8
2	Co@C-N(600)	27.2
3	Co@C-N(700)	33.7
4	Co@C-N(800)	34.5
5	Co@C-N(900)	37.3
6	Co@C-N(800) (1 cycle)	32.5
7	Reaction solution	0
8	Co@C-N(800)-H^+	0.9
9	Co@C-N(800)-air	95.0

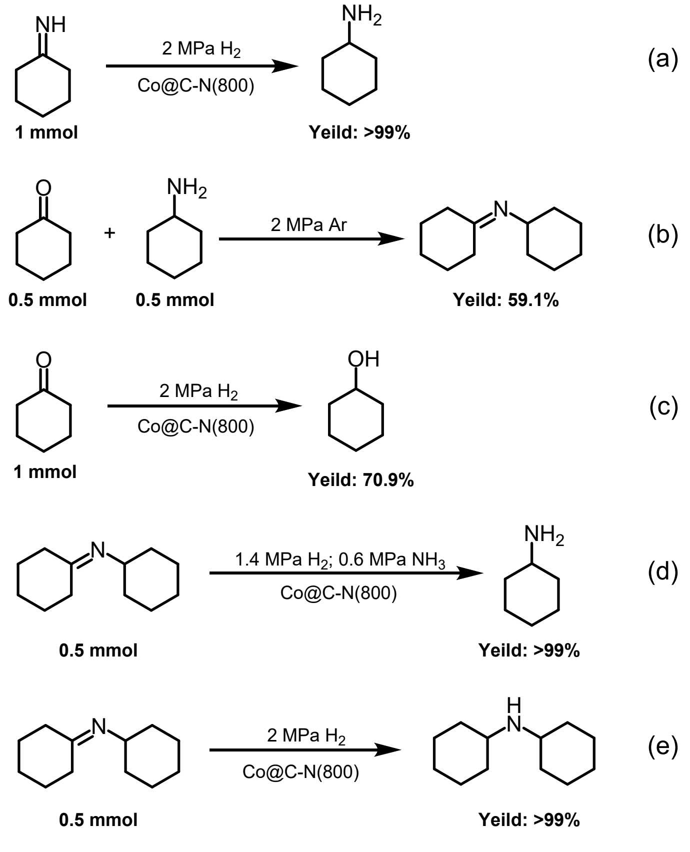

Fig. S17. Control experiments using diffferent reactant over Co@C-N(800). Reaction conditions: methanol, 3 mL; Co@C-N(800), 50 mg; 35 ^{o}C; 6 h.

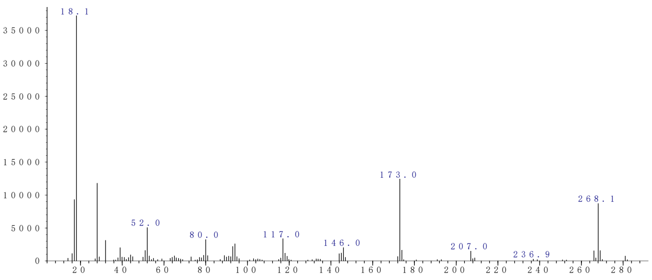

Fig. S18. GC-MS spectra of imidazoline derived from the reductive amination of furfural catalyzed by Co@C-N(800).

Table S3. Comparison of Co@C-N(800) with state-of-the-art catalysts in reductive amination of aldehydes and ketones into primary amines.

Catalyst	Ammonia and hydrogen source	T (℃)	Time (h)	mol% metal based on substrate	TON	TOF (h^{-1})	Product	Yield(%)	Ref.
Ru/ZrO_2	3 mL 25 wt% NH_3, 1.2 MPa H_2	95	8	2.5	40.5	5.1	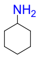	99	2
Ru/ZrO_2	3 mL 25 wt% NH_3, 1.2 MPa H_2	95	6	2.5	36.5	6.1	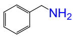	90	2
Ru/Nb_2O_5	0.1 MPa NH_3, 4 MPa H_2	90	1	0.24	400	400	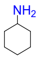	96	3
Ru/Nb_2O_5	0.1 MPa NH_3, 4 MPa H_2	90	6	0.24	408.3	68.1	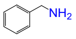	98	3
$RuCl_2(PPh_3)_3$	5-7 bar NH_3, 4 MPa H_2	130	30	3	28.7	1.0	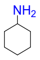	86	4
$RuCl_2(PPh3)_3$	5-7 bar NH_3, 4 MPa H_2	130	24	3	31.7	1.3	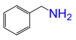	95	4
Co-DABCO-TPA@C-800	5-7 bar NH_3, 4 MPa H_2	120	15	3.5	26.3	1.8	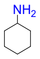	92	5
Co-DABCO-TPA@C-800	5-7 bar NH_3, 4 MPa H_2	120	15	3.5	24.9	1.7	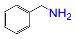	87	5
Ni/Al_2O_3	0.5 mL 25 wt% NH_3, 1 MPa H_2	80	20	1.4	71.0	3.6	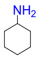	99	6
Ni/Al_2O_3	0.5 mL 25 wt% NH_3, 1 MPa H_2	80	20	1.4	71.0	3.6	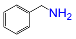	99	6
Ru-NPS	8 mmol NH_3, 2 MPa H_2	90	1	0.04	2425	2425	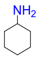	97	7
Ru-NPS	8 mmol NH_3, 2 MPa H_2	90	4	0.04	2375	594.0	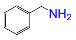	95	7
Co@CN-800	2 mL 26.5 wt% NH_3, 2 MPa H_2	130	12	13	7.35	0.6	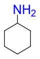	95.5	8
Co@CN-800	2 mL 26.5 wt% NH_3, 0.5 MPa H_2	110	4	13	7.1	1.8	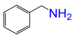	92	8
Ru/TiP-100	0.6 MPa NH_3, 1.4 MPa H_2	30	5	0.2	485	97.0	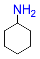	97	9
Ru/TiP-100	0.3 MPa NH_3, 1.7 MPa H_2	30	15	0.2	480	32.0	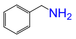	96	9
Pd-NPs	0.2 mmol NH_3, 0.1 MPa H_2	30	3	9.8	3.9	1.3	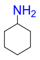	38	10
Pd-NPs	0.2 mmol NH_3, 0.1 MPa H_2	30	3	9.8	10.0	3.3	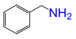	98	10
Ni-NPs	0.3 MPa NH_3, 0.9 MPa H_2	50	48	18	5.3	0.1	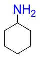	95	11
Ni-NPs	0.3 MPa NH_3, 0.9 MPa H_2	50	48	18	4.7	0.1	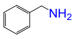	84	11
Co-NPs	3.5 mL 26.5 wt% NH_3, 1 MPa H_2	50	20	1.5	20.3	1.0	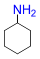	61	12
Co-NPs	3.5 mL 26.5 wt% NH_3, 1 MPa H_2	50	20	1.1	---	---	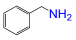	Not detected	12

Co-NPs	3.5 mL 26.5 wt% NH_3, 1 MPa H_2	80	20	1.1	41.8	2.1	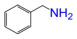	92	12
Raney®Ni	3000 mmol NH_3, 9 MPa H_2	70	0.5	---	---	---	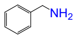	89	**13**
Co@CN-800	0.6 MPa NH_3, 1.4 MPa H_2	35	6	29.2	3.4	0.6	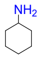	99	**This work**
Co@CN-800	0.3 MPa NH_3, 1.7 MPa H_2	35	16	29.2	3.4	0.2	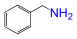	98	**This work**
Co@CN-800	0.6 MPa NH_3, 1.4 MPa H_2	35	24	5.8	16.4	0.7	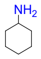	95	**This work**
Co@CN-800	0.6 MPa NH_3, 1.4 MPa H_2	35	24	8.0	12.5	0.5	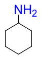	99	**This work**
Co@CN-800	0.6 MPa NH_3, 1.4 MPa H_2	50	12	5.8	17.2	1.4	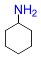	99	**This work**

References

1. X. Jv, S. Sun, Q. Zhang, M. Du, L. Wang, and B. Wang, *ACS Sustainable Chem. Eng.*, 2020, **8**, 1618−1626.
2. G. Liang, A. Wang, L. Li, G. Xu, N. Yan and T. Zhang, *Angew. Chem. Int. Ed.*, 2017, **56**, 3050-3054.
3. T. Komanoya, T. Kinemura, Y. Kita, K. Kamata and M. Hara, *J. Am. Chem. Soc.*, 2017, **139**, 11493-11499.
4. T. Senthamarai, K. Murugesan, J. Schneidewind, N. V. Kalevaru, W. Baumann, H. Neumann, P. C. J. Kamer, M. Beller and R. V. Jagadeesh, *Nat. Commun.*, 2018, **9**, 4123.
5. R. V. Jagadeesh, K. Murugesan, A. S. Alshammari, H. Neumann, M.-M. Pohl, J. Radnik and M. Beller, *Science*, 2017, **358**, 326-332.
6. G. Hahn, P. Kunnas, N. de Jonge and R. Kempe, *Nat. Catal.* **2019**, *2*, 71-77.
7. D. Chandra, Y. Inoue, M. Sasase, M. Kitano, A. Bhaumik, K. Kamata, H. Hosono and M. Hara, *Chem. Sci.*, 2018, **9**, 5949-5956.
8. X. Liu, Y. Wang, S. Jin, X. Li and Z. Zhang, *Arab. J. Chem.*, 2020, **13**, 4916-4925.
9. C.Xie, J. Song, M. Hua, Y. Hu, X. Huang, H. Wu, G.Yang and B. Han, *ACS Catal.*, 2020, **10**, 7763−7772.
10. X. Jv, S. Sun, Q. Zhang, M. Du, L. Wang and B. Wang, *ACS Sustainable Chem. Eng.*, 2020, **8**, 1618-1626.
11. M. K. Bhunia, D. Chandra, H. Abe, Y. Niwa and M. Hara, *ACS Appl. Mater. Interfaces*, 2022, **14**, 4144−4154.
12. M. Elfinger, T. Schönauer, S. L. J. Thomä, R. Stäglich, M. Drechsler, M. Zobel, J. Senker and R. Kempe, *ChemSusChem*, 2021, **14**, 2360–2366.
13. C. F. Winans, *J. Am. Chem. Soc.*, 1939, **61**, 3566 –3567.

Chromatograms for GC-measurements

Cyclopentylamine

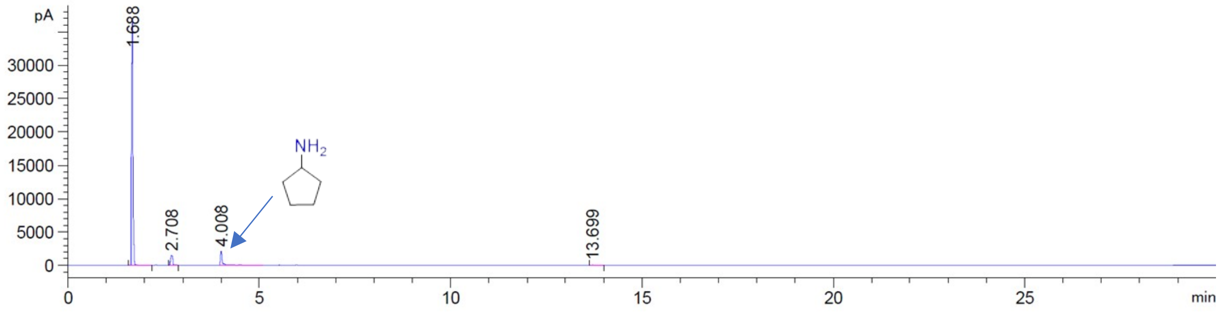

Cyclohexanamine

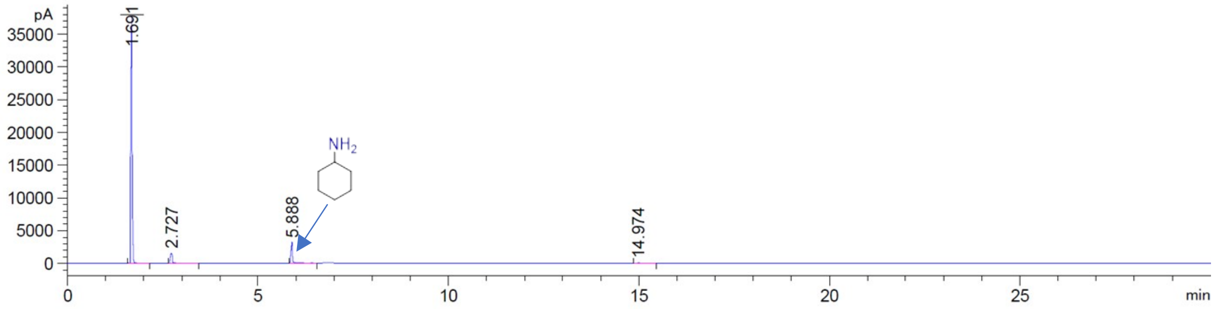

Cycloheptanamine

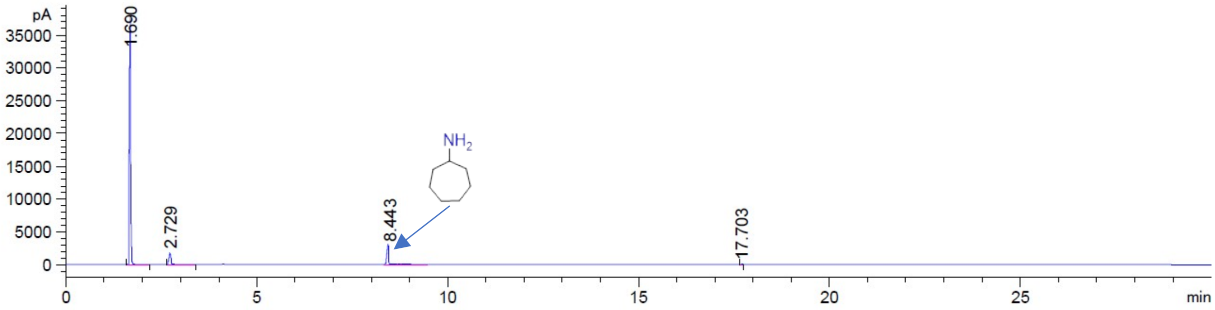

4-Methylpentan-2-amine

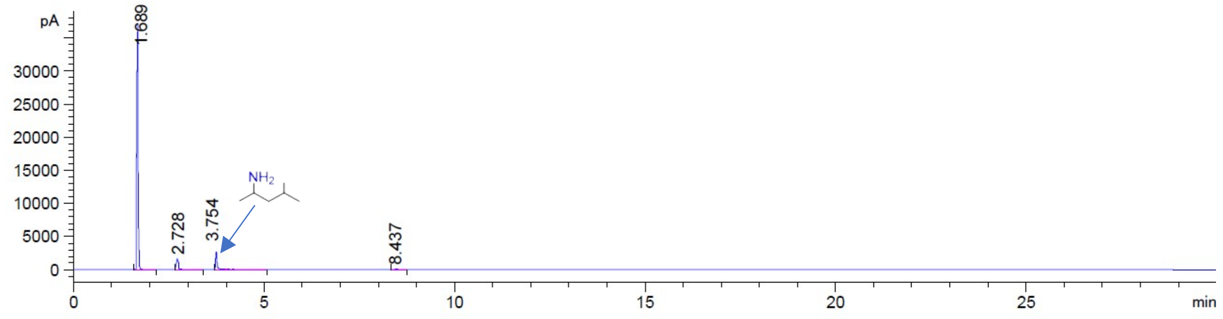

Pentan-3-amine

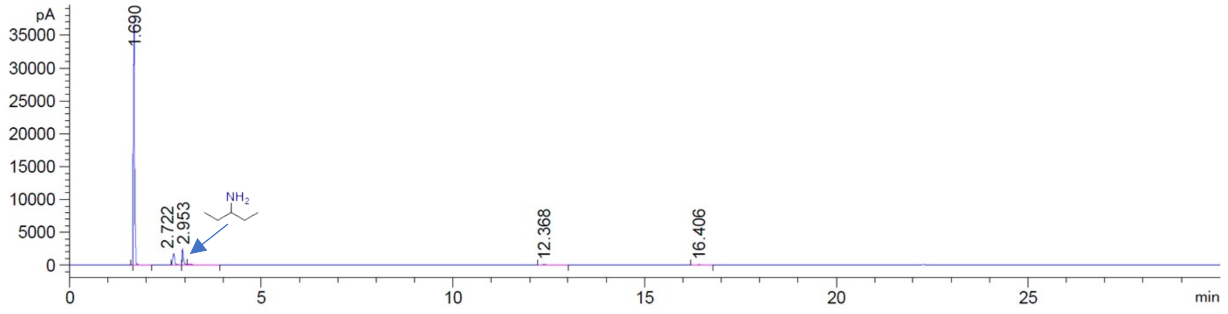

Octan-2-amine

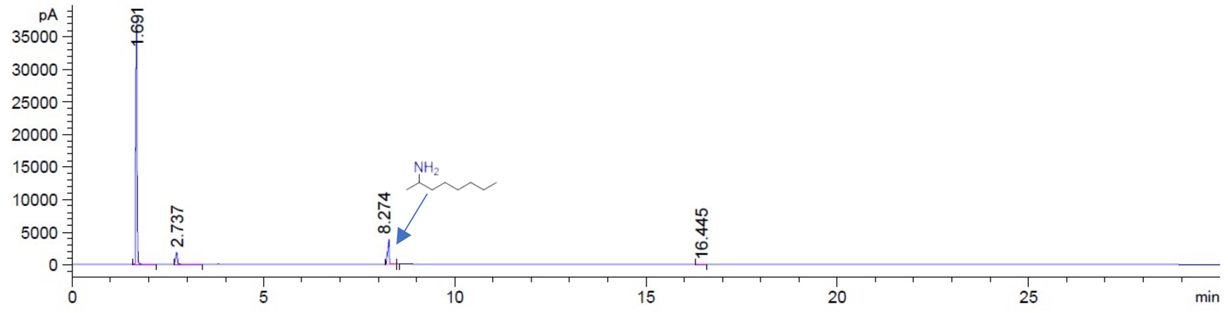

Nonan-2-amine

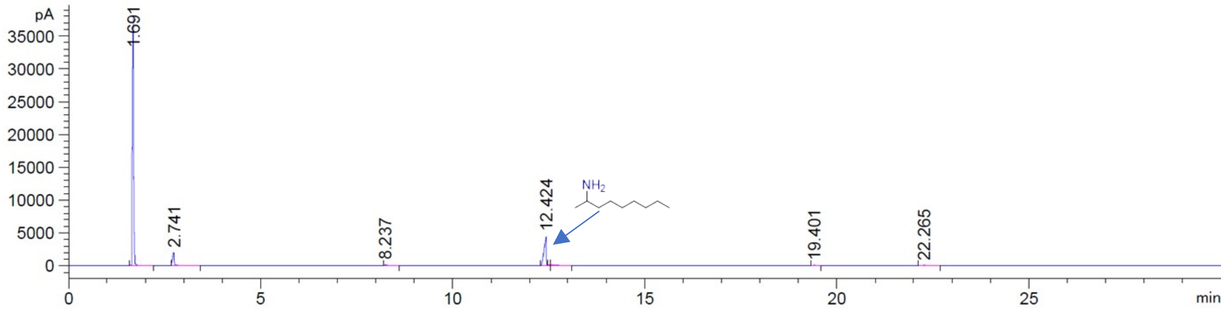

4-Phenylbutan-2-amine

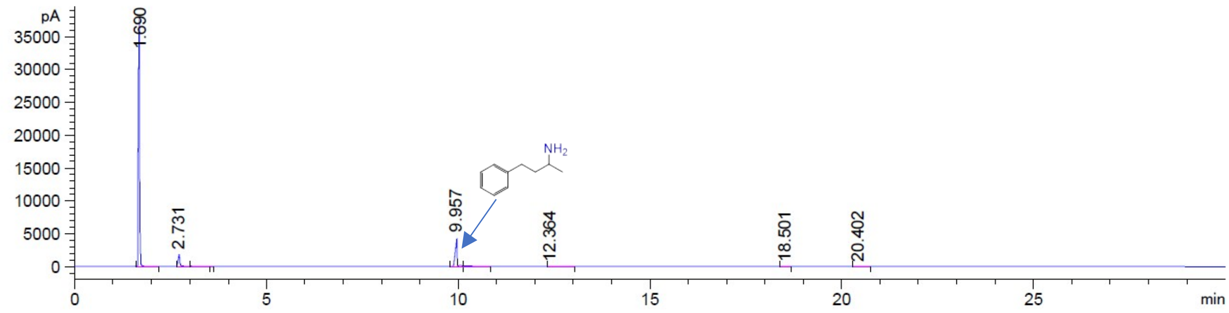

1-Phenylethan-1-amine

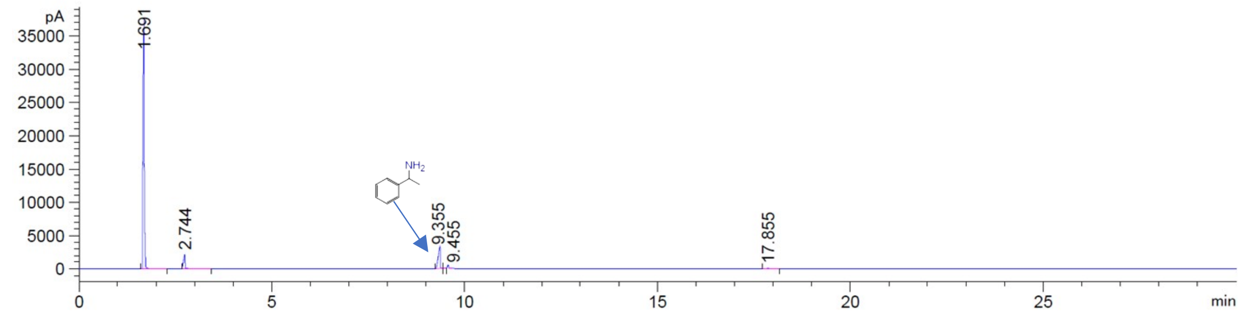

1-Adamantanamine

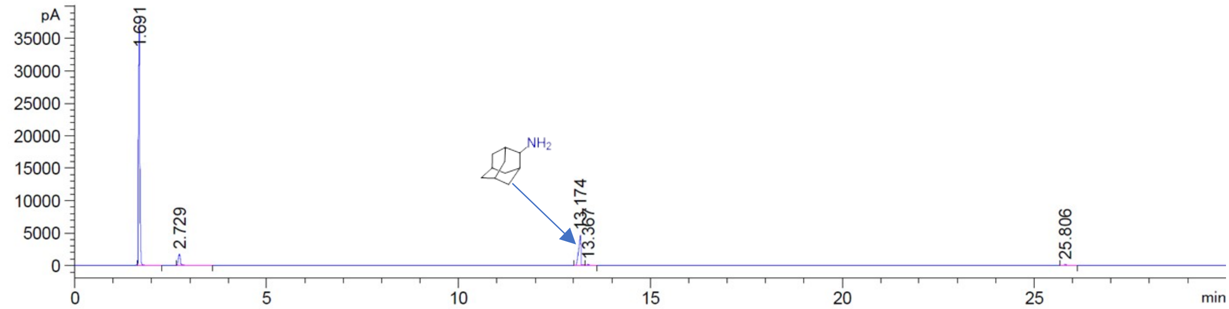

n-Butylamine

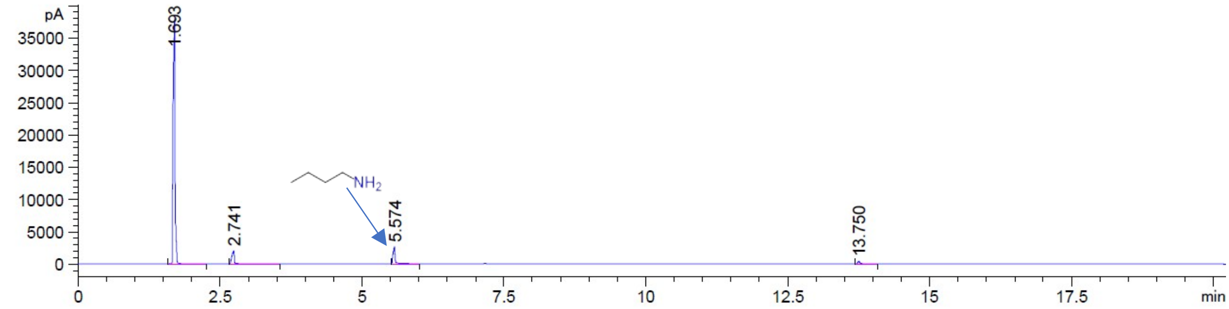

n-hexylamine

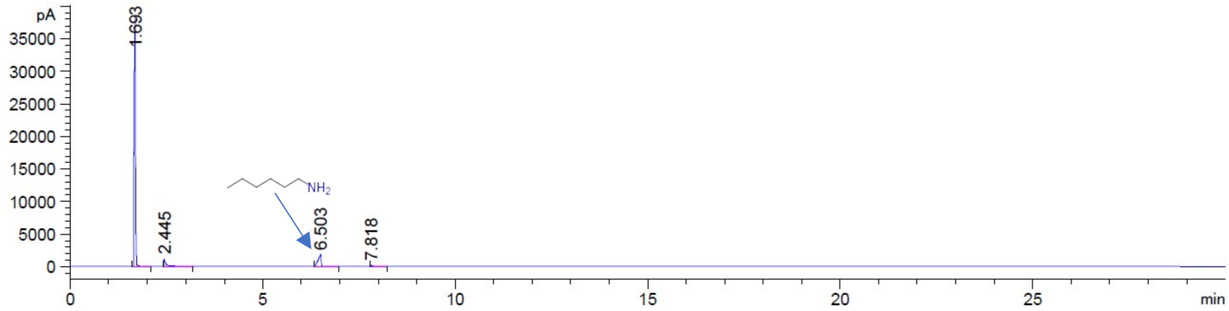

Octan-1-amine

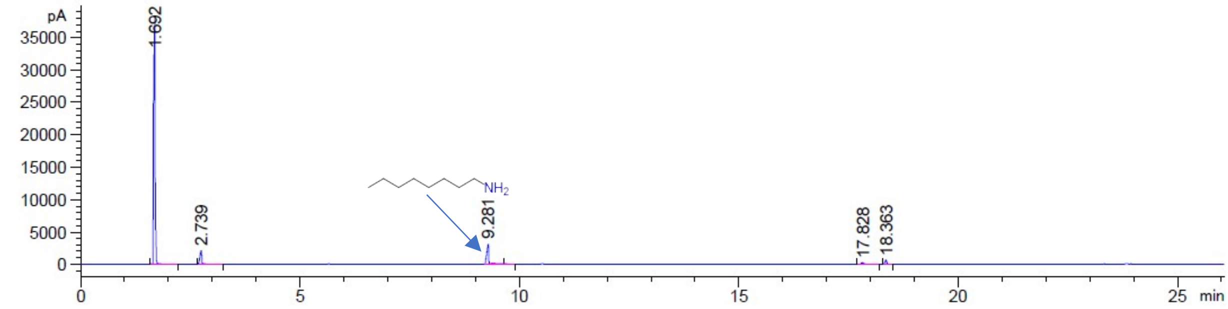

3-Phenylpropan-1-amine

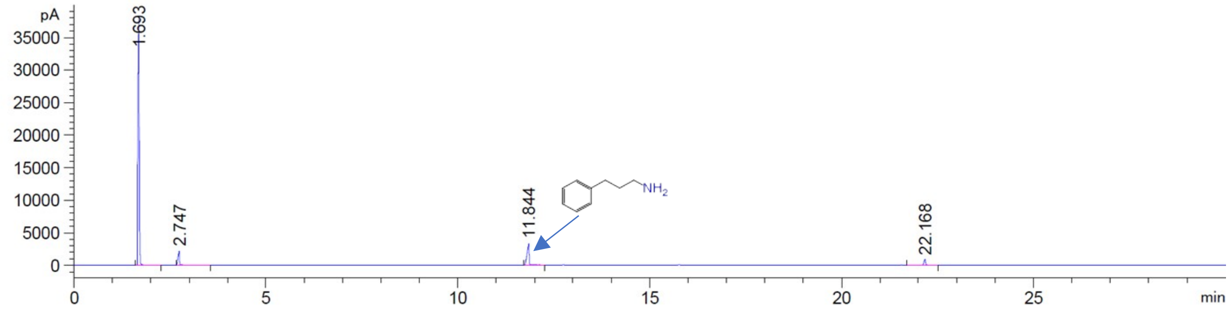

Phenylmethanamine

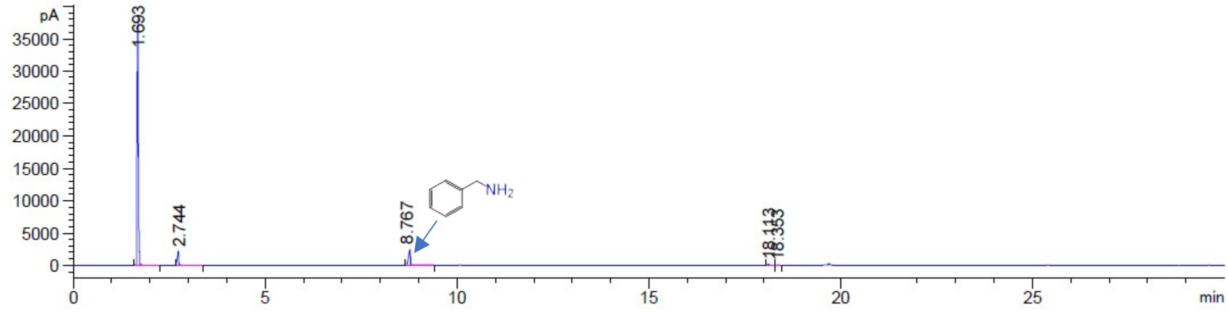

Pyridin-3-ylmethanamine

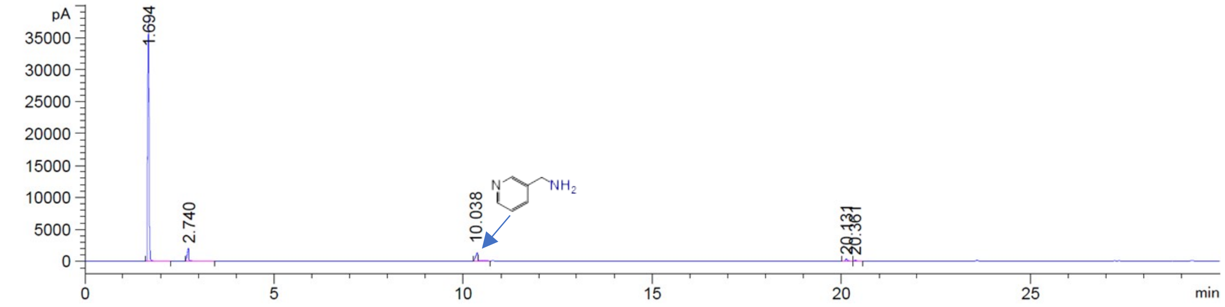

Furan-2-ylmethanamine

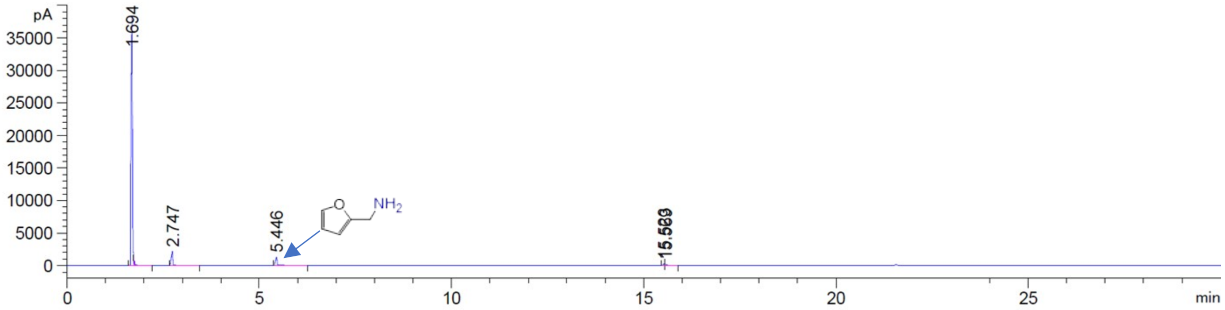

4-Methylbenzylamine

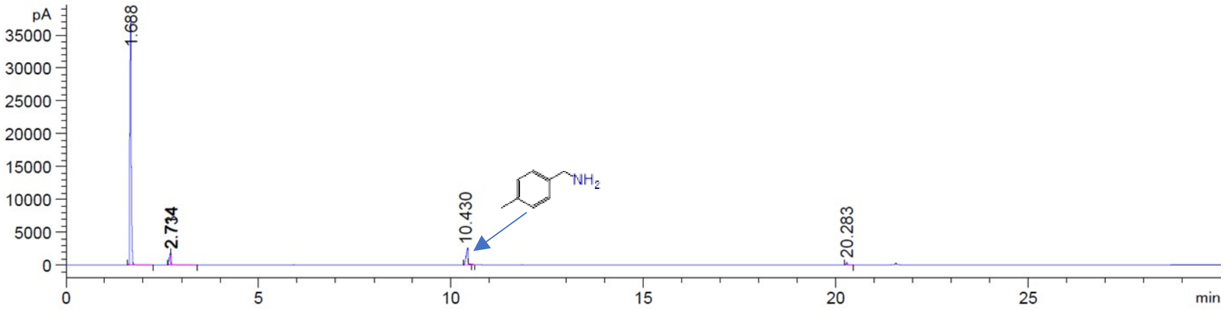

(4-Ethylphenyl)methanamine

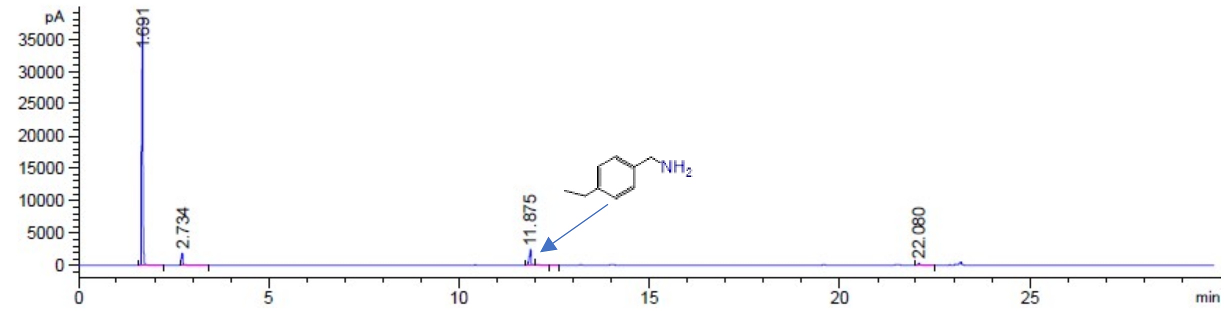

4-tert-Butylbenzylamine

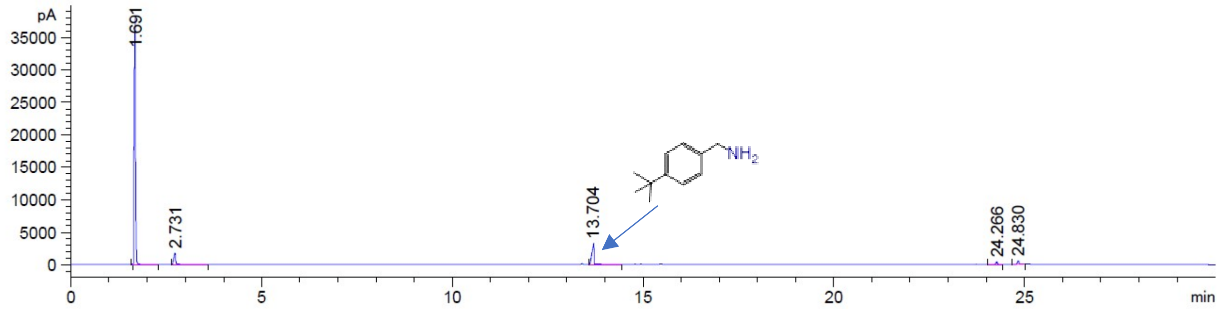

(4-Fluorophenyl)methanamine

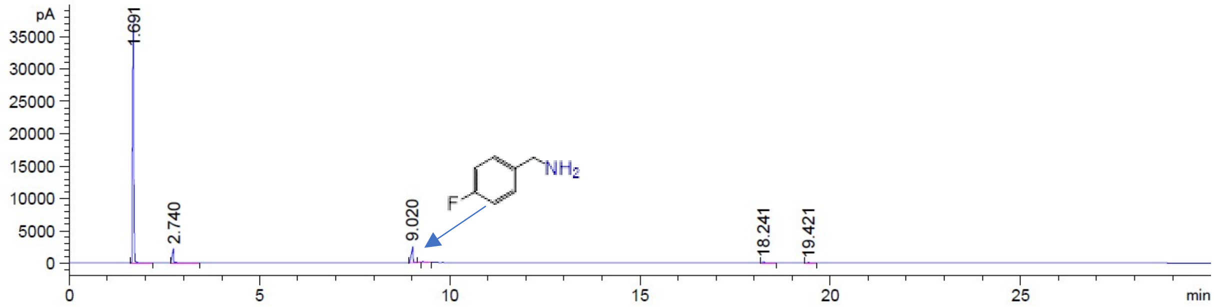

(4-Chlorophenyl)methanamine

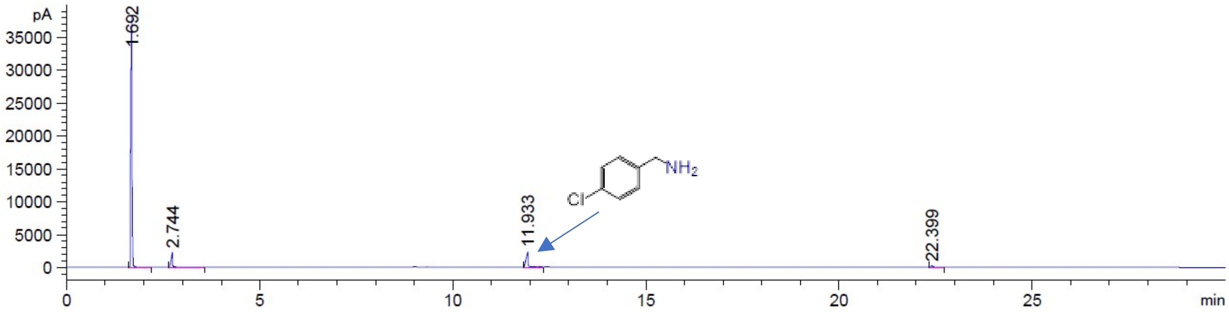

(4-Bromophenyl)methanamine

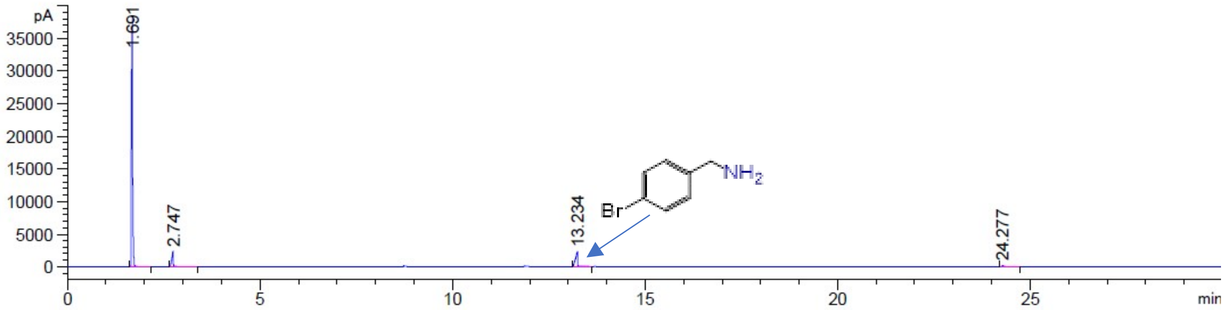

(4-Methoxyphenyl)methanamine

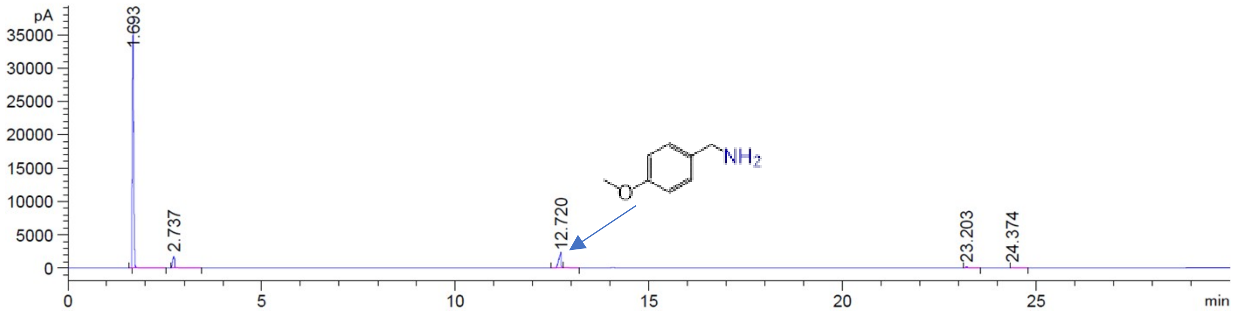

(3,4-Dimethoxyphenyl)methanamine

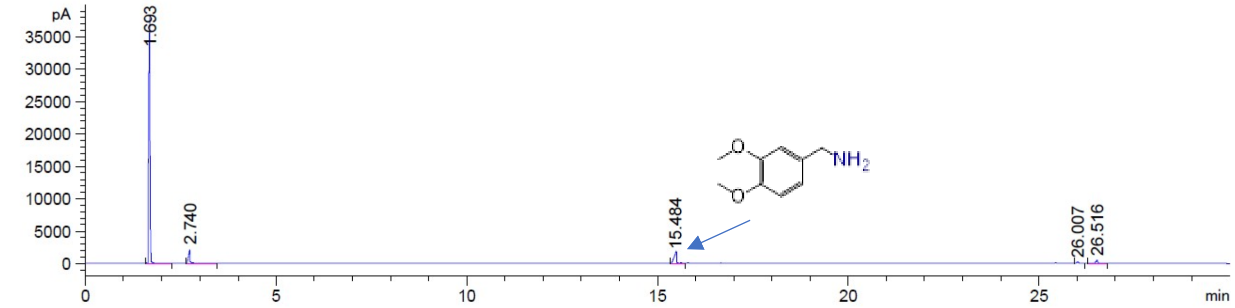

N-(4-(aminomethyl)phenyl)acetamide

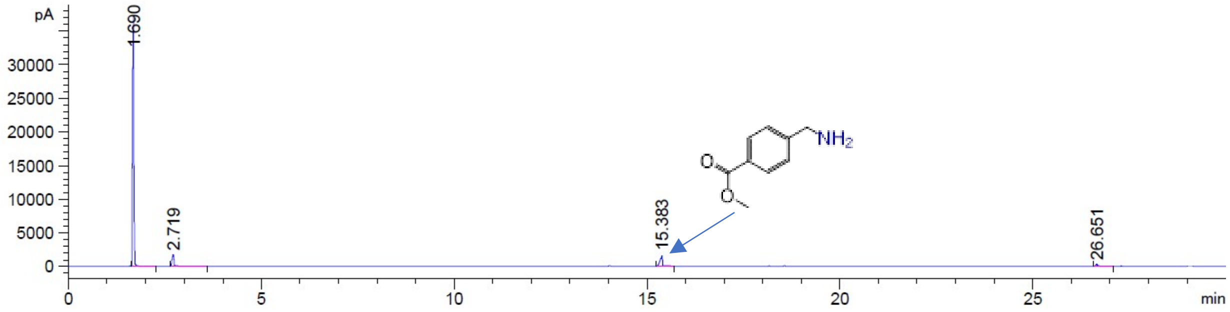

Methyl 4-(aminomethyl)benzoate

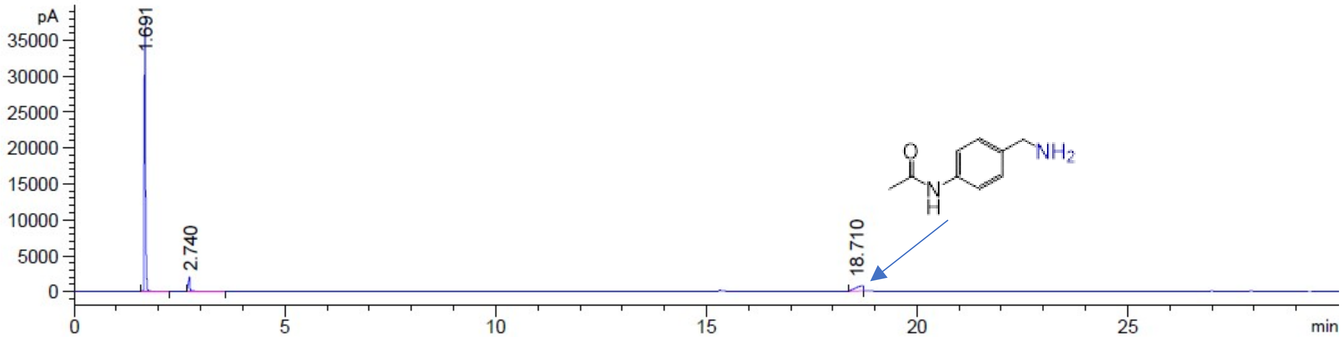

3,7-dimethyloct-6-en-1-amine

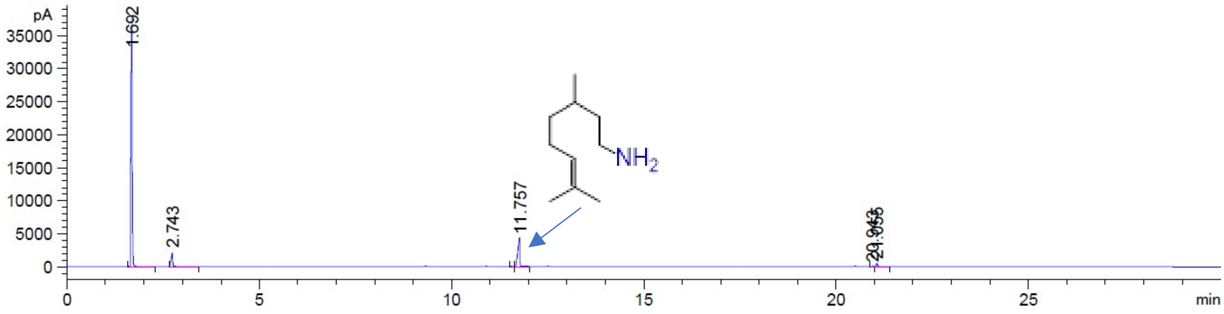

(5-(Aminomethyl)furan-2-yl)methanol

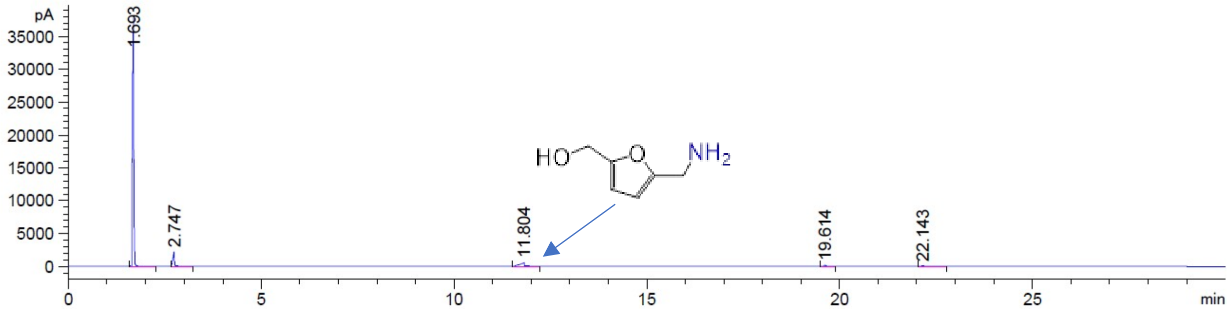

Androsterone-NH_2

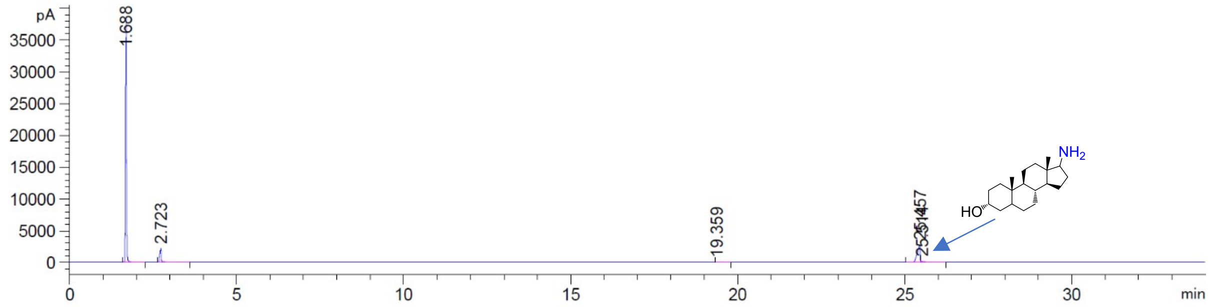

Copies of ^{1}H NMR and ^{13}C NMR

Cyclopentylamine

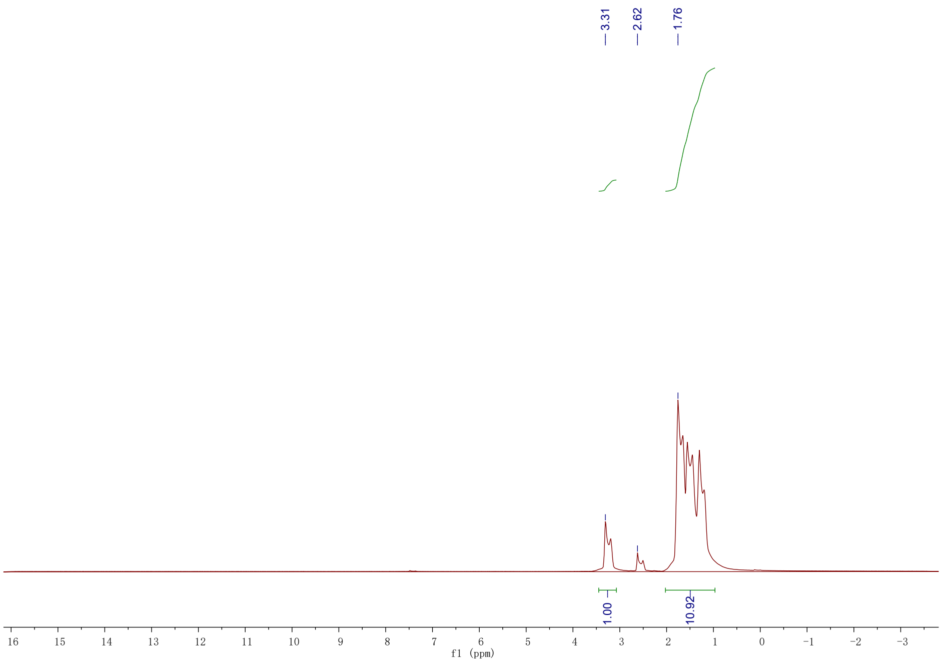

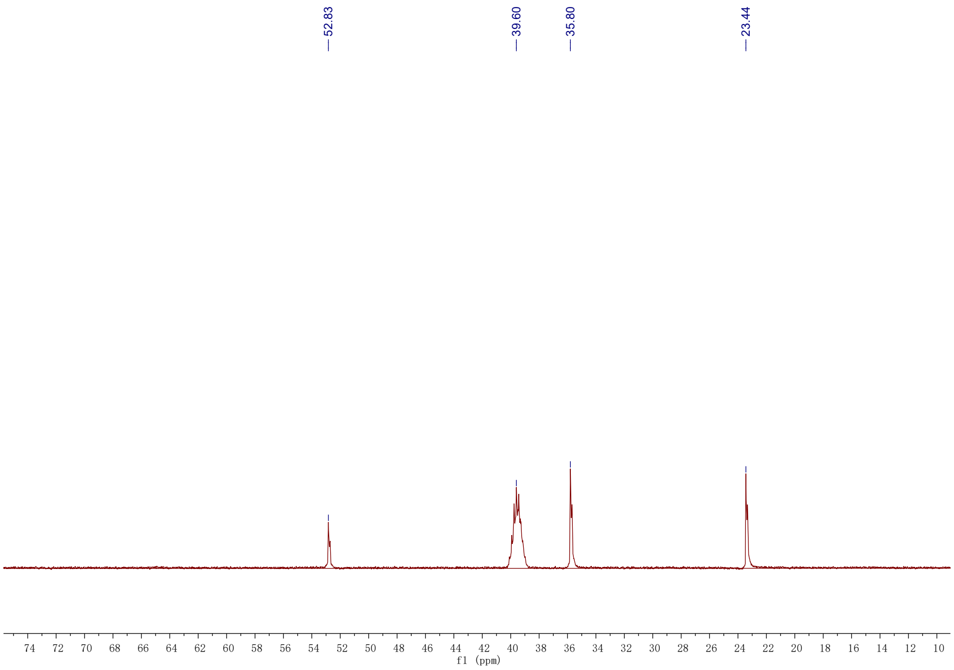

Cyclohexanamine

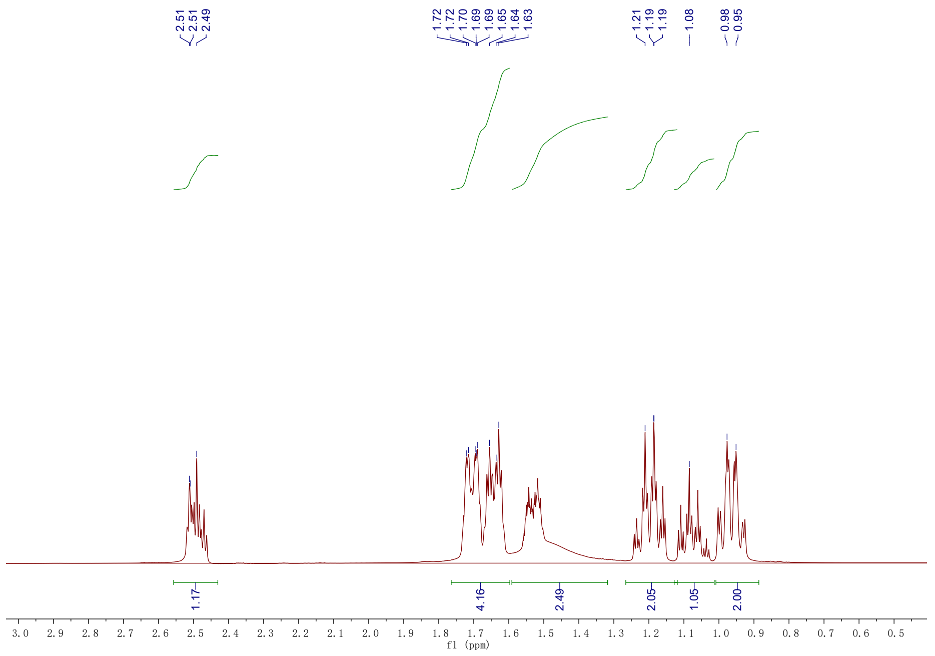

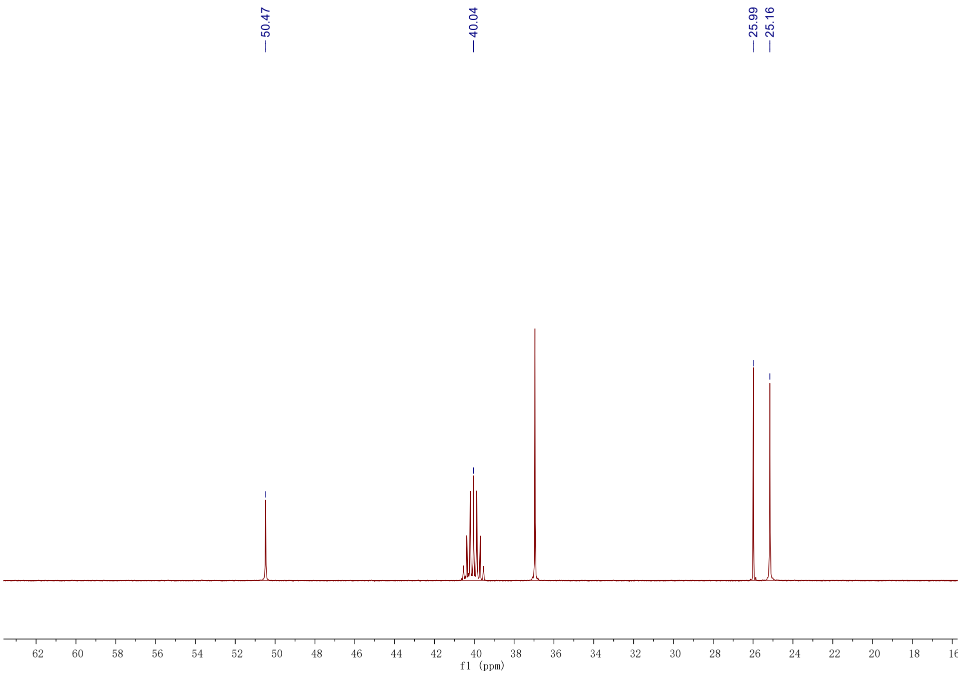

Cycloheptanamine

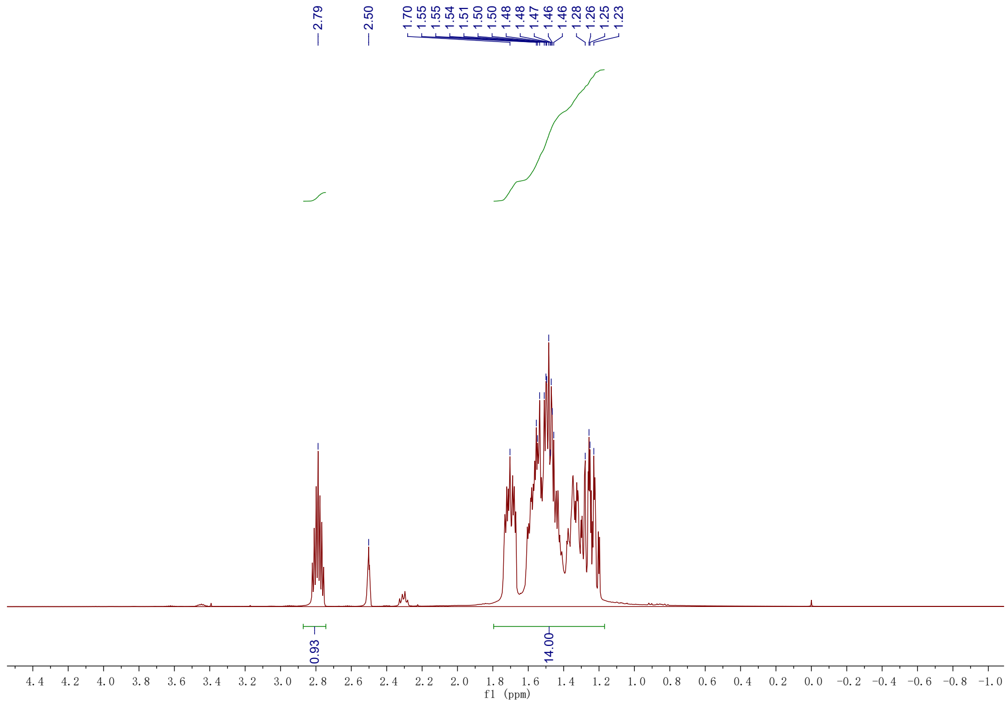

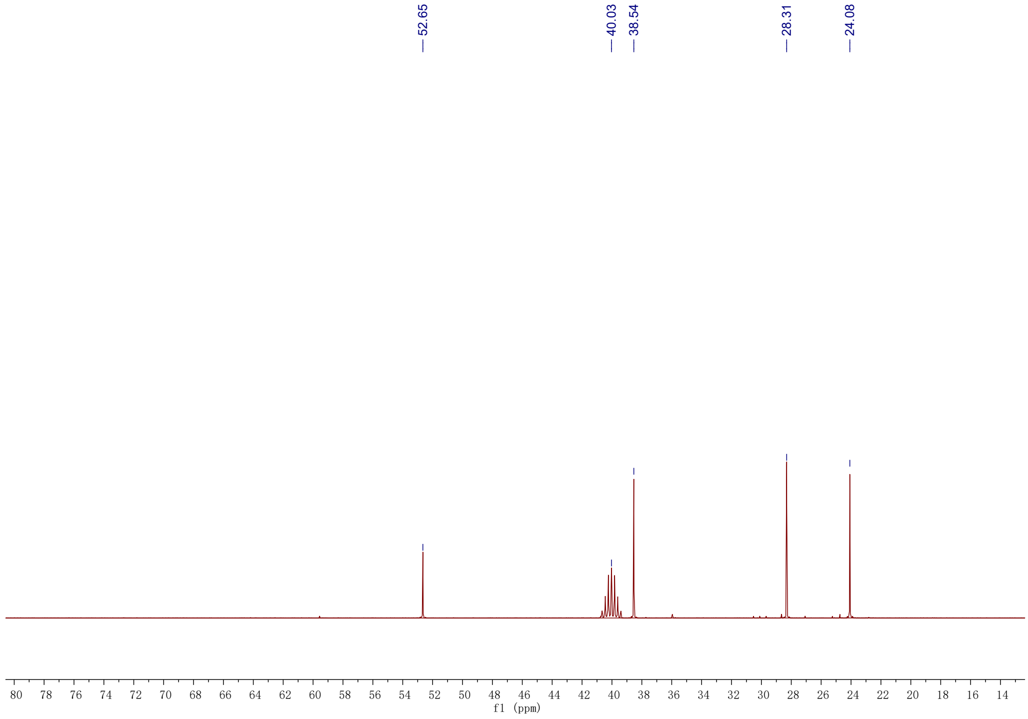

4-Methylpentan-2-amine

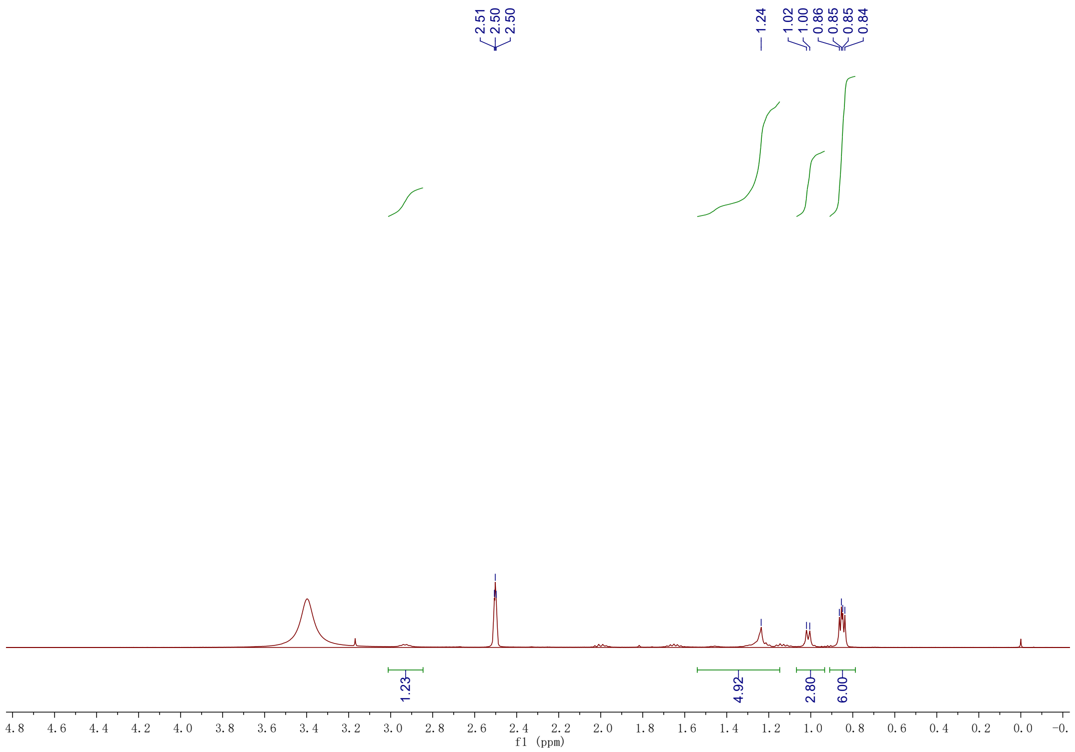

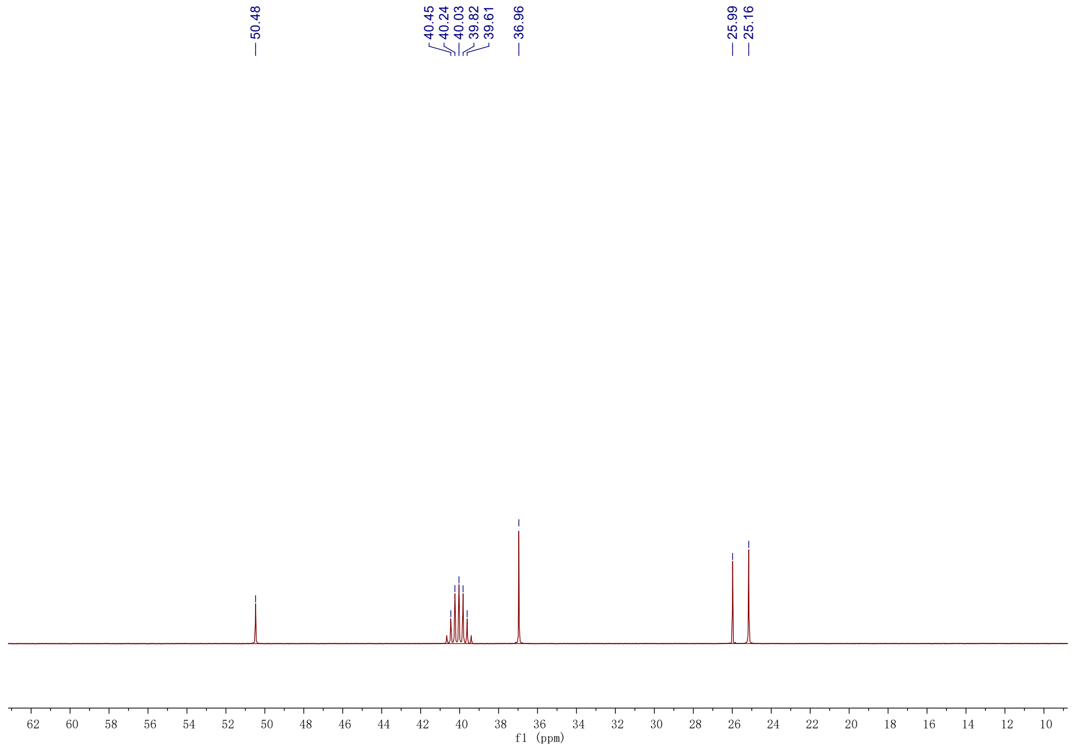

Pentan-3-amine

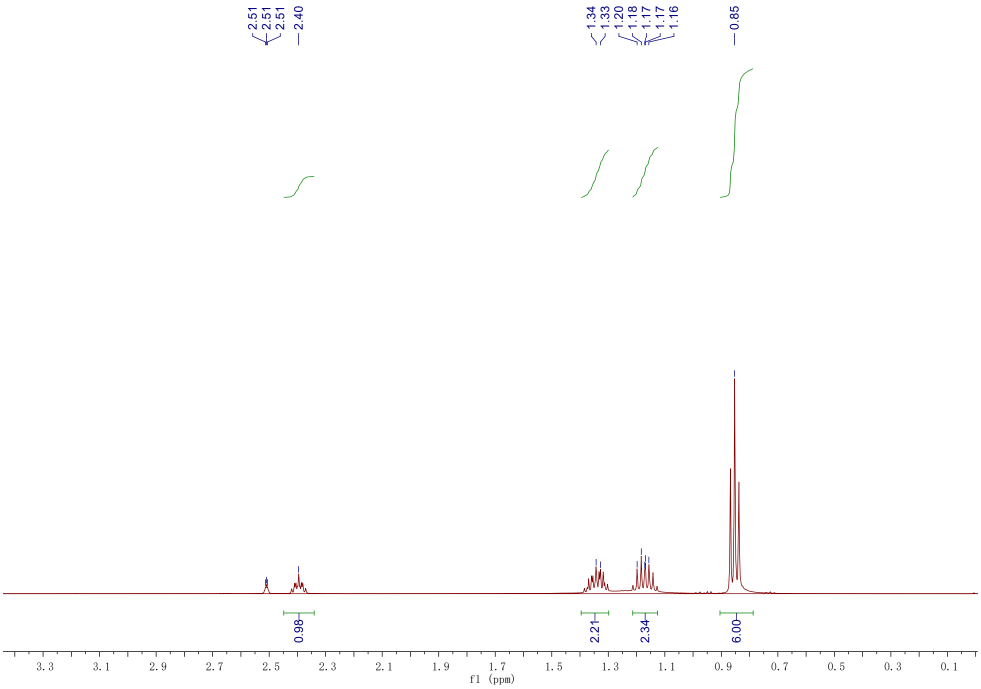

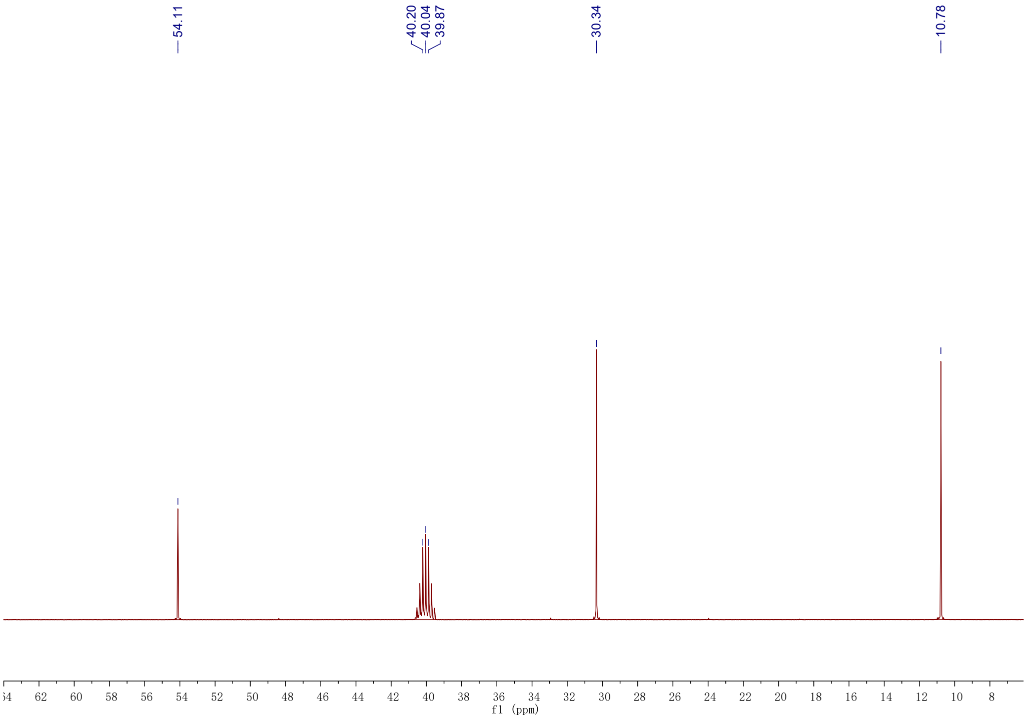

Octan-2-amine

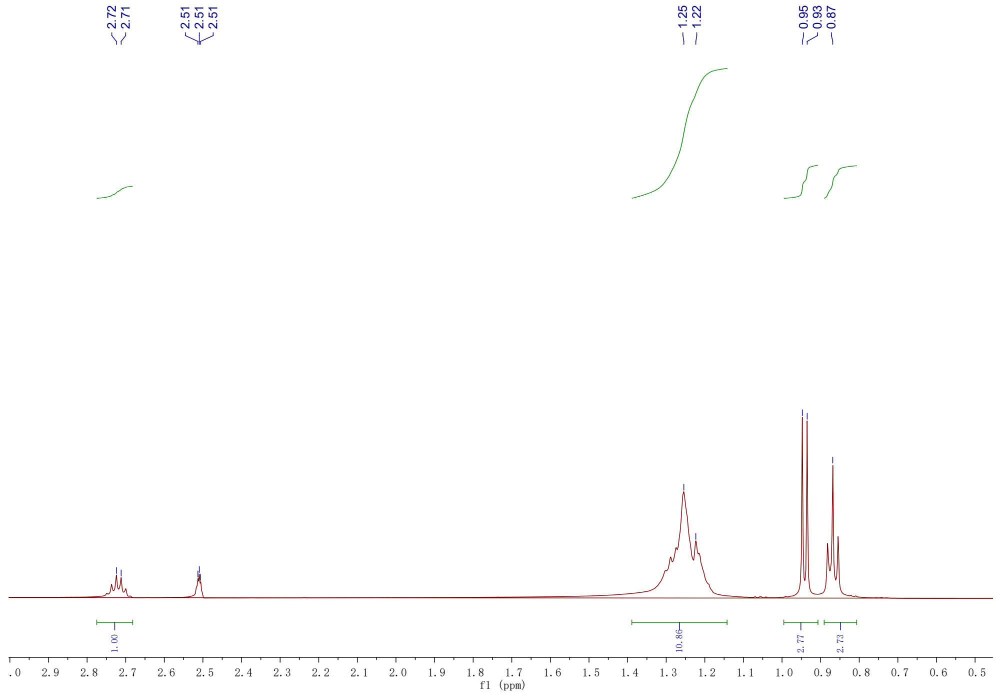

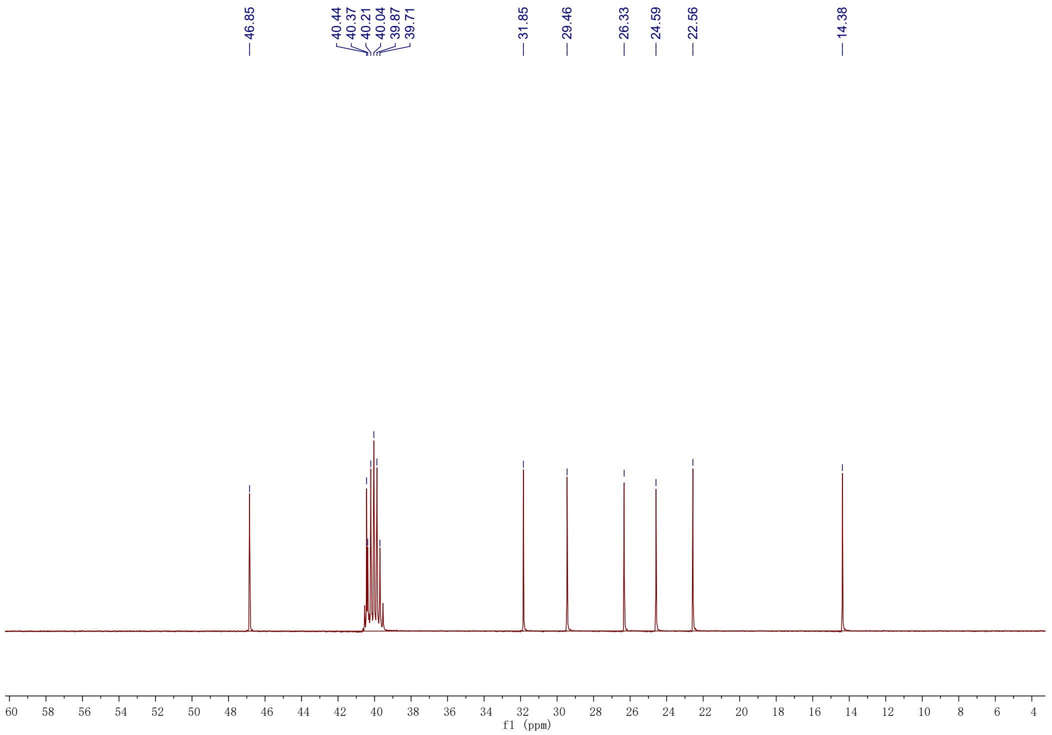

Nonan-2-amine

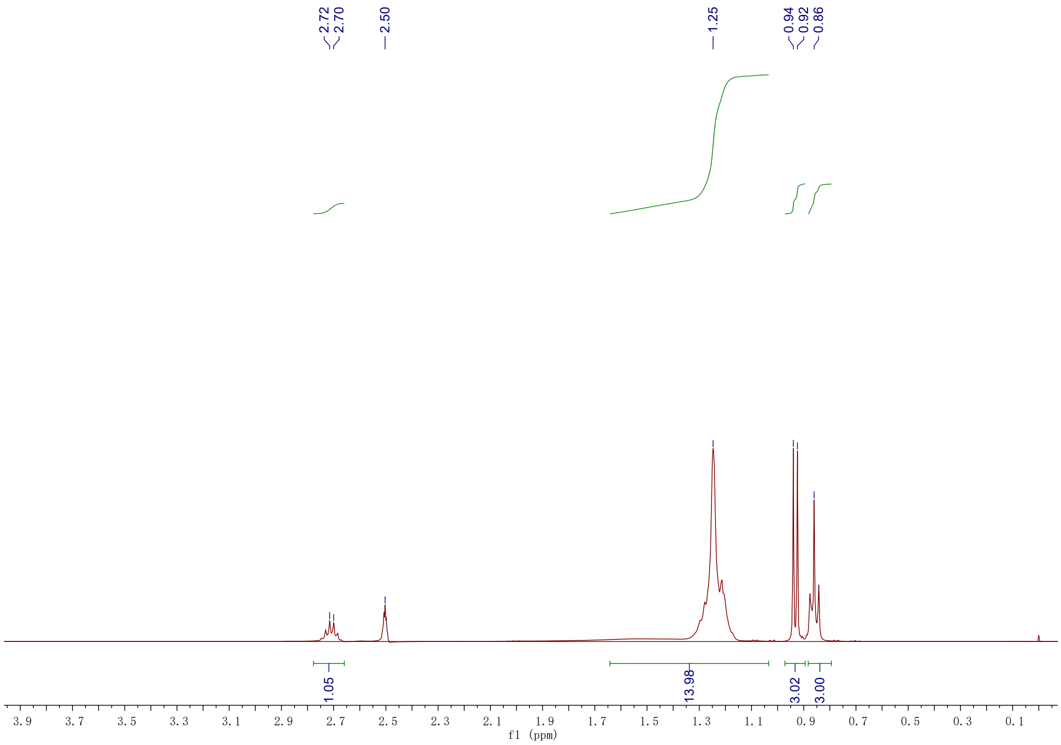

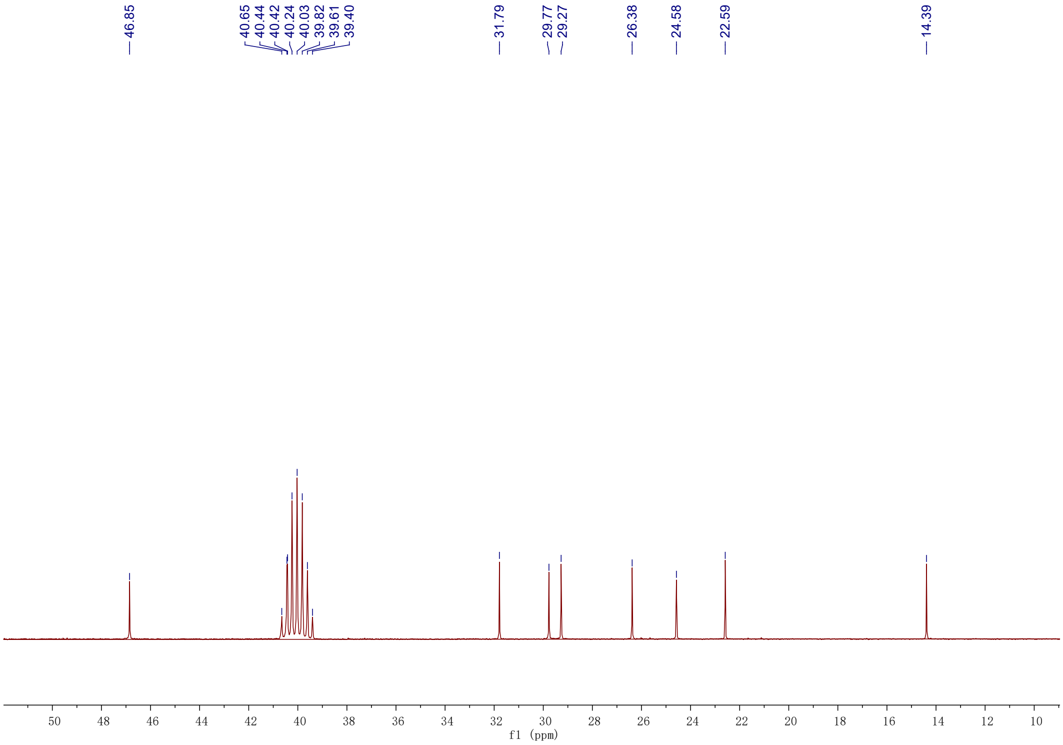

4-Phenylbutan-2-amine

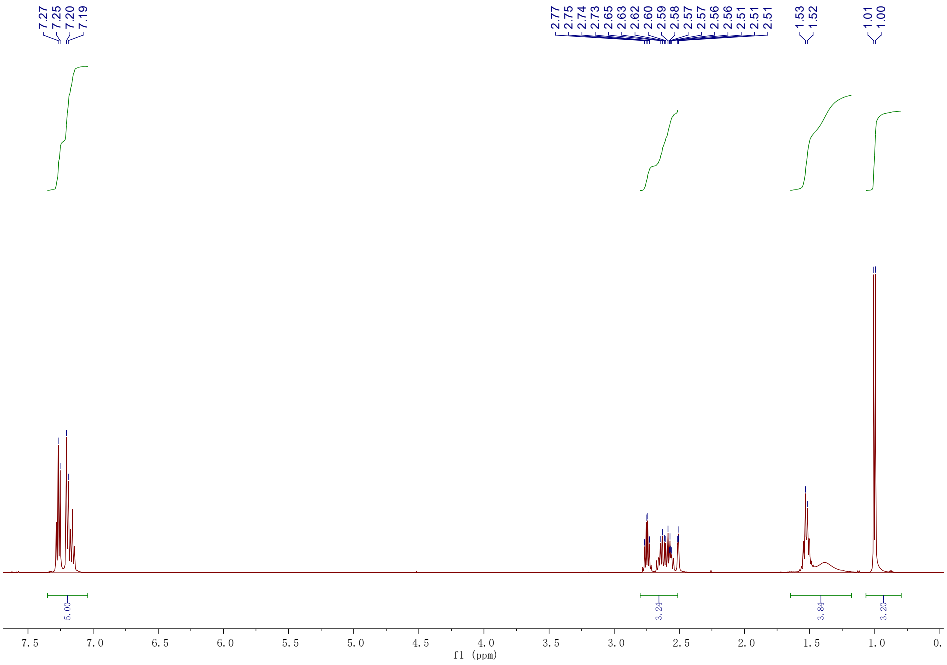

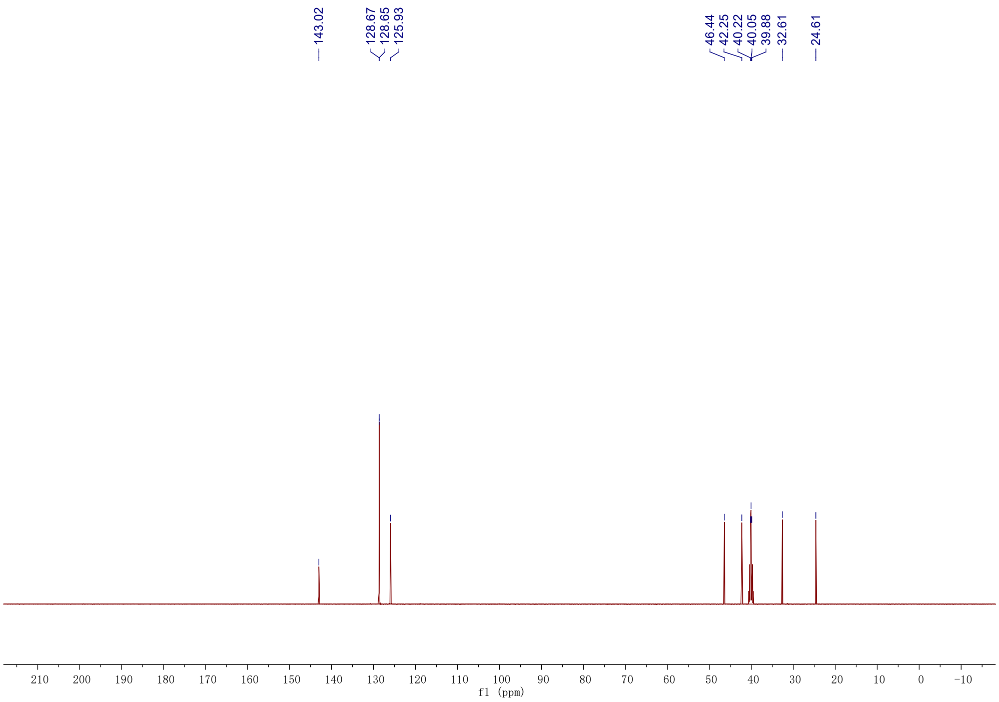

1-Adamantanamine

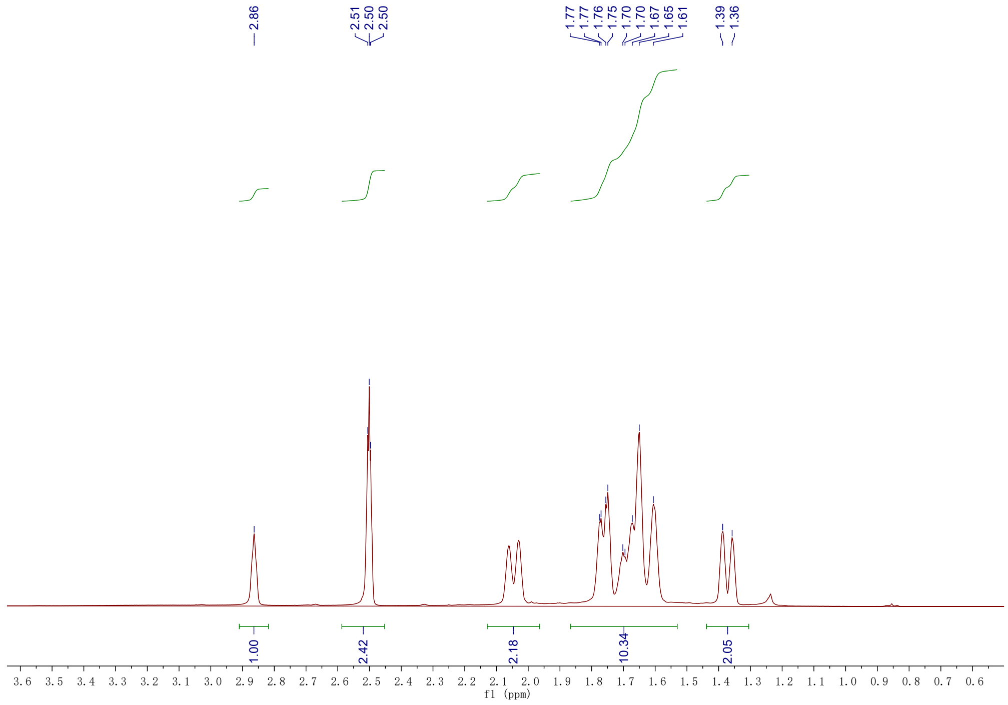

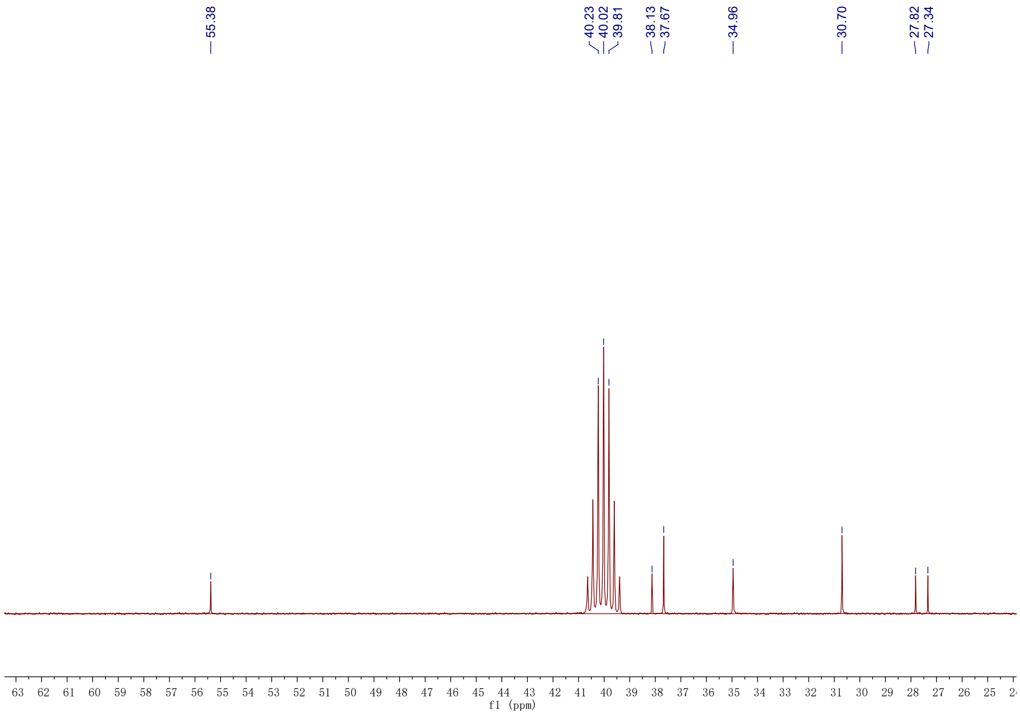

1-Phenylethan-1-amine

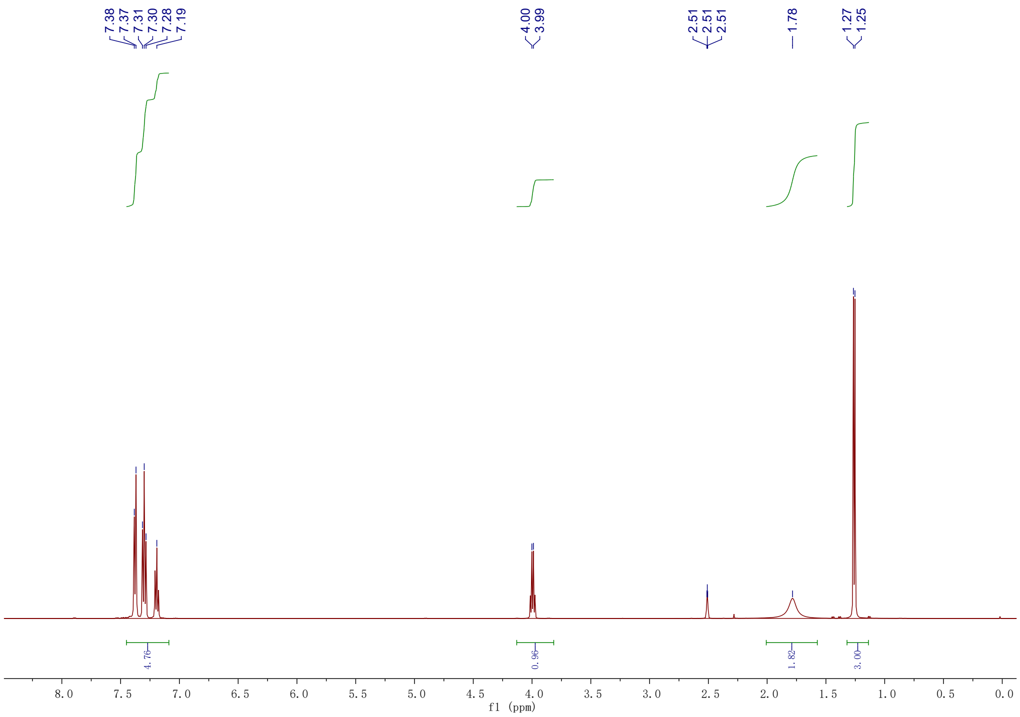

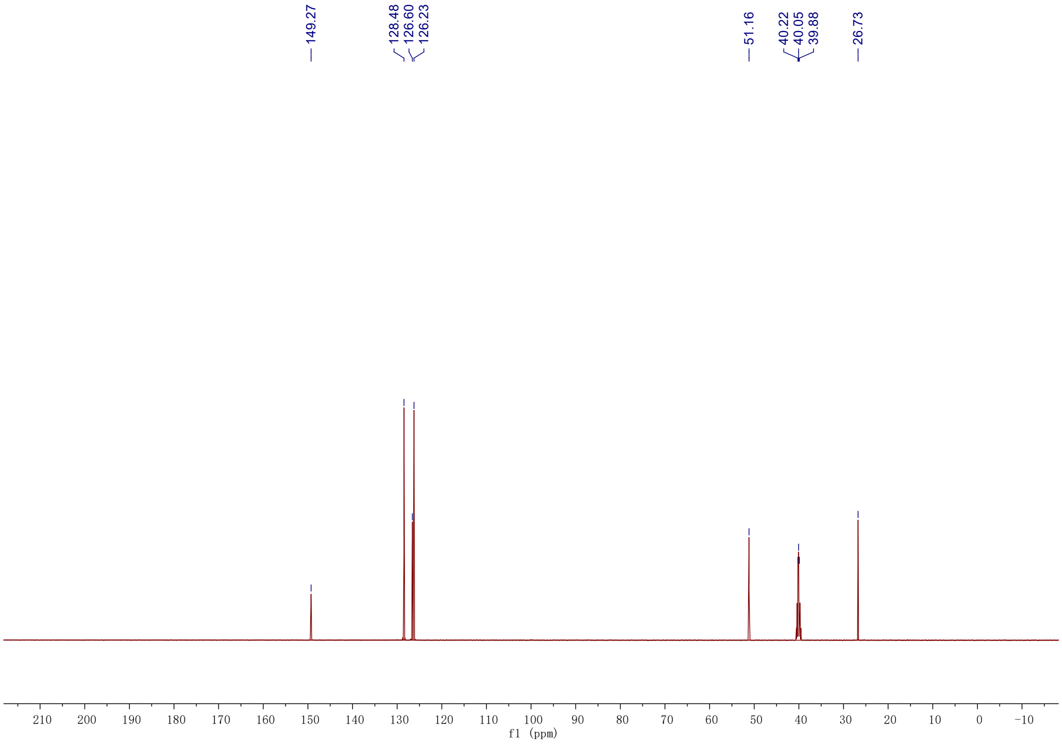

n-Butylamine

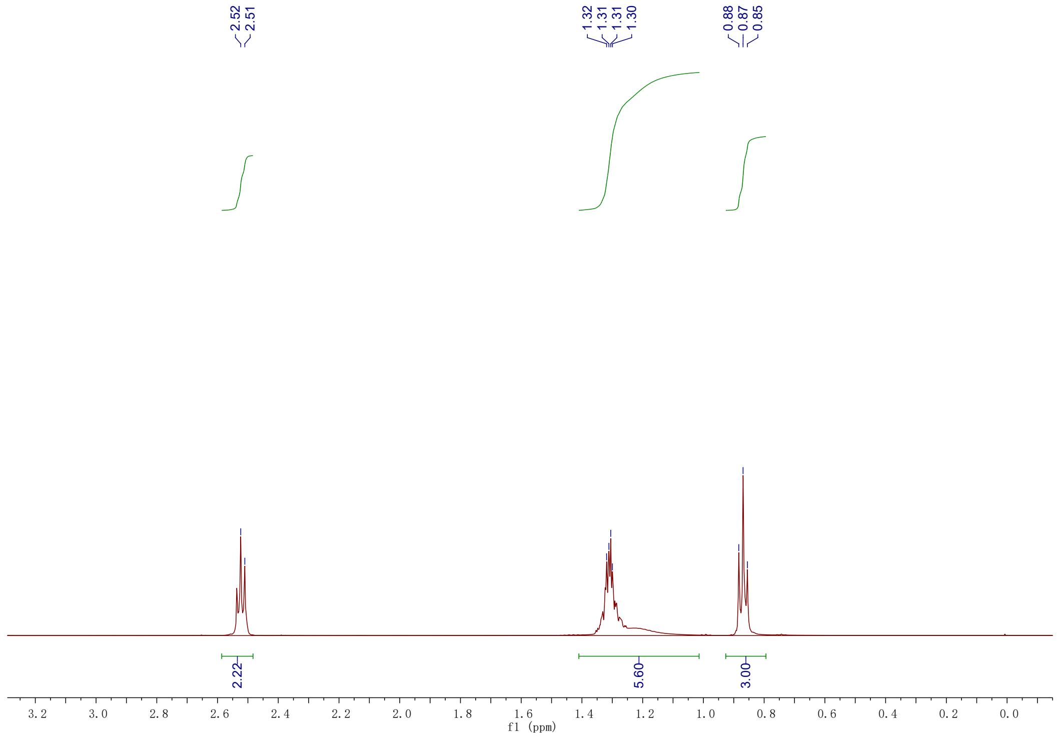

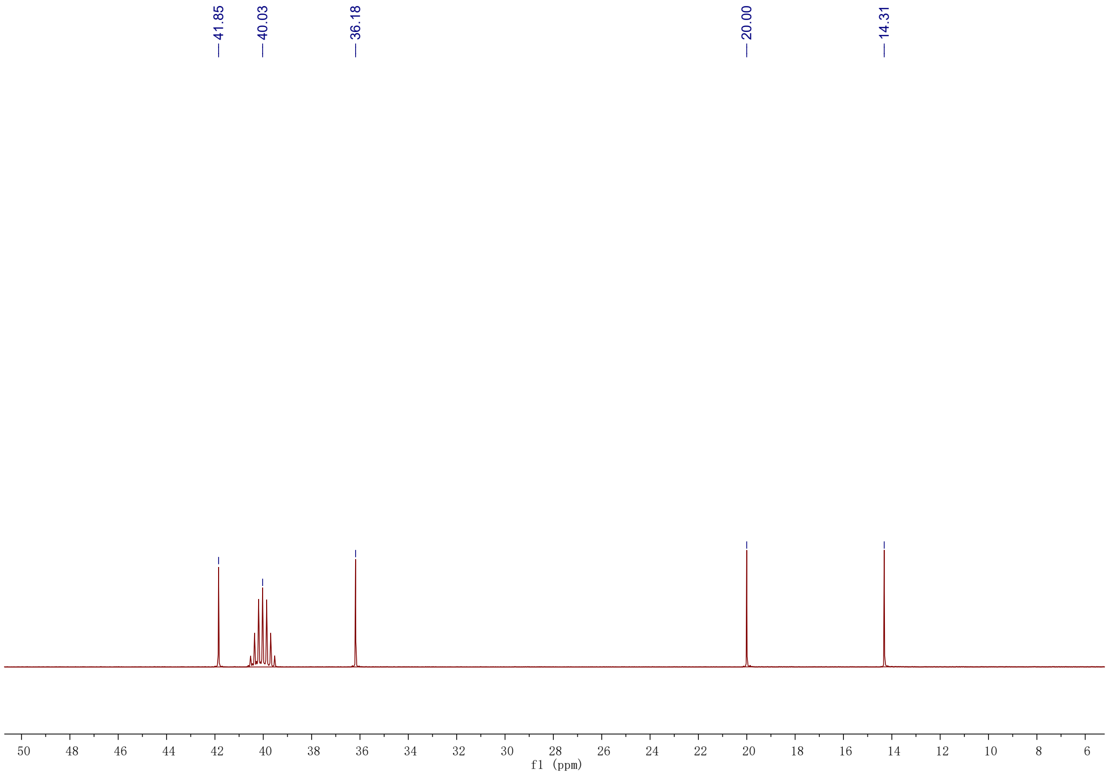

_n_-hexylamine

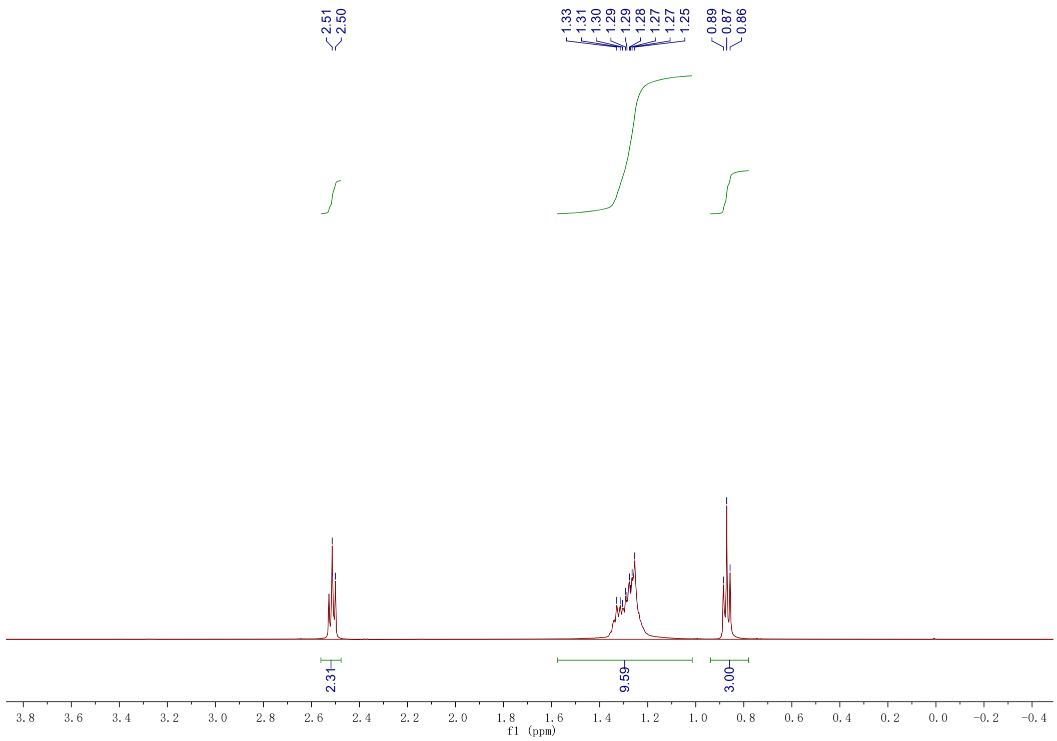

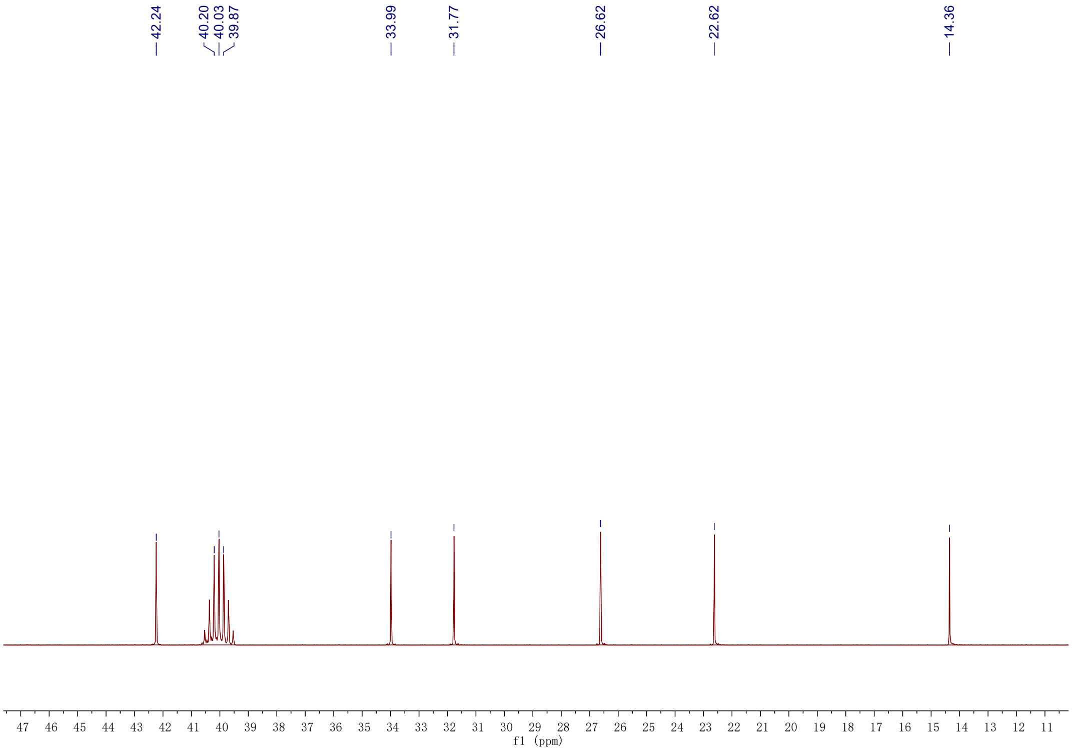

Octan-1-amine

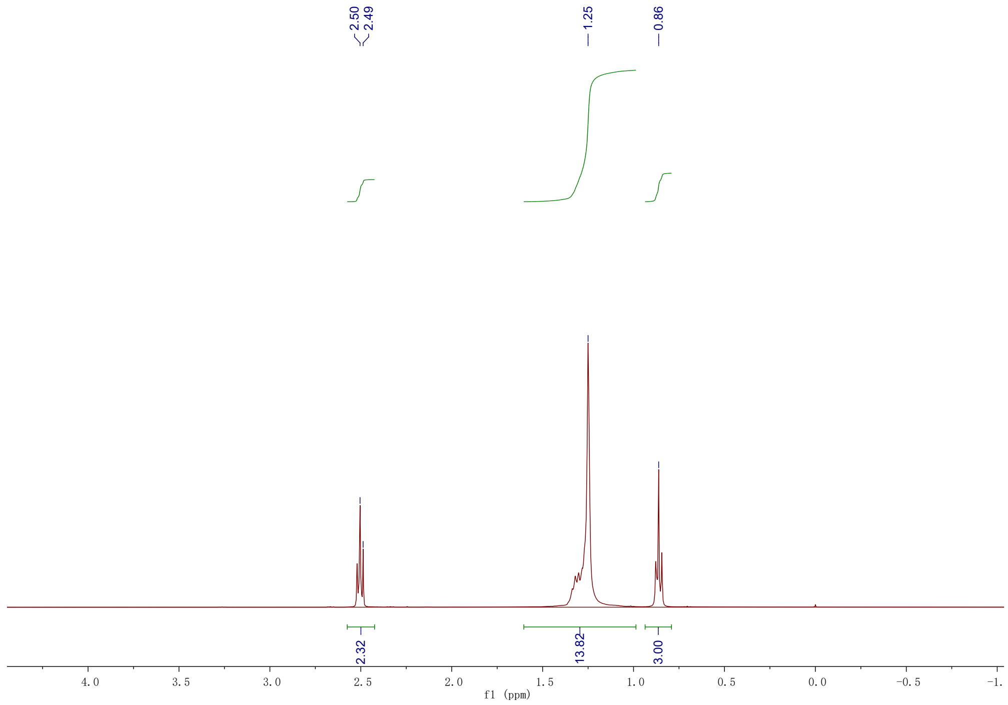

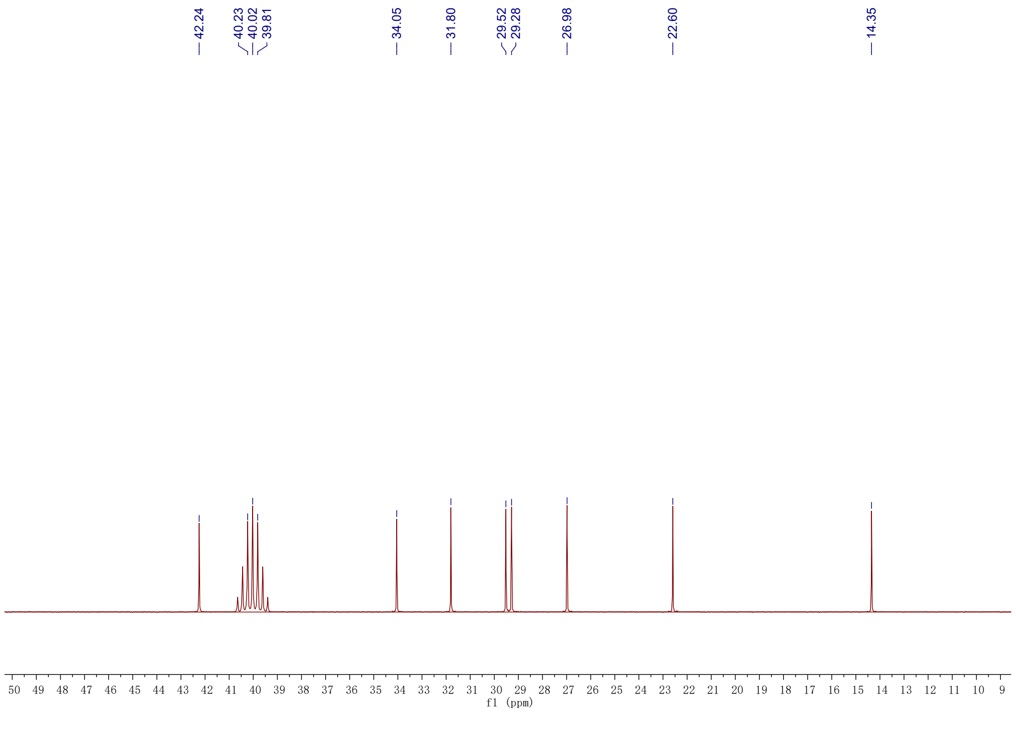

3-Phenylpropan-1-amine

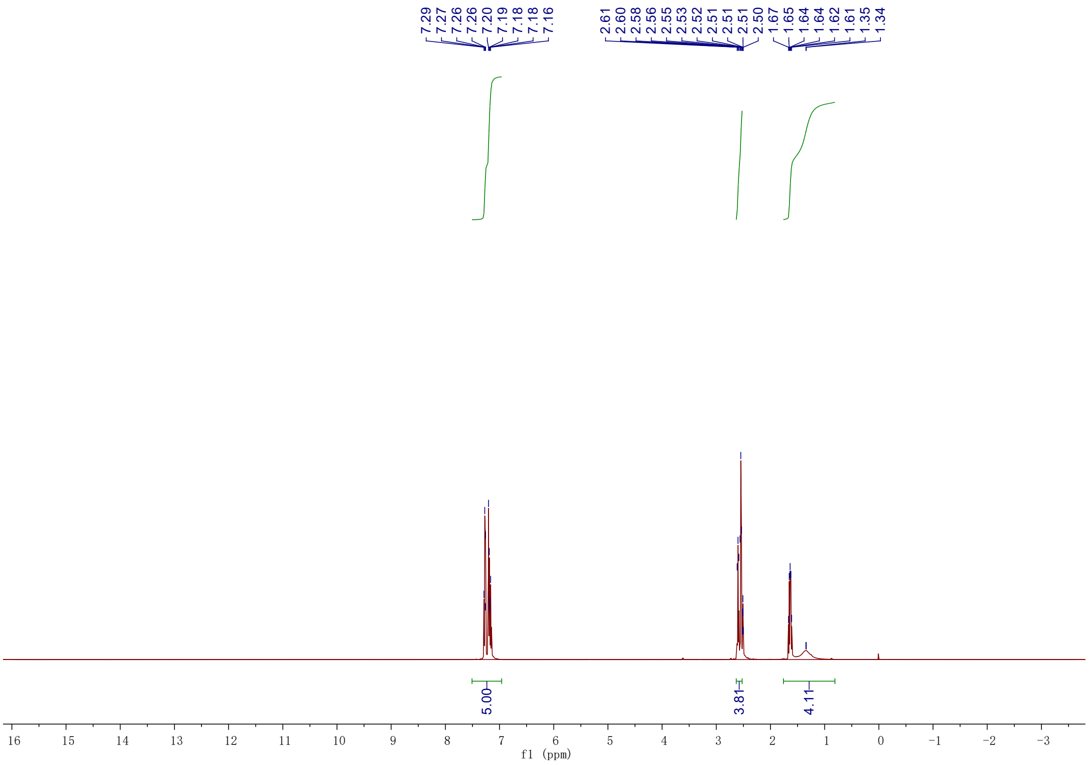

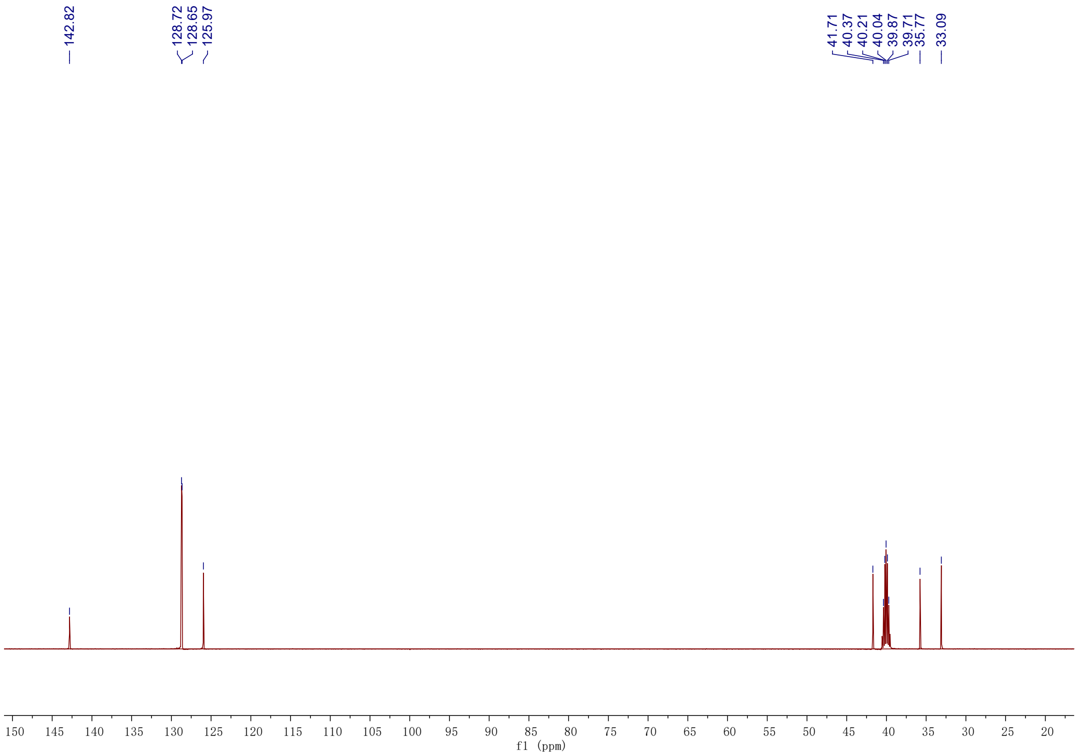

Phenylmethanamine

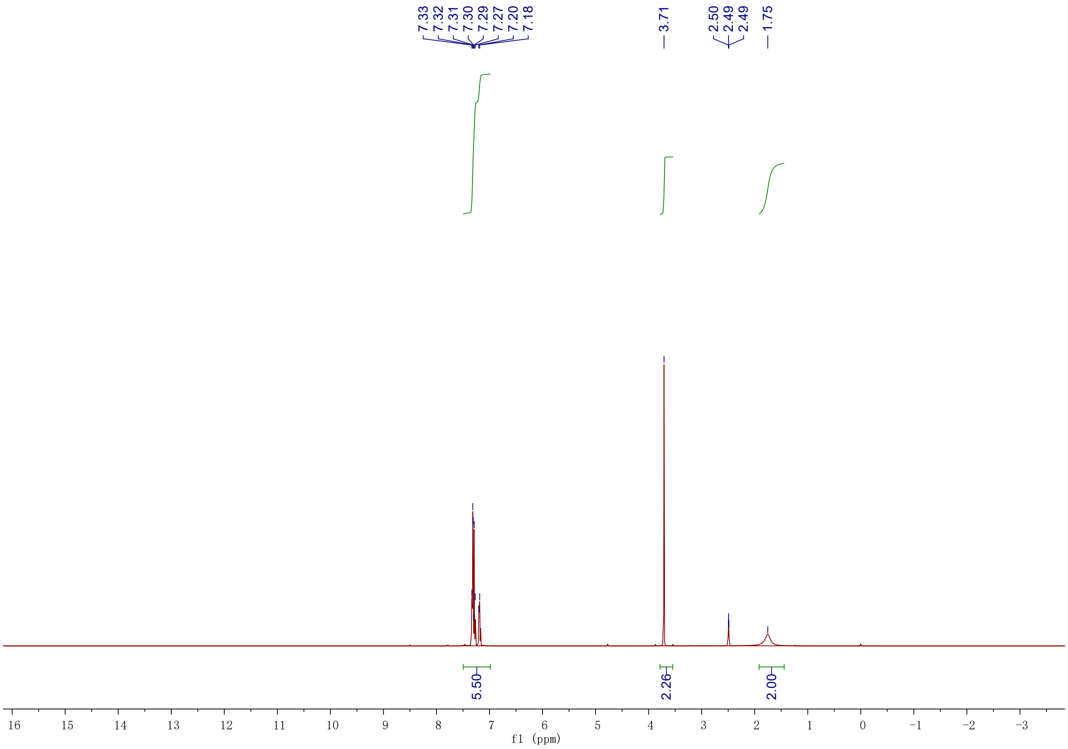

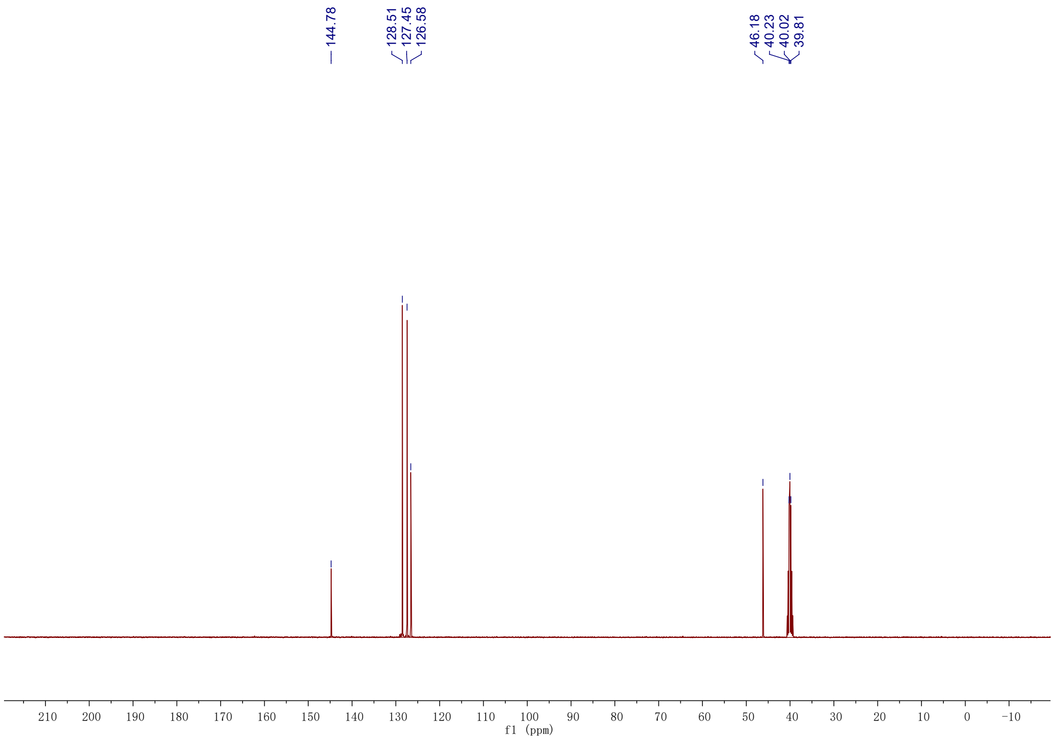

Pyridin-3-ylmethanamine

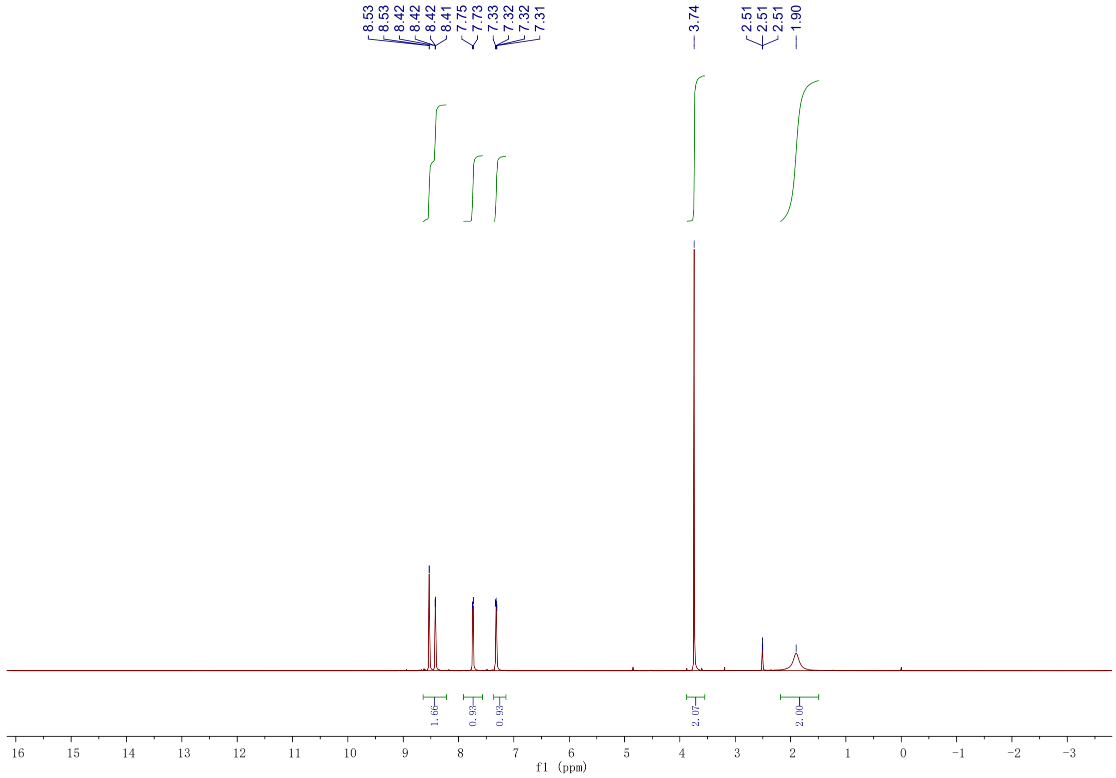

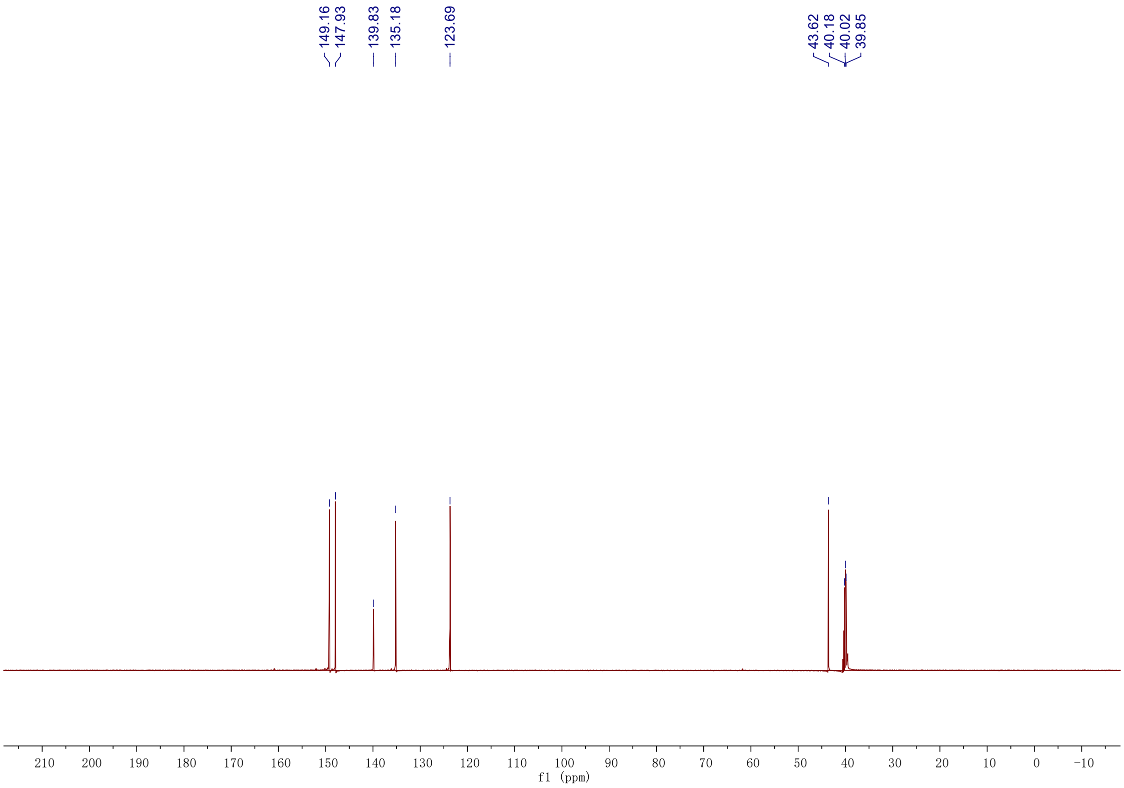

Furan-2-ylmethanamine

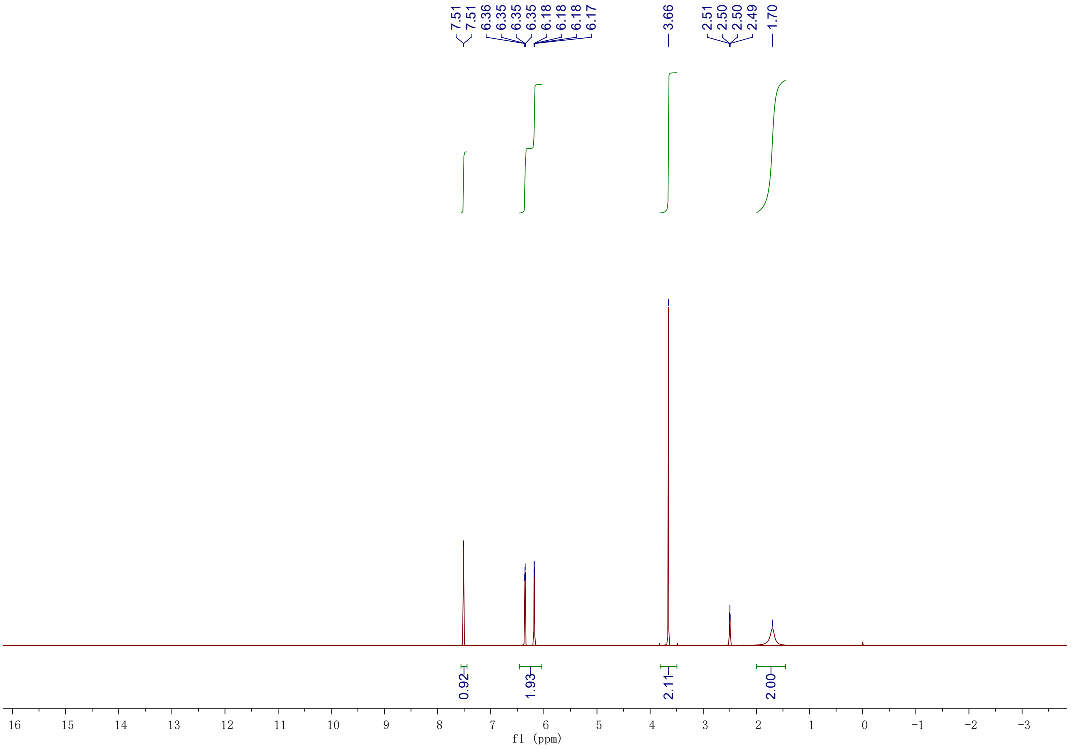

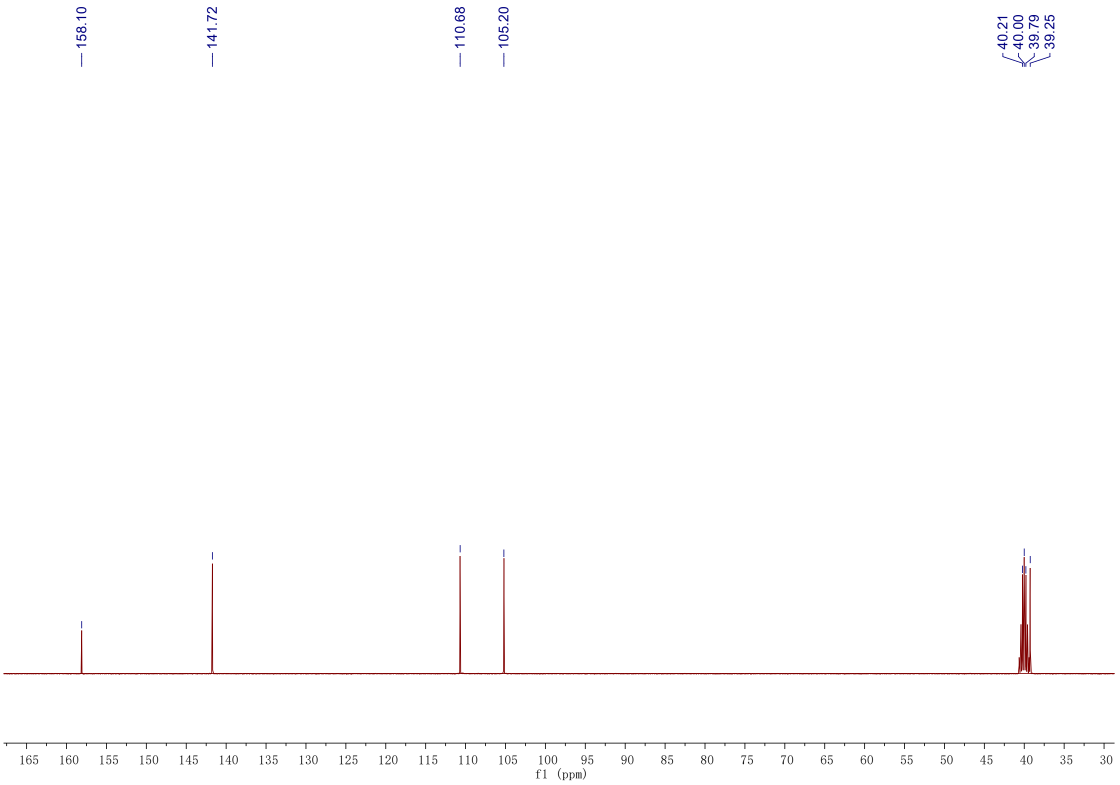

4-Ethylbenzylamine

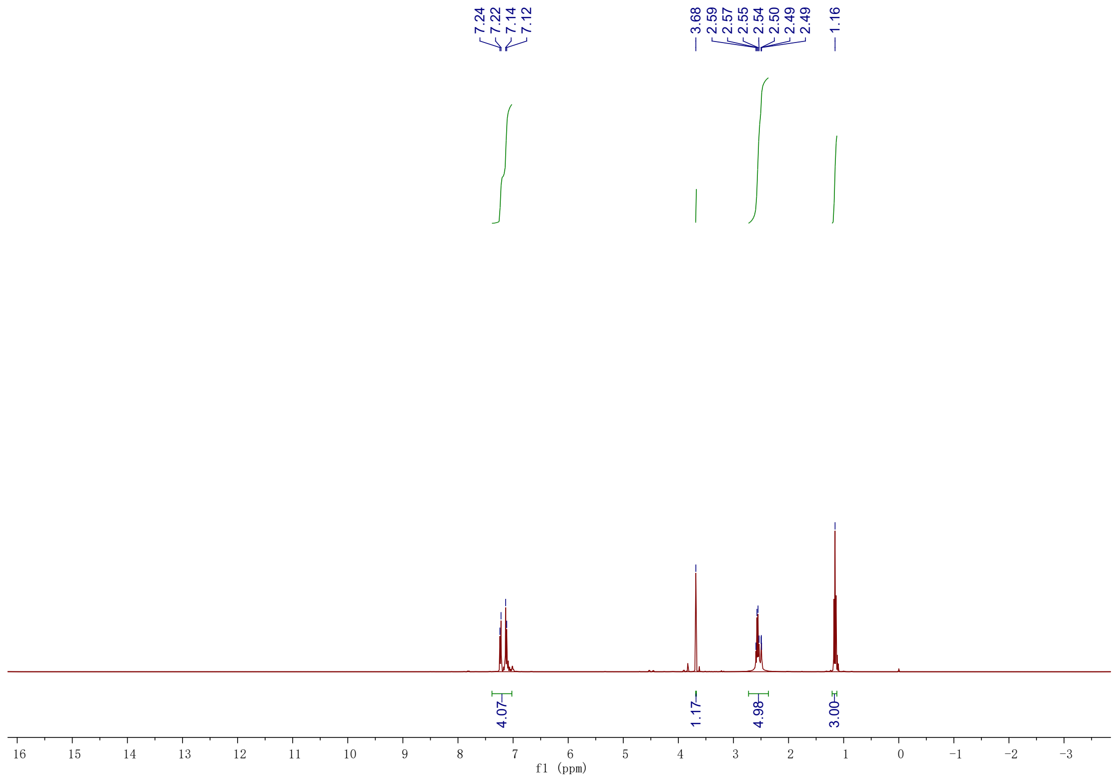

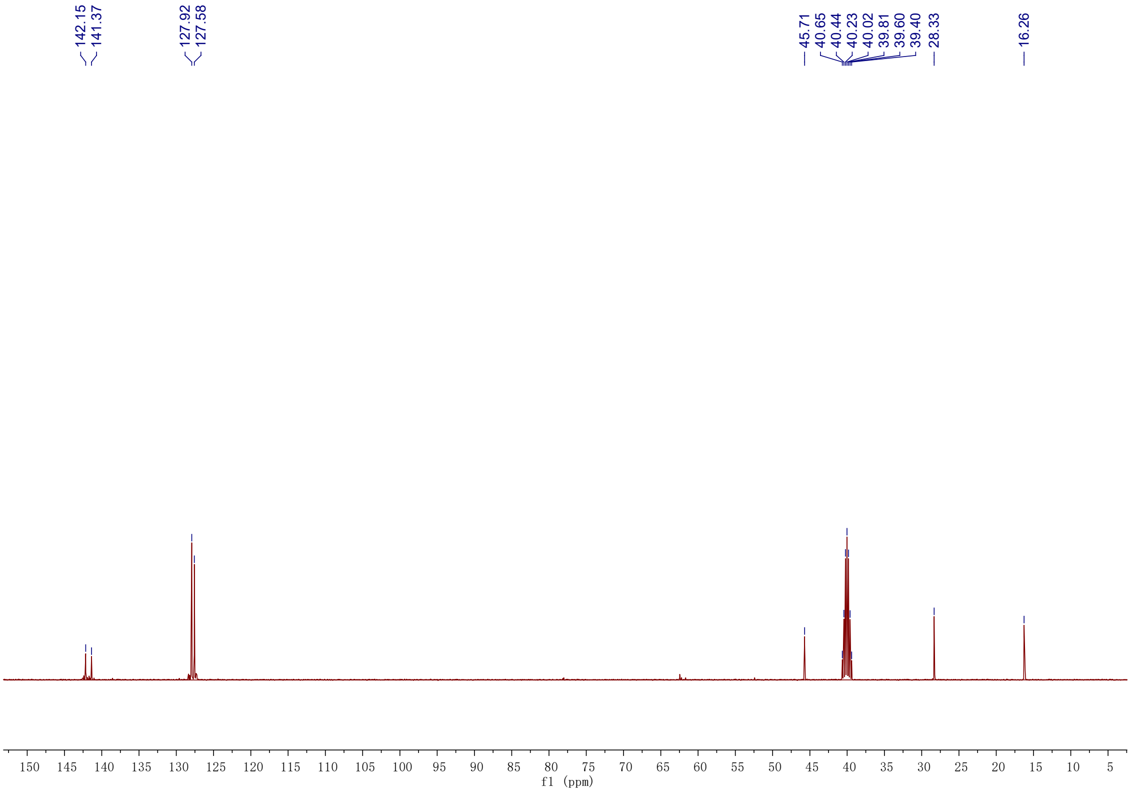

4-tert-Butylbenzylamine

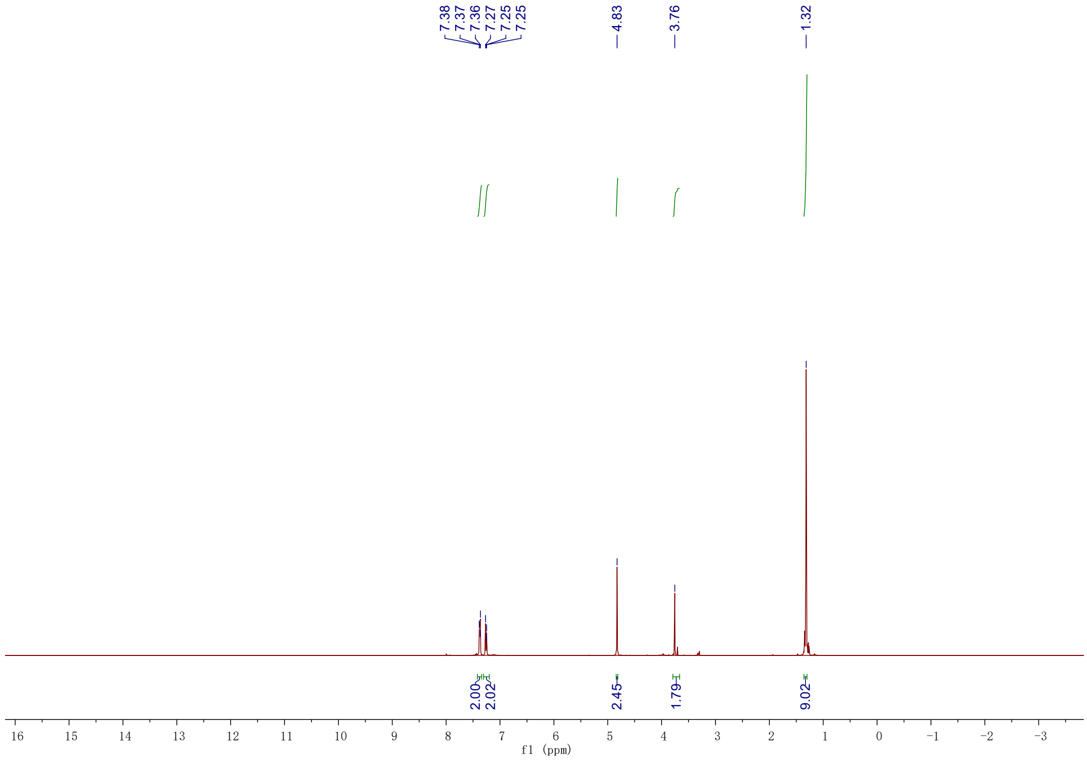

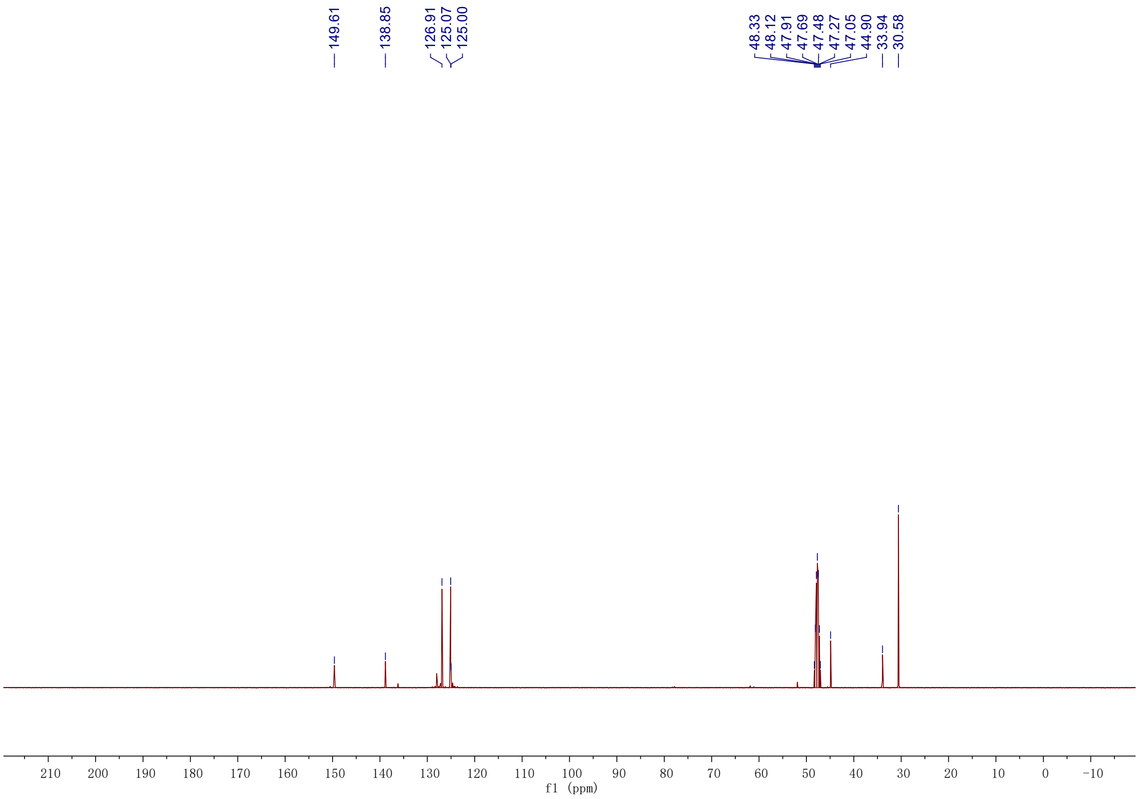

(4-Fluorophenyl)methanamine

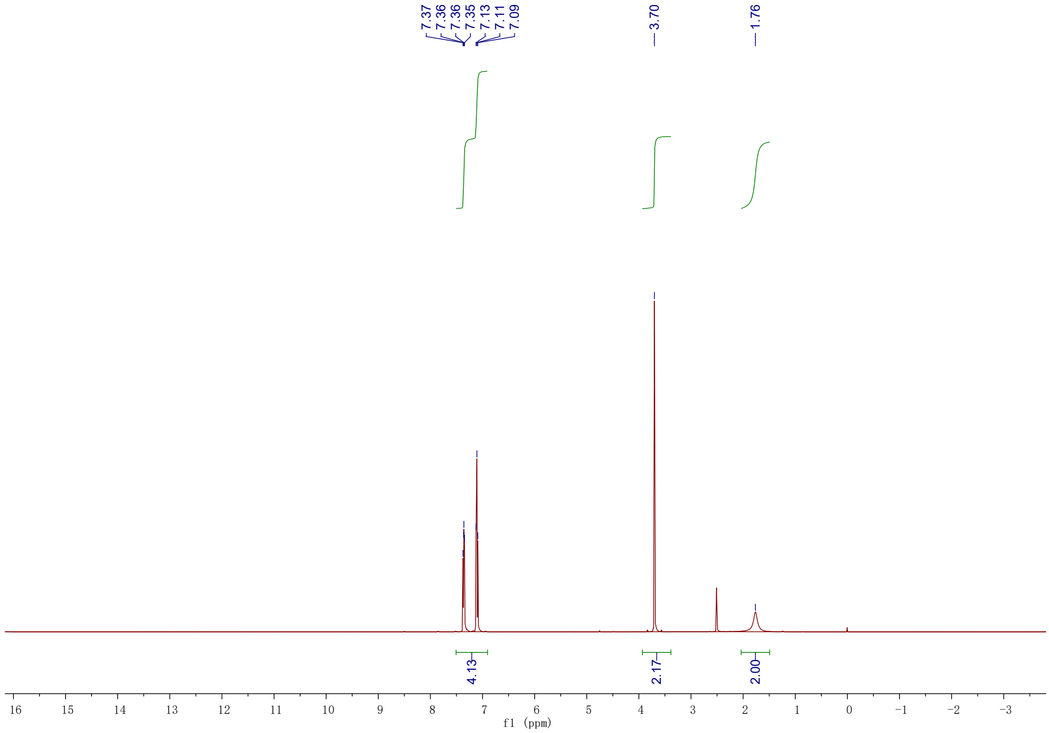

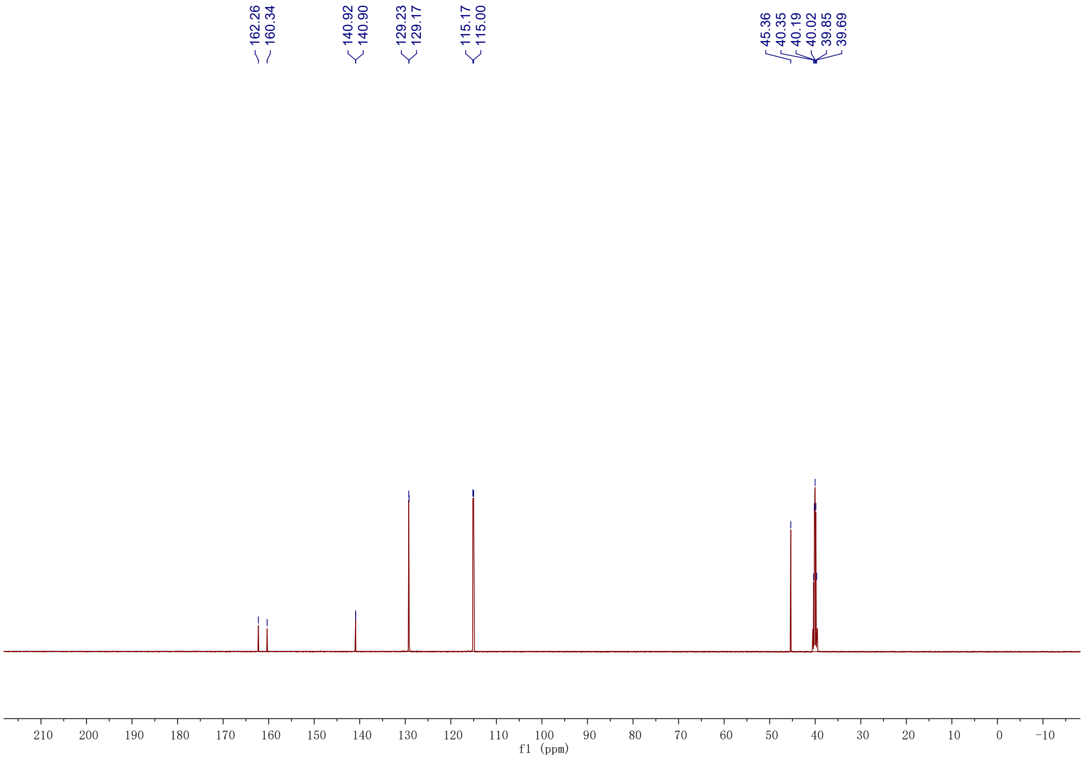

(4-Chlorophenyl)methanamine

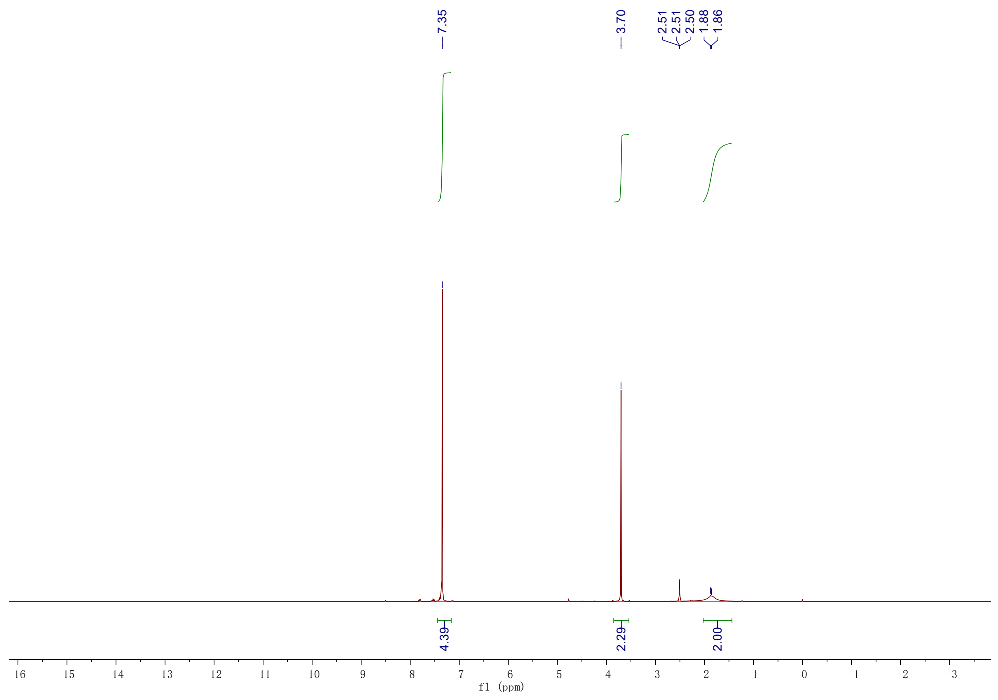

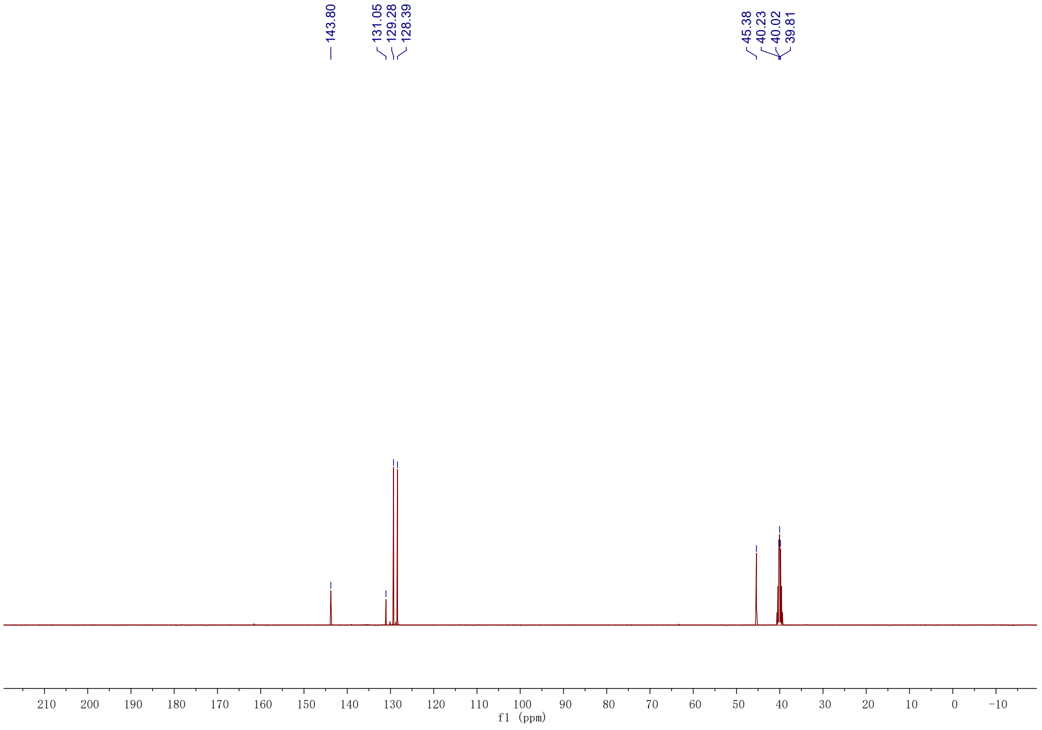

(4-Bromophenyl)methanamine

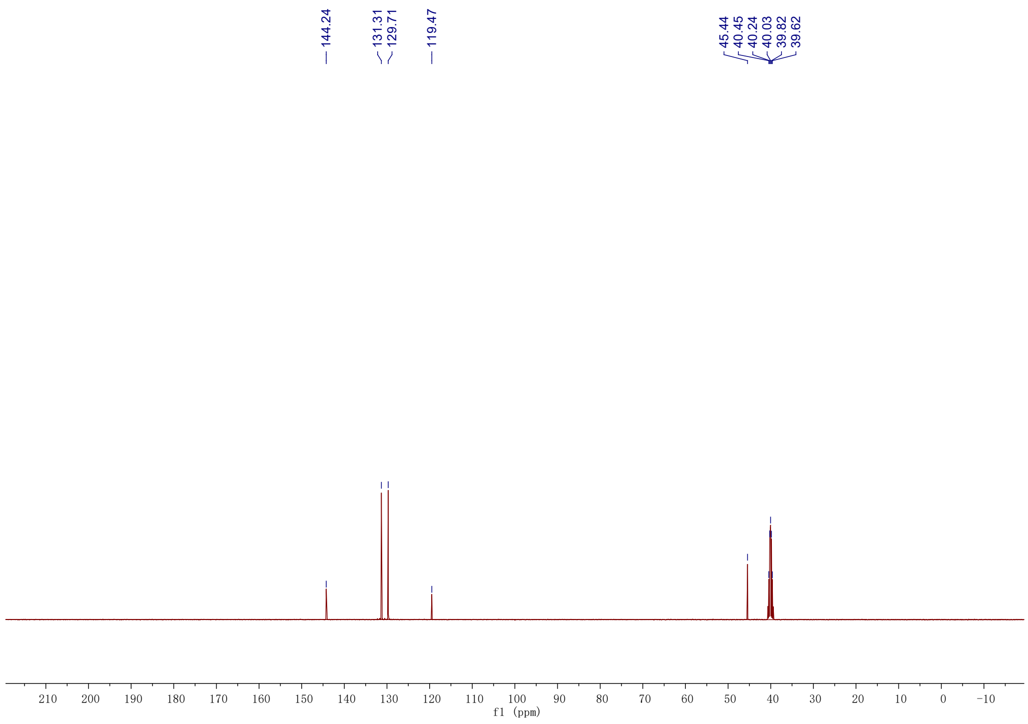

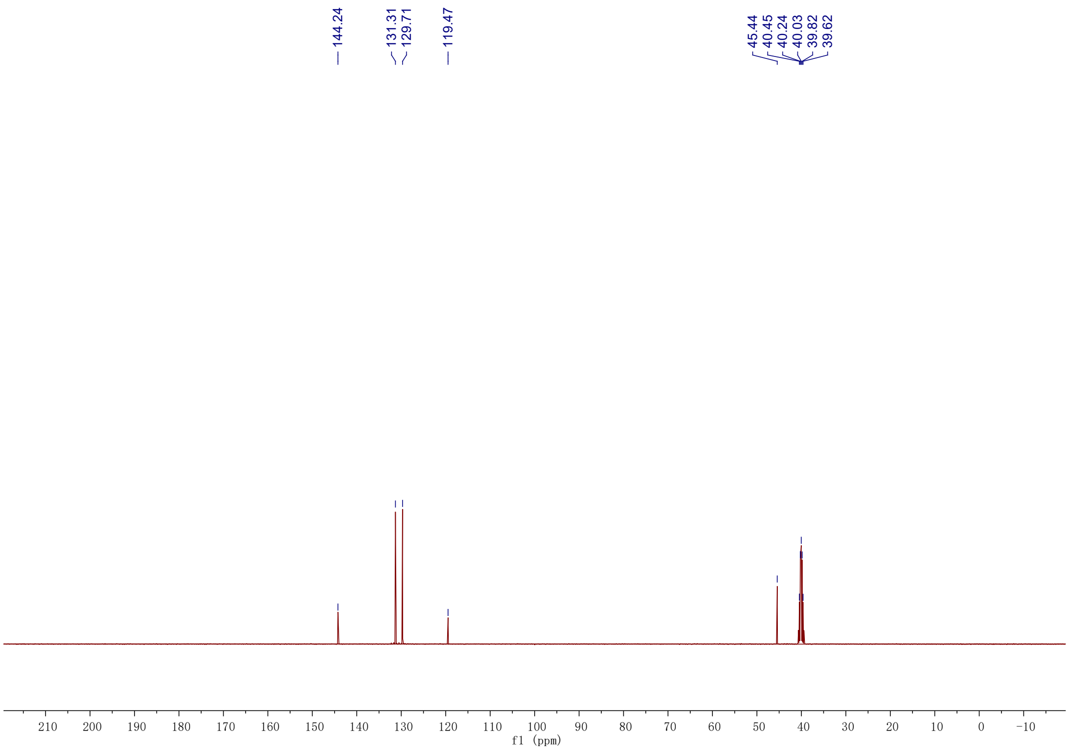

(4-Methoxyphenyl)methanamine

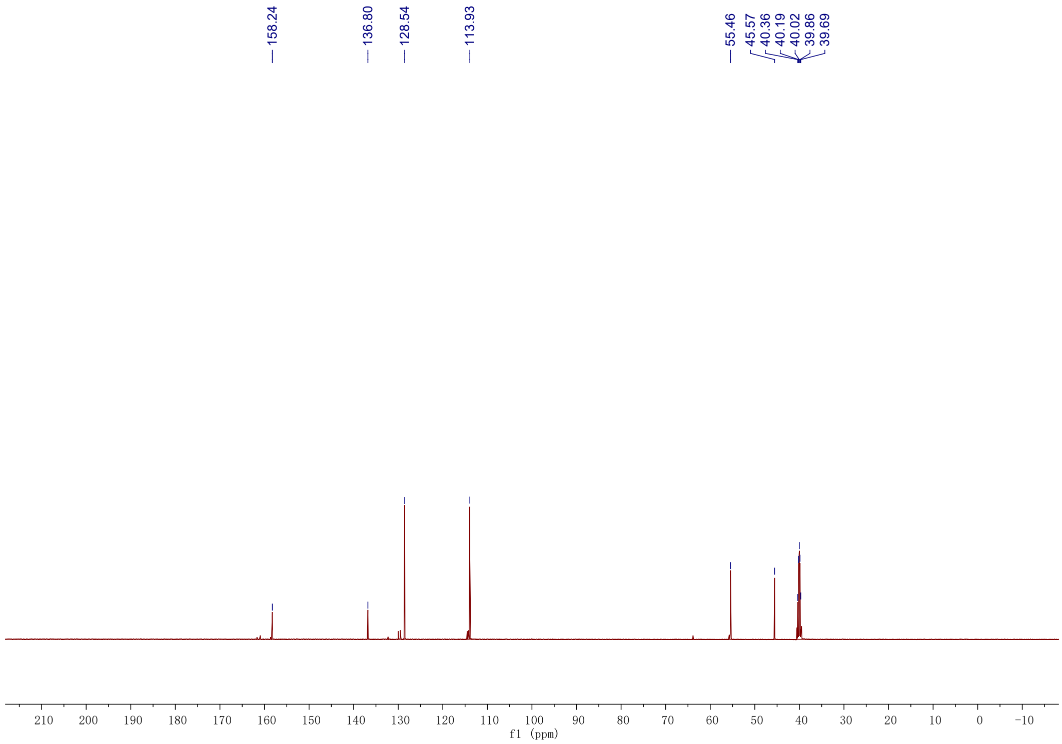

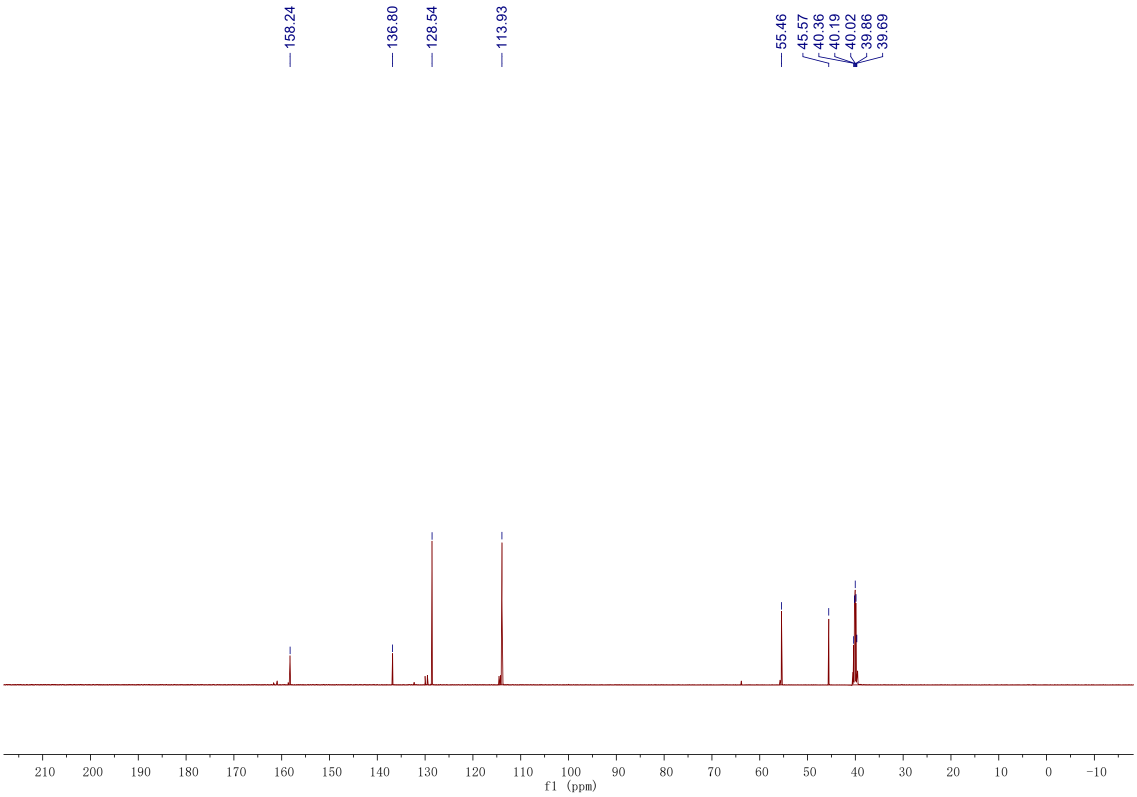

(3,4-Dimethoxyphenyl)methanamine

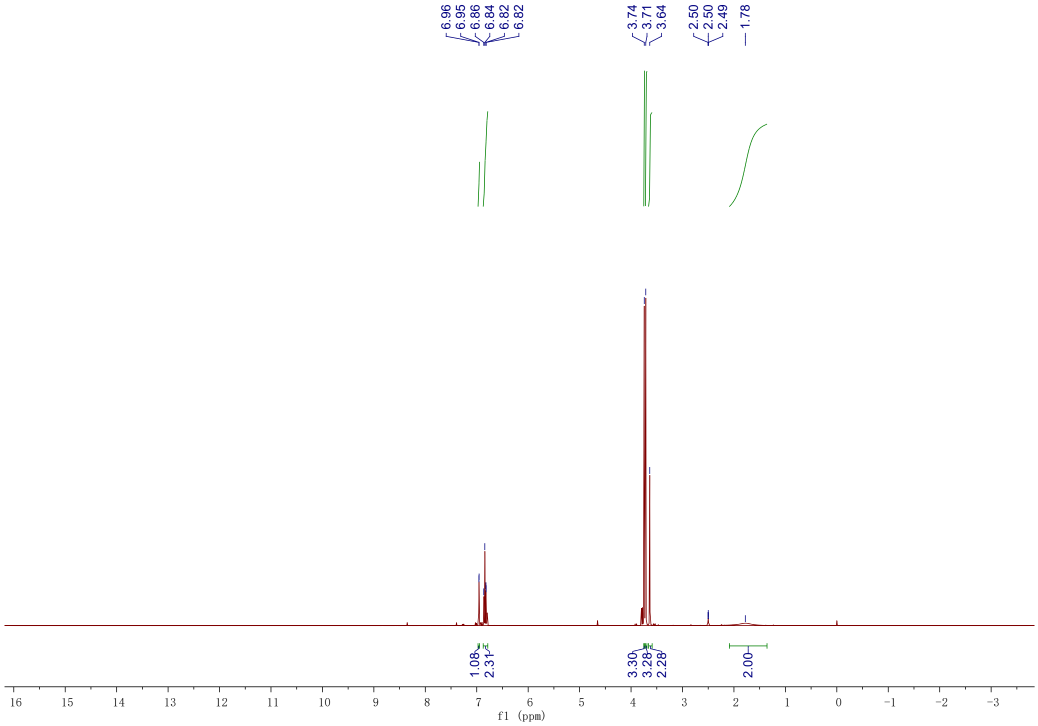

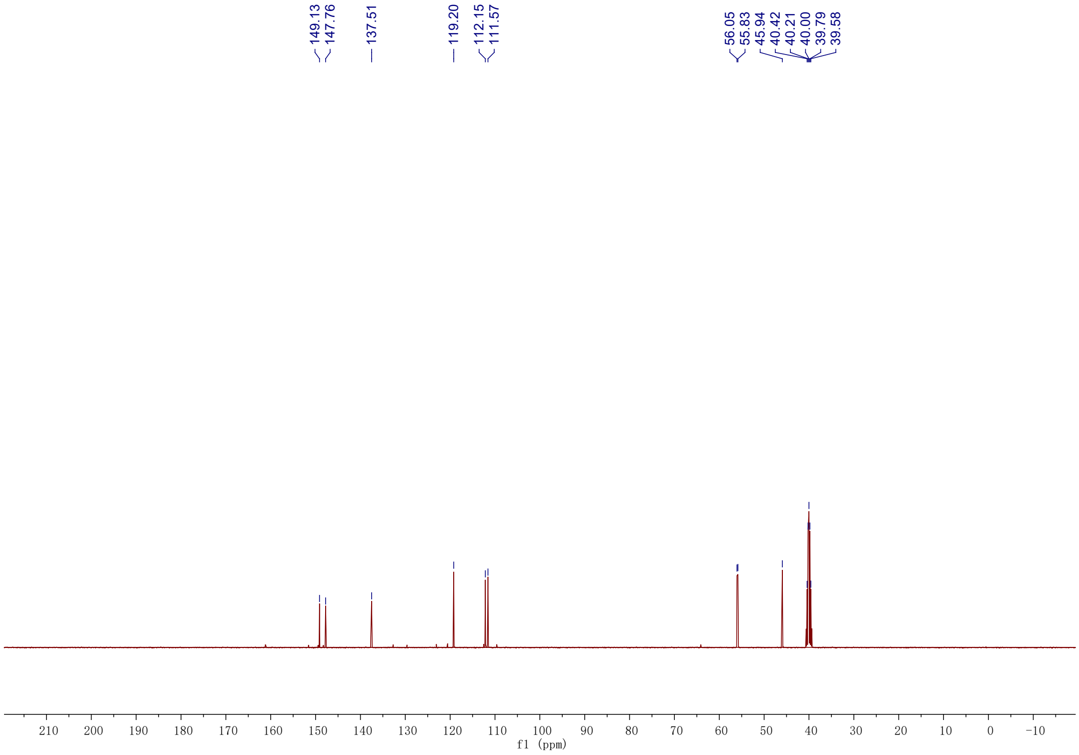

N-(4-(aminomethyl)phenyl)acetamide

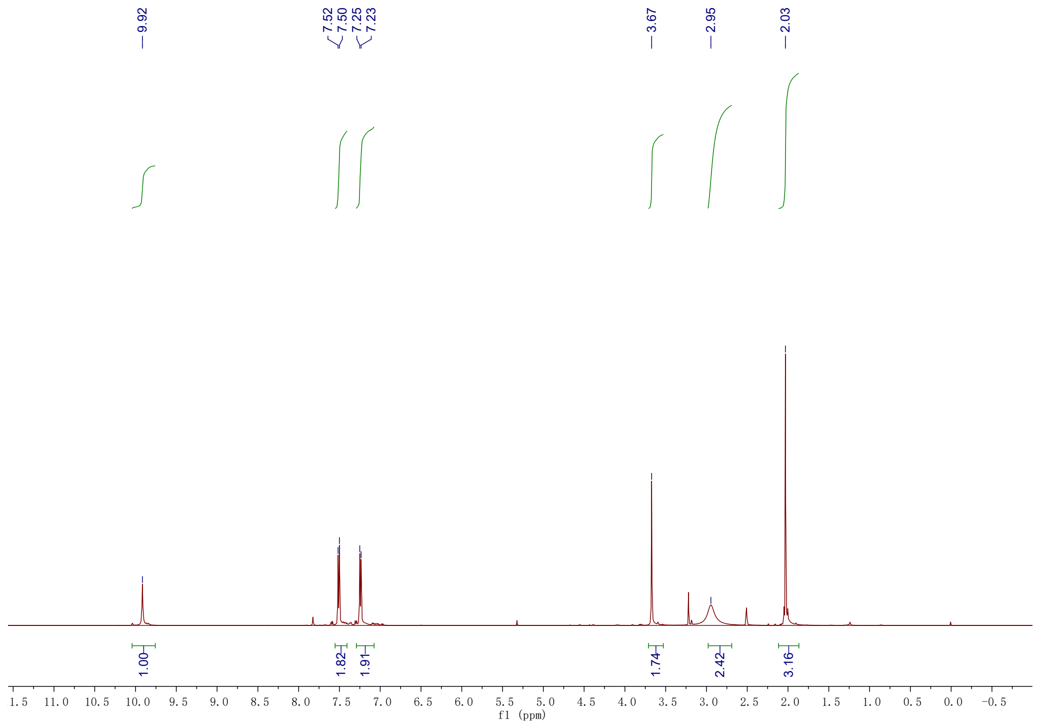

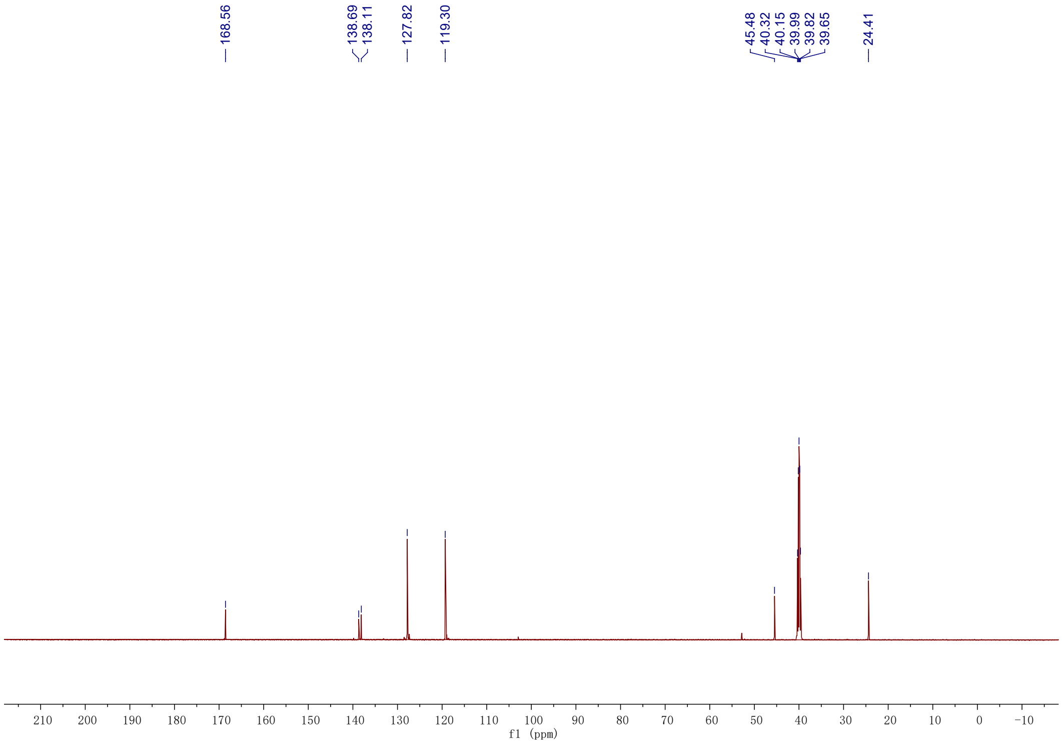

Methyl 4-(aminomethyl)benzoate

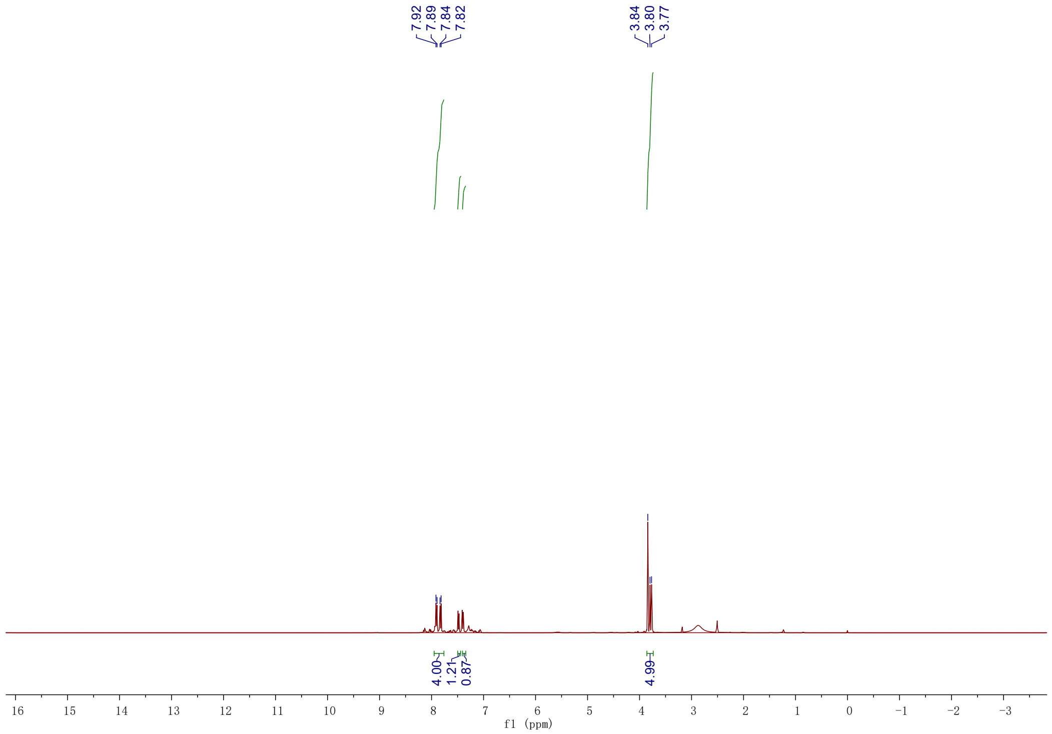

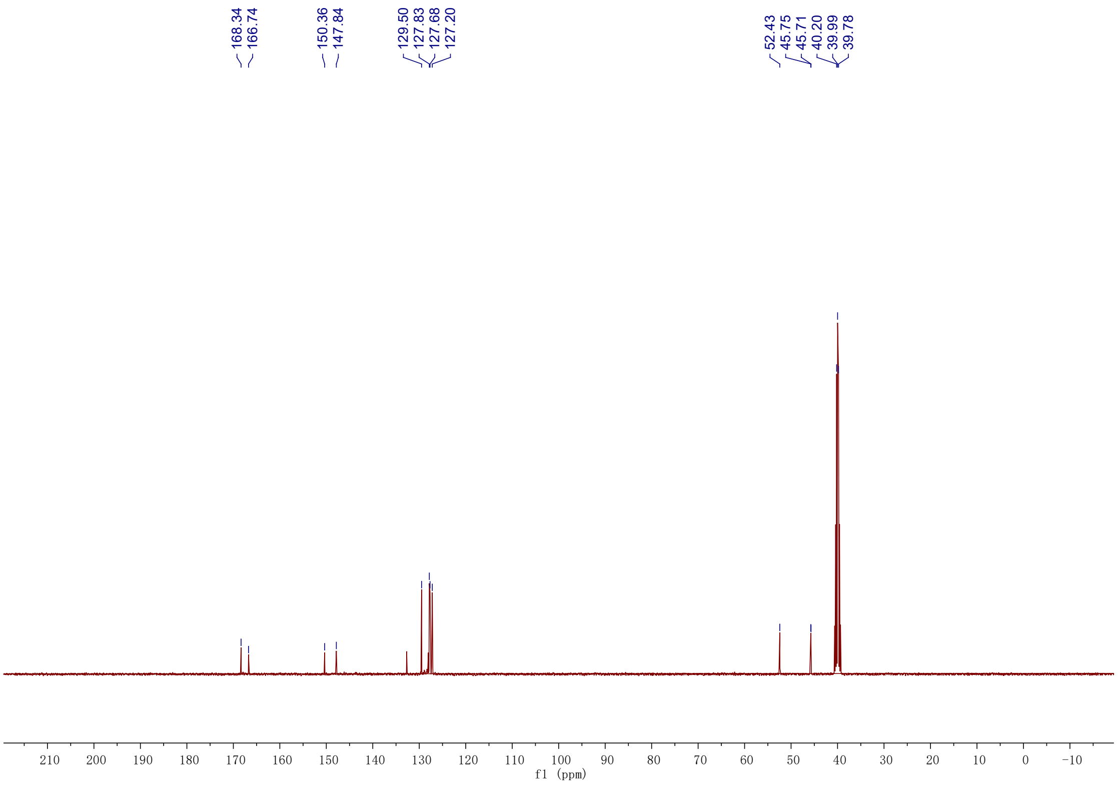

3,7-dimethyloct-6-en-1-amine

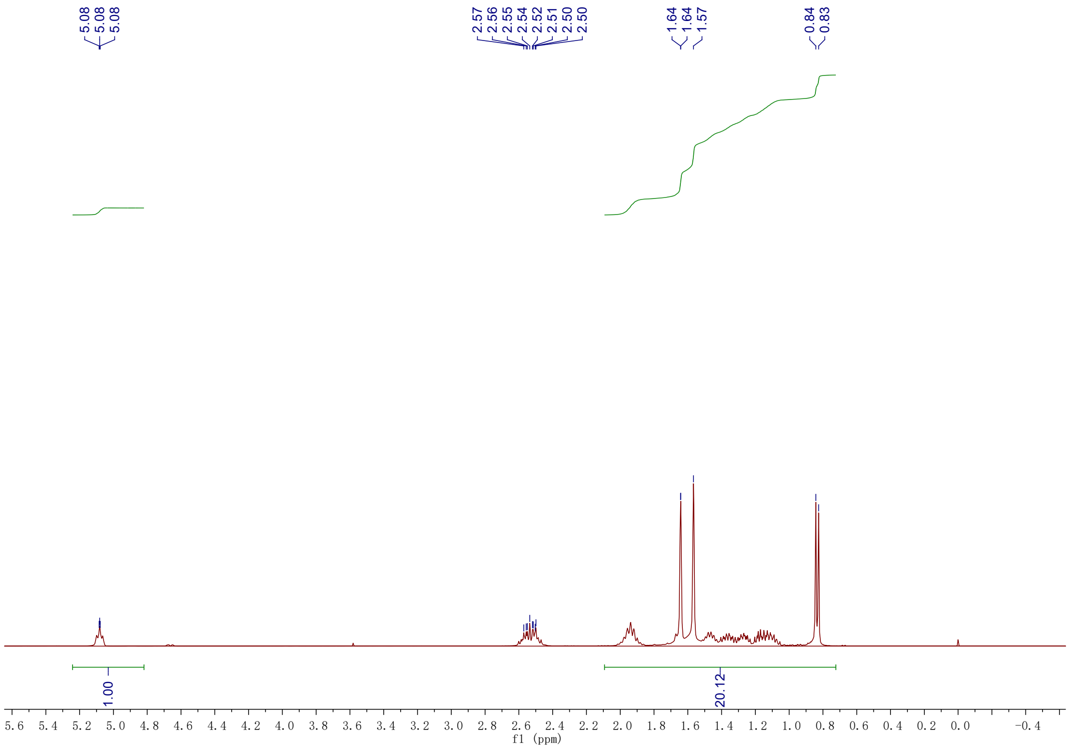

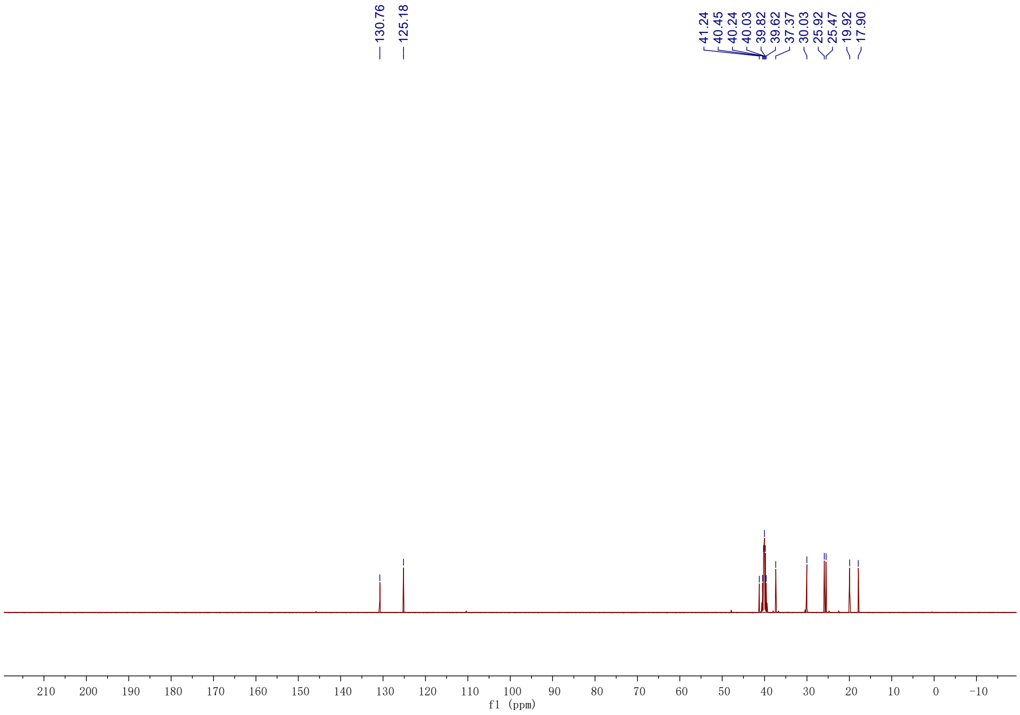

(5-(Aminomethyl)furan-2-yl)methanol

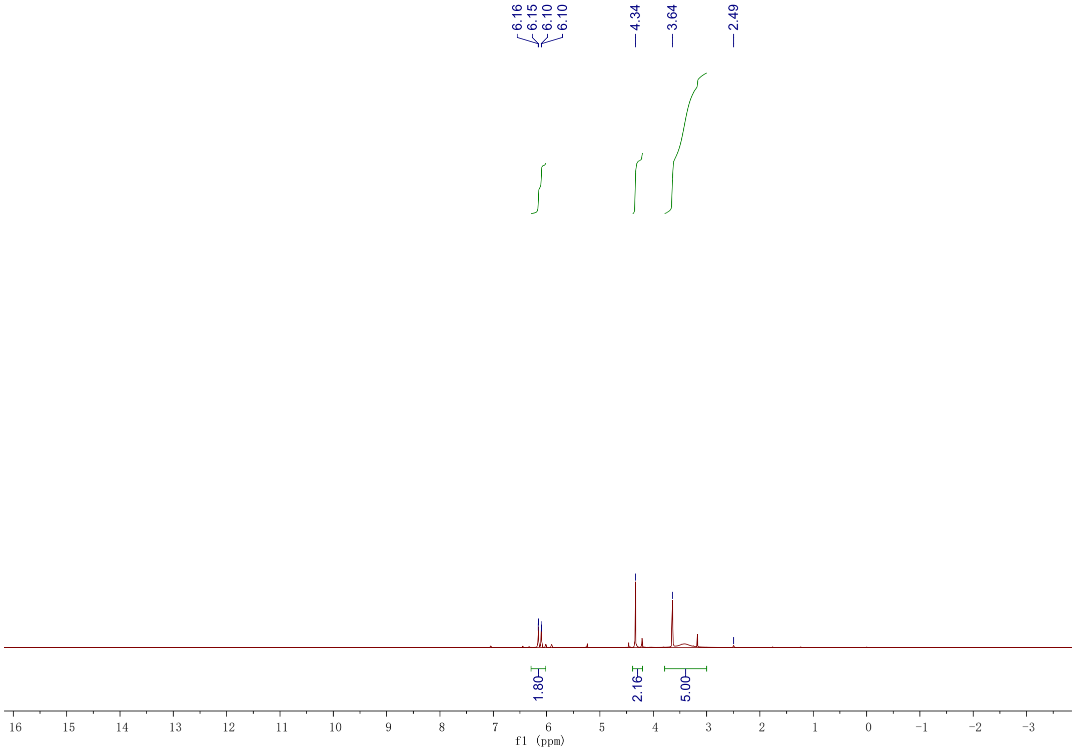

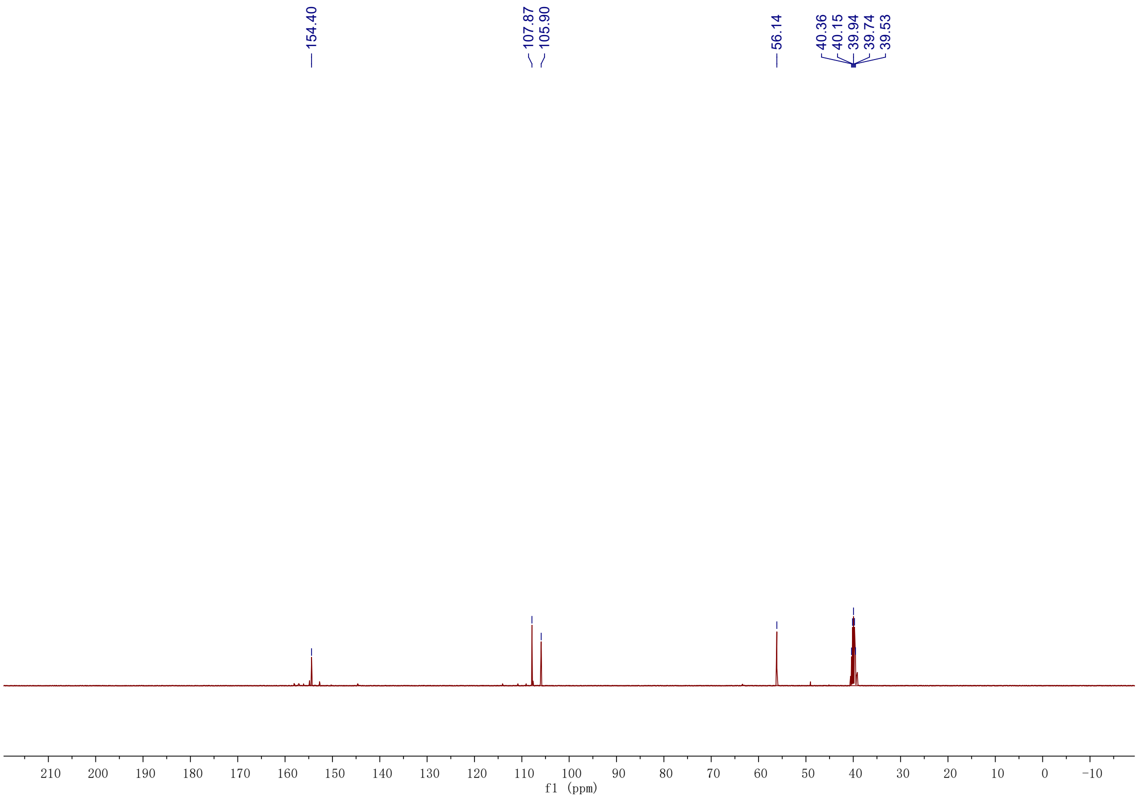

Androsterone-NH_2 (diastereomeric mixture, 66:34)

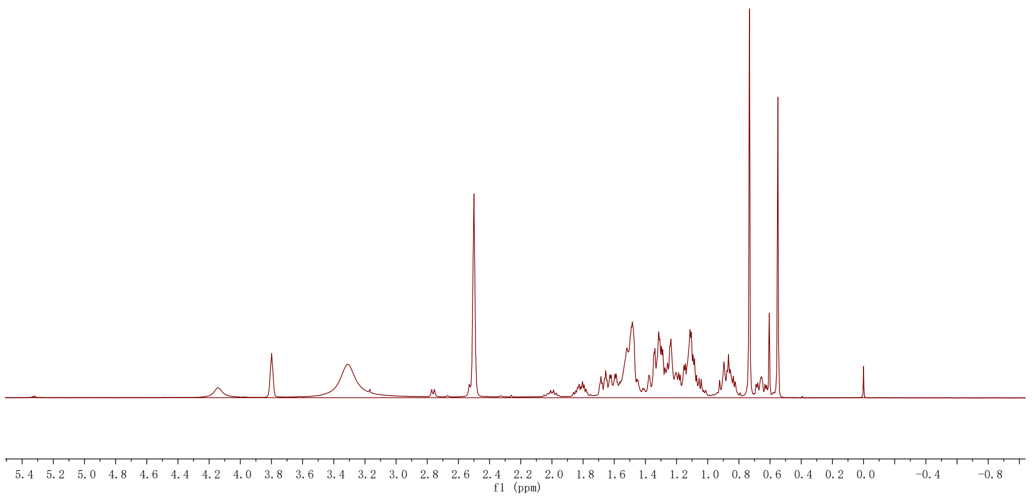

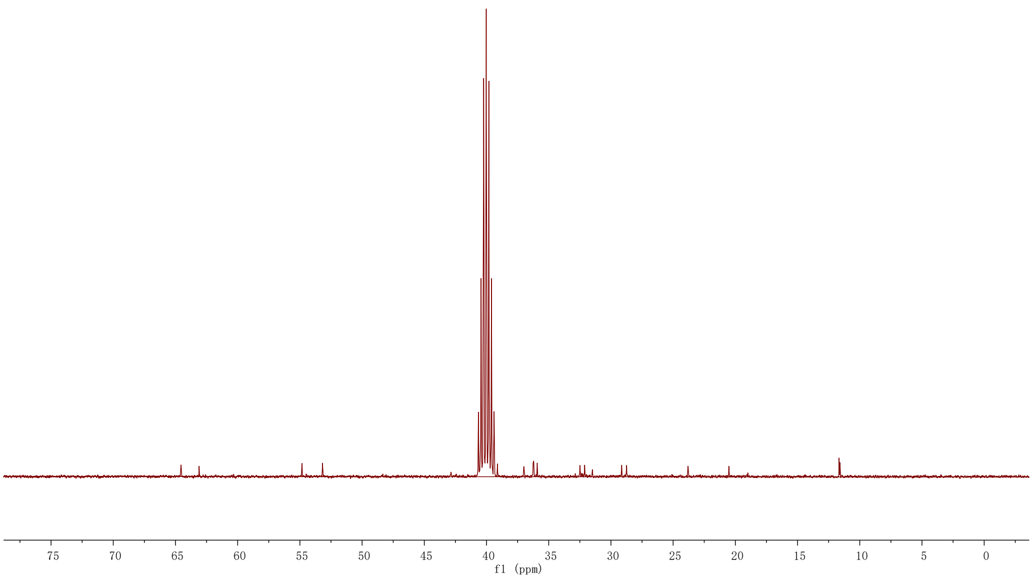

Supplement: SC-013-D2SC01596J-s001 [file SC-013-D2SC01596J-s001.pdf]
